# Chromosomal Inversions and the Demography of Speciation in *Drosophila montana* and *Drosophila flavomontana*

**DOI:** 10.1093/gbe/evae024

**Published:** 2024-03-14

**Authors:** Noora Poikela, Dominik R Laetsch, Ville Hoikkala, Konrad Lohse, Maaria Kankare

**Affiliations:** Department of Biological and Environmental Science, University of Jyväskylä, FI-40014, Jyväskylä, Finland; Institute of Evolutionary Biology, University of Edinburgh, Edinburgh, UK; Department of Biological and Environmental Science, University of Jyväskylä, FI-40014, Jyväskylä, Finland; Institute of Evolutionary Biology, University of Edinburgh, Edinburgh, UK; Department of Biological and Environmental Science, University of Jyväskylä, FI-40014, Jyväskylä, Finland

**Keywords:** chromosomal inversion, coalescence, *Drosophila*, genetic divergence, introgression, speciation

## Abstract

Chromosomal inversions may play a central role in speciation given their ability to locally reduce recombination and therefore genetic exchange between diverging populations. We analyzed long- and short-read whole-genome data from sympatric and allopatric populations of 2 *Drosophila virilis* group species, *Drosophila montana* and *Drosophila flavomontana*, to understand if inversions have contributed to their divergence. We identified 3 large alternatively fixed inversions on the X chromosome and one on each of the autosomes 4 and 5. A comparison of demographic models estimated for inverted and noninverted (colinear) chromosomal regions suggests that these inversions arose before the time of the species split. We detected a low rate of interspecific gene flow (introgression) from *D. montana* to *D. flavomontana*, which was further reduced inside inversions and was lower in allopatric than in sympatric populations. Together, these results suggest that the inversions were already present in the common ancestral population and that gene exchange between the sister taxa was reduced within inversions both before and after the onset of species divergence. Such ancestrally polymorphic inversions may foster speciation by allowing the accumulation of genetic divergence in loci involved in adaptation and reproductive isolation inside inversions early in the speciation process, while gene exchange at colinear regions continues until the evolving reproductive barriers complete speciation. The overlapping X inversions are particularly good candidates for driving the speciation process of *D. montana* and *D. flavomontana*, since they harbor strong genetic incompatibilities that were detected in a recent study of experimental introgression.

**Significance**

Chromosomal inversions, genomic rearrangements with reversed gene order, have been extensively studied, but it remains unclear whether and how inversions play a role in species divergence. Analysis of long- and short-read whole-genome data for 2 *Drosophila* sister species, *Drosophila montana* and *Drosophila flavomontana*, revealed 5 alternatively fixed inversions. Modeling the demographic history of these inversions shows that they were segregating already in the common ancestor of the species and that they have reduced gene exchange between these sister taxa both before and after the onset of species divergence. These results are compatible with a scenario in which ancestrally polymorphic inversions aid species divergence by protecting divergently selected loci from erosion via gene flow during the earliest stages of speciation.

## Introduction

Chromosomal inversions, genomic regions with reversed gene order, may facilitate adaptation and speciation in the face of gene flow because they suppress recombination between alternate rearrangements, which creates and preserves associations between sets of alleles conferring local adaptation, mate choice, and genetic incompatibilities (Sturtevant 1921; Butlin 2005; Hoffmann and Rieseberg 2008). While inversions have been found in many species of insects, fish, birds, mammals, and plants, their frequency varies widely between and even within taxa (Stone et al. 1960; Wellenreuther and Bernatchez 2018), and it remains an open question whether and how inversions contribute to the evolution of species divergence. Genomic data from young species pairs offer the chance to reconstruct both the demographic history of species divergence in the face of gene flow and the history of alternatively fixed inversions and interspecific gene flow (introgression) (Faria et al. 2018; Faria and Navarro 2010).

Inversions may facilitate adaptation and speciation in many ways (reviewed in Hoffmann and Rieseberg 2008; Jackson 2011; Faria et al. 2018). A new inversion may be favored by selection if it protects epistatic interactions (Hoffmann and Rieseberg 2008) and/or locally adapted alleles (Kirkpatrick and Barton 2006) from recombination with immigrant alleles that reside in an alternate rearrangement. Also, an inversion may be under selection if its breakpoints disrupt reading frames of genes or change the expression of genes (Wright and Schaeffer 2022; Matzkin et al. 2005; Villoutreix et al. 2021). While the probability of fixation of an inversion between diverging populations depends on the strength of selection and the levels of gene flow (Hoffmann and Rieseberg 2008), its potential to contribute to local adaptation and/or speciation in the long term depends also on whether populations evolve in isolation or in the face of gene flow. Upon secondary contact, alternatively fixed inversions may protect existing incompatibilities from gene flow between diverging populations, while noninverted (colinear) regions are more susceptible to the homogenizing effects of gene flow (Noor et al. 2001). In contrast, if populations diverge in the presence of gene flow, we expect incompatibilities to accumulate in inverted regions (Navarro and Barton 2003). In both scenarios, inversions harboring incompatibilities delay species’ fusion and provide time for additional barriers to evolve. For example, prezygotic reproductive barriers are expected to be more easily reinforced in response to genetic incompatibilities and maladaptive hybridization (reinforcement) (Servedio and Noor 2003), if the causal loci are located within inversions (Trickett and Butlin 1994; Butlin 2005; Dagilis and Kirkpatrick 2016). Two kinds of empirical observations give indirect support for these theories. First, genes maintaining local adaptation, premating barriers, and genetic incompatibilities between species have been found to be concentrated in alternatively fixed inversions (Fishman et al. 2013; Lowry and Willis 2010; Noor et al. 2001). Second, fixed inversions generally have elevated genetic divergence compared to colinear regions (Noor et al. 2007; Kulathinal et al. 2009; Lohse et al. 2015). However, it has proven extremely difficult to distinguish speciation histories in which inversions have acted as triggers of speciation from scenarios in which alternately fixed inversions arise incidentally either because they are polymorphic in the ancestral population for reasons that may have nothing to do with local adaptation (Fuller et al. 2019; Faria and Navarro 2010; Guerrero and Hahn 2017) or because they arise after speciation is complete.

So far, only a few studies have dissected the evolutionary history of inversions to explore their role in adaptation (Lundberg et al. 2023) and speciation (e.g. Lohse et al. 2015; Fuller et al. 2018). Demographic models can be used to systematically compare the species’ divergence time estimated from colinear regions (*T*_col_) and the origin of inversions (*T*_inv_) and the amount of long-term effective introgression between inverted (*M*_inv_) and colinear (*M*_col_) regions (Noor and Bennett 2009). Similarly, recent or ongoing introgression can be diagnosed by comparing estimates of *M* between sympatric and allopatric population pairs (Noor and Bennett 2009). There are at least 3 scenarios for the evolutionary history of alternately fixed inversions. First, inversions arise and fix after speciation is largely complete, most likely for reasons unrelated to the speciation process. In this case, we expect reduced introgression (*M*_inv_ < *M*_col_) within inversions, but the same split time estimates for inversions and colinear regions (*T*_inv_ = *T*_col_). Second, inversions fix during the speciation process because they contribute to local adaptation and/or formation of reproductive isolation at an early stage of high gene flow (Kirkpatrick and Barton 2006). Such inversions should have reduced introgression compared to colinear regions (*M*_inv_ < *M*_col_) and their estimated divergence time either predates that of colinear regions (*T*_inv_ > *T*_col_) (if they have been segregating in the common ancestral population) or is the same (*T*_inv_ = *T*_col_) (if they arose at the onset of divergence). Crucially, however, irrespective of their age, we expect that these inversions have fixed because they act as barriers to gene flow; i.e. they protect alleles that are involved in local adaptation, mate choice, and/or genetic incompatibilities. Finally, in a third scenario, inversions are segregating in the ancestral population due to forces that have nothing to do with local adaptation or speciation. Importantly, we would expect any inversion that segregates in the ancestral population to be alternately fixed between the 2 species by chance alone with a probability of 1/2 (Guerrero and Hahn 2017). Such coincidental inversions that fix differentially with no effect on species divergence could still help impede species fusion upon secondary contact if they contain Bateson-Dobzhansky-Muller incompatibilities (BDMIs) (Noor et al. 2001). However, the predictions for the coincidental inversion scenario in terms of demographic parameters are the same as in the second scenario above.

**Fig. 1.**
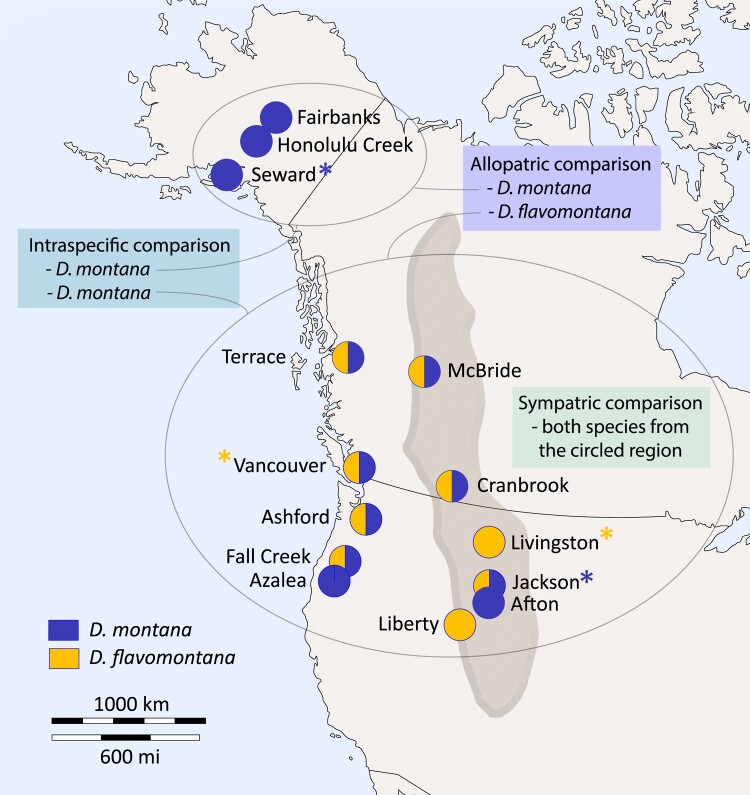
Sampling sites of sympatric (or parapatric) and allopatric *D. montana* and *D. flavomontana* populations in North America. Pie charts indicate the sampling sites for 1 or both species. Long-read PacBio data were obtained from 2 isofemale strains per species (sample sites indicated with asterisks). Short-read Illumina data were obtained from single wild-caught females for all sites shown. The dark area illustrates the Rocky Mountains of North America. The map template was obtained from https://d-maps.com/carte.php?num_car=5082.

The 2 *Drosophila virilis* group species, *Drosophila montana* and *Drosophila flavomontana*, offer a great opportunity to investigate the potential role of inversions in species divergence. Based on polytene chromosome studies, these species have several alternatively fixed inversions (Throckmorton 1982; Stone et al. 1960), which, however, have so far not been characterized at the genomic level. *D. montana* and *D. flavomontana* have diverged ∼3.8 Mya in the Rocky Mountains, and the 2 species presently inhabit variable climates in the Rocky Mountains and along the western coast of North America (Hoikkala and Poikela 2022; Yusuf et al. 2022). In the mountains, *D. montana* has spread to higher altitudes than *D. flavomontana*, while on the western coast, where *D. flavomontana* has expanded relatively recently, both species live at low altitudes (Fig. 1; supplementary table S1, Supplementary Material online; Patterson 1952; Hoikkala and Poikela 2022). Thus, in both regions, populations of the 2 species can be regarded as sympatric or parapatric. However, *D. montana* also has allopatric populations at high latitudes, e.g. in Alaska, where *D. flavomontana* does not exist (Fig. 1; supplementary table S1, Supplementary Material online). Reproductive isolation between *D. montana* females and *D. flavomontana* males is nearly complete, characterized by an extremely strong prezygotic isolation and inviability and sterility of F_1_ females and males (Poikela et al. 2019). In contrast, prezygotic isolation between *D. flavomontana* females and *D. montana* males is relatively weaker and shows signs of reinforcement in sympatric populations of *D. flavomontana* (Poikela et al. 2019). Furthermore, in these crosses, F_1_ hybrid males are sterile but F_1_ hybrid females can be crossed with males of both parental species to obtain backcross progenies in both directions (Poikela et al. 2019, 2023). Importantly, evidence for strong BDMI(s) between these species located within inversions on the X chromosome has been found (Poikela et al. 2023). This prevents introgression from *D. montana* to *D. flavomontana* across the entire X chromosome during early backcross generations (Poikela et al. 2023). Despite the strong reproductive isolation, interspecific hybrids have been found in nature (Patterson 1952; Throckmorton 1982).

Here, we explored whether and how inversions have contributed to the species divergence of *D. montana* and *D. flavomontana*. We used long- and short-read sequencing data from allopatric and sympatric populations of the species to generate highly contiguous assemblies for both species, which in turn enabled us to accurately identify the presence of alternatively fixed inversions. We used demographic modeling to estimate the age of these inversions and their potential effect on the long-term rate of introgression and asked the following specific questions:

1. How many alternatively fixed inversions do *D. montana* and *D. flavomontana* carry?
2. When did these inversions most likely arise and how does their age compare to the species divergence time?
3. Do these inversions show reduced introgression compared to colinear regions as would be expected if they arose during or before the onset of species divergence?

## Results and Discussion

We generated long-read Pacific Biosciences (PacBio) sequencing data for females from 2 *D. montana* and 2 *D. flavomontana* isofemale strains and short-read Illumina resequencing data for 12 *D. montana* and 9 *D. flavomontana* wild-caught females (1 female per population per species) originating from allopatric and sympatric populations (Fig. 1; supplementary tables S1 and S2, Supplementary Material online). These data enabled us to generate contiguous, high-quality genome assemblies for both species to accurately identify alternatively fixed inversions and to examine the species’ evolutionary history and the role of inversions and introgression therein. In the following, we refer to the comparison between *D. montana* and *D. flavomontana* samples from the Rocky Mountains and the western coast as “sympatric” and the comparisons between *D. montana* from Alaska and *D. flavomontana* from the mountains and the coast as “allopatric” (Fig. 1; supplementary table S1, Supplementary Material online). To evaluate the timing of potential recent introgression in sympatry, we estimated the divergence time for *D. montana* living in contact (sympatry) and in isolation (allopatry) with *D. flavomontana*, and we refer to this comparison as “intraspecific” (Fig. 1; supplementary table S1, Supplementary Material online).

### Construction and Annotation of Species Genomes

Two genome assemblies for each species were generated using the PacBio data of 2 *D. montana* and *D. flavomontana* isofemale strains and the Illumina data for the respective founder females collected from the wild (supplementary tables S1 and S2, Supplementary Material online). The assembled genomes had a total length of 181 to 194 Mb (supplementary table S3, Supplementary Material online), which resemble those of previously assembled *D. montana*, *D. flavomontana*, and several other *Drosophila* species (128 to 198 Mb) (Miller et al. 2018; Parker et al. 2018; Yusuf et al. 2022). A small proportion of each assembly (0 to 18 contigs, spanning = 0.0 to 9.9 Mb) was excluded as contaminant sequences, mainly bacteria, based on the coverage, GC%, and taxonomic annotation of contigs (supplementary figs. S1 to S4, Supplementary Material online). From the 3,285 BUSCO groups, we identified 97.3% to 98.5% as complete BUSCOs, of which 96.9% to 98.0% were single-copy and 0.4% to 0.5% duplicated BUSCOs (supplementary table S3, Supplementary Material online). The BUSCO values were similar to the ones in other *Drosophila* assemblies (Miller et al. 2018). Repetitive sequences comprised 25.5% to 29.9% of our assemblies (supplementary table S3, Supplementary Material online), which is close to the repeat content reported for other *Drosophila* species (e.g. 26.5% in *D. virilis*, 28.6% in *Drosophila melanogaster*, 22.9% in *Drosophila mojavensis*, and 19.9% in *Drosophila elegans*; NCBI Annotation Report). Our annotations included 15,696 to 16,056 genes per assembly, which is plausible given the number of genes reported for other *Drosophila* assemblies (e.g. Yang et al. 2018). Overall, the combination of long- and short-read data resulted in more contiguous assemblies for both species (N50 values of 1.3 to 11.0 Mb; supplementary table S3, Supplementary Material online) compared to the previously published *D. montana* and *D. flavomontana* genomes that were based on short-read data (e.g. N50 of 41 kb in *D. montana*; Parker et al. 2018; Yusuf et al. 2022).

We built a chromosome-level reference genome for *D. montana* by scaffolding with the genome of another *virilis* group species, *Drosophila lummei*, and for *D. flavomontana* by first scaffolding 1 assembly with the other (within species) and then with the *D. lummei* genome (see Materials and Methods for details). For both chromosome-level genomes, the total genome size, BUSCO values, and the number of repeats and genes slightly decreased compared to the original, nonscaffolded assemblies (supplementary table S3, Supplementary Material online). Given greater span and completeness (as measured by BUSCO) of the *D. montana* compared to the *D. flavomontana* genome, subsequent analyses were performed using *D. montana* as a reference by default. However, to quantify the effect of reference bias, we repeated the demographic inference using *D. flavomontana* as a reference.

**Fig. 2.**
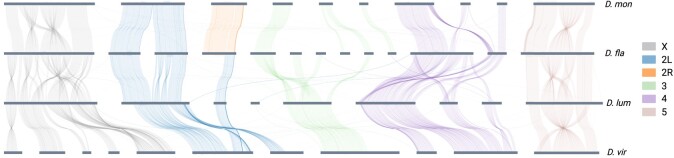
Chromosome synteny between *D. montana* (*D. mon*: monSE13F37), *D. flavomontana* (*D. fla*: flaMT13F11), *D. lummei* (*D. lum*), and *D. virilis* (*D. vir*). Different chromosomes and chromosome arms are marked with different colors, in the order displayed in the legend. The plot shows contigs larger than 2 Mb.

To understand how chromosomes of *D. montana* and *D. flavomontana* relate to the more studied *D. virilis*, we compared the genomes of *D. montana* and *D. flavomontana* (species of the *montana* phylad of the *virilis* group) and *D. virilis* and *D. lummei* (species of the *virilis* phylad of the *virilis* group) (Yusuf et al. 2022). While chromosome synteny is highly variable between distantly related *Drosophila* species, such as *D. melanogaster* and *D. virilis* (Schaeffer et al. 2008), it is relatively similar between the *virilis* group species (Fig. 2; Stone et al. 1960). The most noticeable difference is that in *D. montana* and *D. flavomontana*, chromosome 2 has left (2L) and right (2R) arms that are separated by a (sub)metacentric centromere, while in *D. virilis* and *D. lummei*, the centromere is located near 1 end of the chromosome 2 (Fig. 2; Stone et al. 1960).

### Genetic Differentiation and Climatic Variability of *D. montana* and *D. flavomontana* Populations

**Fig. 3.**
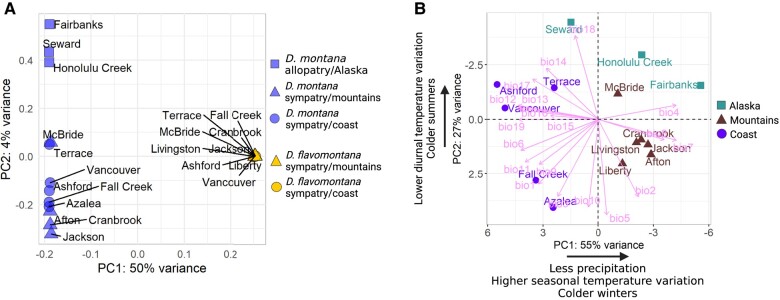
A PCA A) on whole-genome SNP data of *D. montana* and *D. flavomontana* Illumina resequence samples originating from different sites of North America and B) on 19 bioclimatic variables (detailed explanations in supplementary table S5, Supplementary Material online) of each fly sampling site.

To investigate the genetic structure of *D. montana* and *D. flavomontana* populations, we performed a principal component analysis (PCA) on the Illumina resequence data for the 12 and 9 wild-caught females of *D. montana* and *D. flavomontana*, respectively (supplementary tables S1 and S2, Supplementary Material online). The PCA included 9,102,309 filtered single nucleotide polymorphisms (SNPs) from coding, intronic, and intergenic regions. The first 2 principal components (PCs) had Eigenvalues of >1, and PC1 explained majority (50%) of the total genetic variance and clearly separated *D. montana* and *D. flavomontana* samples from each other (Fig. 3A; supplementary table S4, Supplementary Material online). PC2 explained 4% of the total variance and captured variation mainly within *D. montana*, while variation in *D. flavomontana* was lower (Fig. 3A; supplementary table S4, Supplementary Material online). PC2 separated allopatric Alaskan *D. montana* populations (Honolulu Creek, Seward, and Fairbanks) from sympatric mountainous and coastal *D. montana* populations and also showed some variation within the allopatric and sympatric populations (Fig. 3A; supplementary table S4, Supplementary Material online).

Next, we explored climatic variability of fly sampling sites to determine the extent to which climatic conditions may have affected the genetic differentiation of the samples. We performed a PCA on 19 bioclimatic variables of each fly sampling site (supplementary tables S5 and S6, Supplementary Material online) to reduce correlations between the variables and summarized climatic patterns prevailing in the sites. This PCA revealed 3 PCs with Eigenvalues of >1, of which the first 2 PCs explained ∼80% of the climatic variation (Fig. 3B; supplementary table S7, Supplementary Material online). The first PC clearly separated inland and coastal populations and suggested that populations from the mountainous inland experience cold winters and high seasonal temperature variation, while coastal populations experience milder temperatures and high precipitation throughout the year (Fig. 3B; supplementary table S8, Supplementary Material online). The second PC separated populations based on summer temperatures and variation in diurnal temperatures and distinguished Alaskan populations (Honolulu Creek, Seward, Fairbanks) from the other populations (Fig. 3B; supplementary table S8, Supplementary Material online).

Together, these results show that *D. montana* and *D. flavomontana* populations are genetically diverged regardless of their climatic origin and species coexistence. Genetic differentiation was greater among *D. montana* populations than among *D. flavomontana* populations, which is likely due to *D. montana*'s larger geographic range and the fact that *D. flavomontana* has spread across North America more recently than *D. montana* (Hoikkala and Poikela 2022). Finally, the genetic differentiation between allopatric (from Alaska) and sympatric (from the Rocky Mountains and the western coast) *D. montana* populations likely reflects a demographic history of intraspecific divergence, local adaptation to climatic conditions, or both.

### *D. montana* and *D. flavomontana* Chromosomes Differ by Several Large Inversions

We combined long- and short-read genomic data to characterize inversion breakpoints in *D. montana* and *D. flavomontana*. We identified 5 large (>0.5 Mb) inversions that were alternatively fixed between North American *D. montana* and *D. flavomontana* samples (supplementary table S9, Supplementary Material online; Fig. 2; supplementary figs. S5 to S10, Supplementary Material online). The X chromosome contained 3 partly overlapping inversions, one of which was fixed in *D. montana* (7.2 Mb) and 2 in *D. flavomontana* (11.0 and 3.1 Mb) (supplementary table S9 and figs. S5 to S10, Supplementary Material online). Chromosomes 4 and 5 each contained 1 inversion fixed in *D. flavomontana* (15.9 and 9.2 Mb, respectively) (supplementary table S9 and fig. S5, Supplementary Material online). All these inversions were homozygous in Illumina resequenced individuals of both species (supplementary table S9, Supplementary Material online). In contrast, chromosomes 2 and 3 did not contain any fixed inversion differences. However, a subset of reads indicated that the left arm of the second chromosome (2L) contained an inversion (3.9 Mb) that was heterozygous in all *D. montana* samples (supplementary table S9 and fig. S5, Supplementary Material online). Since this inversion signal is derived solely from raw reads and not from genome comparisons, we cannot exclude the possibility that this is a false positive. Because this putative inversion is not fixed between the species, it was excluded from further analysis. The sizes of inversions were obtained from the genome assemblies of each species (supplementary table S9, Supplementary Material online). Overall, repeat density was higher at 4 of the 10 breakpoints compared to the mean values for the X chromosome and autosomes (supplementary fig. S11, Supplementary Material online). Generally, high abundance of repeats may contribute to the origin of inversions (Kapun and Flatt 2019). Intriguingly, a known TE (Mariner-2_DVi) was found at the distal breakpoint of the shorter fixed X inversion in *D. flavomontana* but not in *D. montana* genomes, which could potentially be associated with the establishment of that inversion (supplementary file S1, Supplementary Material online). PacBio read support (ranging between 16 and 106 reads) and genes and repetitive regions located at inversion breakpoints are shown in supplementary file S1, Supplementary Material online.

Based on polytene chromosome studies (Stone et al. 1960), the 3 alternatively fixed inversions between *D. montana* and *D. flavomontana* on the X chromosome likely correspond to inversions E, F, and G. These inversions were not distinguished in more detail in Stone et al. (1960), and, in contrast to our results, Stone et al. (1960) suggest that all 3 X inversions are fixed in *D. flavomontana*. The inversions on the fourth and fifth chromosome have been named J and E in karyotype studies, respectively (Stone et al. 1960).

The average size of the inversions fixed in *D. montana* was 7.2 Mb and in *D. flavomontana* 9.8 Mb (supplementary table S9, Supplementary Material online), which resembles the average reported size of inversions in animals and plants (8.4 Mb) (Wellenreuther and Bernatchez 2018). Our finding of a larger number of inversions on the X is consistent with theory showing that the fixation probability of X chromosomal inversions is higher than that of autosomal inversions, because selection can more effectively favor beneficial and purify deleterious recessive X-linked alleles than autosomal ones (Charlesworth et al. 2018, 1987; Connallon et al. 2018; Vicoso and Charlesworth 2006). Moreover, the higher content of repetitive sequences we find on the X chromosome compared to autosomes (supplementary fig. S11, Supplementary Material online), which has also been observed in other *Drosophila* species (Cridland et al. 2013), may predispose the X chromosome to sequence breakage and thus facilitate the formation of inversions (Kapun and Flatt 2019).

The polytene chromosome studies by Stone et al. (1960) and Throckmorton (1982) suggest that *D. montana* and *D. flavomontana* carry additional inversions that were not detected in this study. In particular, *D. flavomontana* may harbor 1 fixed inversion of unknown size on chromosome 3 (inversion E; Stone et al. 1960), which we might have missed due to the higher fragmentation of this chromosome compared to the other chromosome contigs (supplementary table S10, Supplementary Material online). Given our limited sample size and explicit focus on fixed inversion differences between species, polymorphic inversions previously found in these species (Stone et al. 1960), which may be associated with local adaptation or other evolutionary processes (Fang et al. 2012; Kapun et al. 2016; Wallberg et al. 2017), were also not identified here.

### Genetic Divergence between *D. montana* and *D. flavomontana* Is Greater Inside Than Outside Inversions

We analyzed the mean genetic divergence (*d_xy_*) to test whether inversions have reduced recombination and introgression between *D. montana* and *D. flavomontana*, and if so, whether this is ancient or recent. In the latter case, *d_xy_* should be lower in sympatry compared to allopatry (Harrison and Larson 2014; Noor and Bennett 2009). We estimated *d_xy_* separately for coding, intronic, and intergenic sequences and inverted and colinear regions of the genome. Given the potentially different evolutionary history of the X chromosome and the autosomes (Charlesworth et al. 2018; Vicoso and Charlesworth 2006), analyses for the X were performed separately. We also carried out separate analyses for allopatric and sympatric comparisons of the species. We focus mainly on the absolute measure of genetic divergence (*d_xy_*), since relative differentiation, i.e. *F*_ST_, measures both variation in genetic diversity and divergence and so is harder to interpret (Cruickshank and Hahn 2014; Charlesworth 1998; Noor and Bennett 2009).

**Fig. 4.**
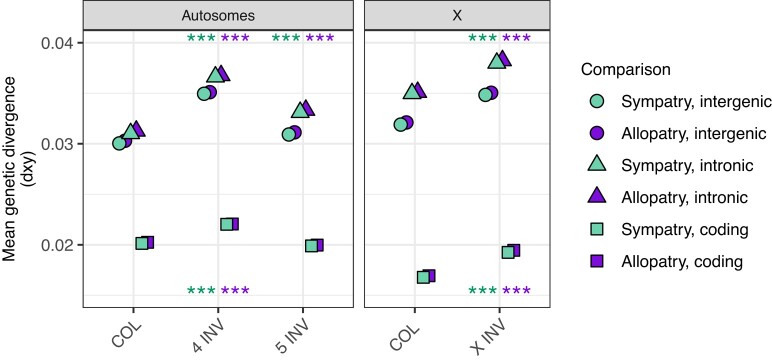
Mean genetic divergence (measured as *d_xy_*) at intergenic, intronic, and coding sequences of colinear (COL, background) and inverted (INV) chromosome partitions on the autosomes and the X. Divergence is shown for allopatric (dark purple) and sympatric (light green) comparisons of *D. montana* and *D. flavomontana*. Significance levels were inferred from simulations, where COL regions were compared to INV regions separately for autosomes and the X chromosome, for intergenic, intronic, and coding sequences, and for allopatric and sympatric comparisons (***P < 0.001; *P*-values for intergenic and intronic sequences shown above and for coding sequences below dots; supplementary table S11, Supplementary Material online).

Mean divergence (*d_xy_*) between *D. montana* and *D. flavomontana* was remarkably similar for intergenic and intronic sequences but much lower for coding sequences (Fig. 4; supplementary table S11, Supplementary Material online), as expected given the stronger selective constraint on coding sites (Halligan and Keightley 2006). Moreover, *d_xy_* was slightly, but consistently lower for sympatric compared to allopatric comparisons of the species across all chromosome regions (Fig. 4; supplementary table S11, Supplementary Material online).

**Fig. 5.**
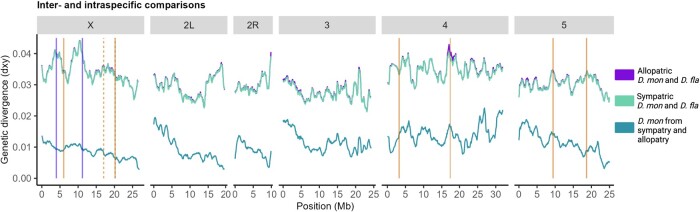
Genetic divergence (measured as *d*_*xy*_) across the genome (including intergenic regions) in sliding windows (window size 5,000 blocks, step size 500 blocks, and block length 64 bp) for allopatric and sympatric comparisons of *D. montana* and *D. flavomontana* (interspecific), and allopatric and sympatric *D. montana* populations (intraspecific). Vertical lines represent inversion breakpoints (supplementary fig. S5 and table S9, Supplementary Material online): dark purple solid lines and light orange solid and dashed lines indicate alternatively fixed inversions of *D. Montana* and *D. flavomontana*, respectively.

At noncoding sequences (i.e. intergenic and intronic), mean *d_xy_* was consistently higher in inverted compared to colinear regions in allopatric and sympatric comparisons (Fig. 4; supplementary table S11, Supplementary Material online). At coding sequences, mean *d_xy_* was increased for inversions on the fourth and the X chromosome compared to colinear regions both in allopatric and sympatric comparisons (Fig. 4; supplementary table S11, Supplementary Material online). Plotting *d_xy_* in sliding windows showed an increase in genetic divergence, especially around the inversion breakpoints and for overlapping X inversions for sympatric and allopatric comparisons of the species (Fig. 5; chromosomes shown individually in supplementary fig. S12, Supplementary Material online). A similar increase in *d_xy_* within *D. montana* (intraspecific comparison) was seen around some of the breakpoints on chromosomes 4 and 5, but not on chromosome X (Fig. 5). Based on a correlation analysis between inter and intraspecific *d_xy_*, chromosome 4 inversion appears to be an outlier in having a greater correlation than colinear regions (supplementary fig. S13, Supplementary Material online). This increased *d_xy_* both in interspecific and intraspecific crosses is potentially explained by a number of inversions that are polymorphic within *D. montana* on chromosome 4 (Stone et al. 1960; Throckmorton 1982).

*F*_ST_ was also generally higher for inverted compared to colinear regions, especially in allopatry, although these differences were nonsignificant (supplementary table S11, Supplementary Material online). The fact that the differences between inverted and colinear regions are less clear for *F*_ST_ than *d_xy_* reflects the susceptibility of *F*_ST_ to variation in genetic diversity (supplementary figs. S14 to S16, Supplementary Material online).

Overall, our finding of higher genetic divergence inside compared to outside of inversions is consistent with the idea that inversions suppress gene exchange and facilitate the accumulation/preservation of genetic differences (Fig. 4; supplementary table S11, Supplementary Material online; Navarro and Barton 2003; Kirkpatrick and Barton 2006). We also found that genetic divergence was highest around inversion breakpoints and in the series of overlapping inversions on the X (Fig. 5), where recombination is the most suppressed (Hoffmann and Rieseberg 2008). Similar signatures of elevated genetic divergence between closely related species inside and around inversion breakpoints have been detected, e.g. in other *Drosophila* species pairs (Noor et al. 2007; Kulathinal et al. 2009; Lohse et al. 2015), *Helianthus* sunflowers (Barb et al. 2014), *Sorex* shrews (Basset et al. 2006), and *Anopheles* mosquito (Michel et al. 2006). Finally, our finding of lower genetic divergence in sympatry compared to allopatry (Fig. 4; supplementary table S11, Supplementary Material online) is consistent with low levels of recent introgression in sympatry (Harrison and Larson 2014; Noor and Bennett 2009).

### No Evidence for Genes Being under Divergent Selection in Inversions

Alternatively fixed inversions may become hotspots for positively selected genetic differences, which can enhance adaptation and/or give rise to prezygotic and postzygotic barriers (Navarro and Barton 2003; Kirkpatrick and Barton 2006). To investigate whether genes under divergent selection are enriched within inversions, we performed a *d_N_*/*d_S_* analysis for *D. montana* and *D. flavomontana* using the branch-site model in codeML (supplementary file S2, Supplementary Material online).

We found 157 genes with evidence for divergent selection in *D. montana* and *D. flavomontana* (out of a total of 7,423 single-copy orthologs [SCOs]). Altogether, 45 positively selected genes were located inside inversions (1,997 SCOs within inversions altogether), but the inversions were not significantly enriched for genes under divergent selection (*G* = 0.159, *P* = 0.690). However, it is unlikely that we detected all genes under divergent selection since the statistical power of the approach may be relatively low for closely related species. While we find no signal of increased divergent selection in inversions in terms of the numbers of genes involved, the divergent genes inside inversions we identified include plausible targets for selection on potential barrier traits, such as chemoreception (odorant receptor 19a) (Hallem and Carlson 2006) and male fertility (testis-specific serine/threonine-protein kinase 3) (Nozawa et al. 2023). Moreover, even though none of the genes located near the inversion breakpoints were under divergent selection (supplementary files S1 and S2, Supplementary Material online), some of them may still be targets of selection as they have translocated alongside the inversions, and such translocations may give rise to new expression patterns (Villoutreix et al. 2021).

### Hierarchical Model Comparison Suggests Species Diverge with Very Low Levels of Postdivergence Gene Flow from *D. montana* to *D. flavomontana*

We used gIMble (Laetsch et al. 2023), an analytic likelihood method, to fit a series of demographic models of species divergence with and without long-term postdivergence gene flow, i.e. isolation with migration (IM) and strict divergence (DIV) models (supplementary fig. S17, Supplementary Material online), to the data summarized in terms of the blockwise site frequency spectrum (bSFS) (see Materials and Methods). The evolutionary history of the X chromosome (Charlesworth et al. 2018; Vicoso and Charlesworth 2006) and inversions (Lohse et al. 2015; Fuller et al. 2018) may differ from other chromosome regions, and these genomic partitions were therefore analyzed separately from colinear, autosomal regions. To minimize the direct effects of selection, our initial analysis was limited to intergenic sequences of the colinear autosomal regions (repetitive regions were excluded). We performed separate analyses for sympatric and allopatric comparisons of *D. montana* and *D. flavomontana*. To evaluate the timing of potential recent introgression in sympatry compared to allopatry, we also performed a separate analysis for intraspecific comparison of *D. montana* (*D. montana* living in contact vs. in isolation with *D. flavomontana*). We carried out this initial model comparison for the DIV and 2 IM models 4 times, using both *D. montana* and *D. flavomontana* as a reference genome to evaluate the potential effects of reference bias, and performed separate analyses for 2 different block lengths (64 and 128 bp). Parameter estimates and support values (lnCL) under all demographic models are shown in Table 1 for 64-bp blocks and using *D. montana* as a reference. Analogous analyses for all 4 combinations of block length and reference genomes are given in supplementary table S12, Supplementary Material online.

**Table 1:** Support (measured as ΔlnCL) and parameter estimates for divergence time (*T* in years/generations), migration rate (*m*), and effective population sizes (*N_e_*) for studied populations and their common ancestral population under strict DIV (*m* = 0) and IM models with both gene flow directions

Comparison	Model	*D. mon N_e_*	*D. fla N_e_*	Ancestral *N_e_*	*T*	*m*	lnCL	ΔlnCL
Allopatric	DIV	693,000	395,000	1,464,000	2,379,000	-	−45,651,205	12,869
	IM *D. mon –> D. fla*	705,000	382,000	1,403,000	2,539,000	1.09E^−08^	−45,638,336	0
	IM *D. fla –> D. mon*	692,000	396,000	1,457,000	2,398,000	1.21E^−09^	−45,650,952	12,616
Sympatric	DIV	720,000	392,000	1,459,000	2,343,000	-	−136,875,659	47,790
	IM *D. mon –> D. fla*	735,000	377,000	1,388,000	2,526,000	1.29E^−08^	−136,827,869	0
	IM *D. fla –> D. mon*	719,000	393,000	1,447,000	2,376,000	2.13E^−09^	−136,873,722	45,853

**Comparison**	**Model**	***D. mon allop N_e_***	***D. mon symp N_e_***	**Ancestral *N_e_***	***T***	***m***	**lnCL**	**ΔlnCL**

Intraspecific	DIV	1,087,000	1,560,000	858,000	210,000	-	−39,887,382	0
	IM *D. mon symp. –> D. mon allop.*	1,087,000	1,560,000	858,000	210,000	1.50E^−15^	−39,887,382	0
	IM *D. mon allop. –> D. mon symp.*	1,079,000	1,441,000	858,000	217,000	3.32E^−07^	−39,887,408	27

The model comparison is based on 64-bp blocks and the *D. montana* reference genome and was performed for intergenic autosomal colinear regions to minimize the effects of selection. Gray shading indicates the best-fit model for each comparison.

For both allopatric and sympatric comparisons and for 3 of the 4 combinations of block lengths and reference genomes used, the best-fitting demographic scenario was an IM model assuming introgression from *D. montana* into *D. flavomontana* (Table 1; supplementary table S12, Supplementary Material online). Our parametric bootstrap analyses showed that the improvement in fit of this IM model compared to the DIV model was significant suggesting a low but genuine signal of introgression (supplementary figs. S18 and S19, Supplementary Material online). The only exception was the analysis using shorter 64-bp blocks and *D. flavomontana* as a reference genome. In this case, the DIV model could not be rejected (supplementary fig. S19, Supplementary Material online). However, estimates for all parameters (*T* and *N_e_s*) were extremely similar regardless of the model (DIV and IM), block size (64 and 128 bp), and reference genome (*D. montana* and *D. flavomontana*) used (supplementary table S12, Supplementary Material online). Given the overall support for postdivergence gene flow and inherent bias of multilocus inference to underestimate migration, we assume an IM model with migration from *D. montana* into *D. flavomontana* as the best-fitting/most plausible scenario throughout all subsequent analyses (Table 1). Yusuf et al. (2022) also recently found signatures of introgression between *D. montana* and *D. flavomontana* using a different approach, which gives further support for our introgression signal.

In contrast, for the intraspecific comparison of *D. montana*, the DIV model could not be rejected in any analysis. When using 64-bp blocks, DIV and IM models had equal support, irrespective of which species was used as a reference (Table 1; supplementary table S12, Supplementary Material online). Analyses based on longer 128-bp blocks estimated slightly higher support for an IM model assuming postdivergence gene flow from allopatric (Alaskan) *D. montana* to sympatric (coastal/mountain) *D. montana* (Table 1; supplementary table S12, Supplementary Material online). However, the parametric bootstrap analyses showed that the improvement in fit compared to the simpler DIV model was nonsignificant (supplementary figs. S18 and S19, Supplementary Material online). Consequently, the subsequent intraspecific analyses were conducted using the DIV model (Table 1).

### Species-Specific Inversions Were Fixed Earlier or Around the Species’ Split, and Introgression Was Lower Inside Compared to Outside of Inversions and in Allopatry Compared to Sympatry

We used the best-fit IM model (Table 1) to examine the potential role of inversions and introgression in the speciation history of *D. montana* and *D. flavomontana*. As before, all analyses were limited to intergenic regions to minimize the effects of selection, and separate analyses were carried out for the X chromosome and autosomes, for inverted and colinear regions, and for sympatric and allopatric populations of the species. To estimate the timing of potential recent introgression, we analyzed the split time for *D. montana* living in contact (sympatry) or in isolation (allopatry) with *D. flavomontana* using the simpler strict DIV model (intraspecific comparison; Table 1).

**Fig. 6.**
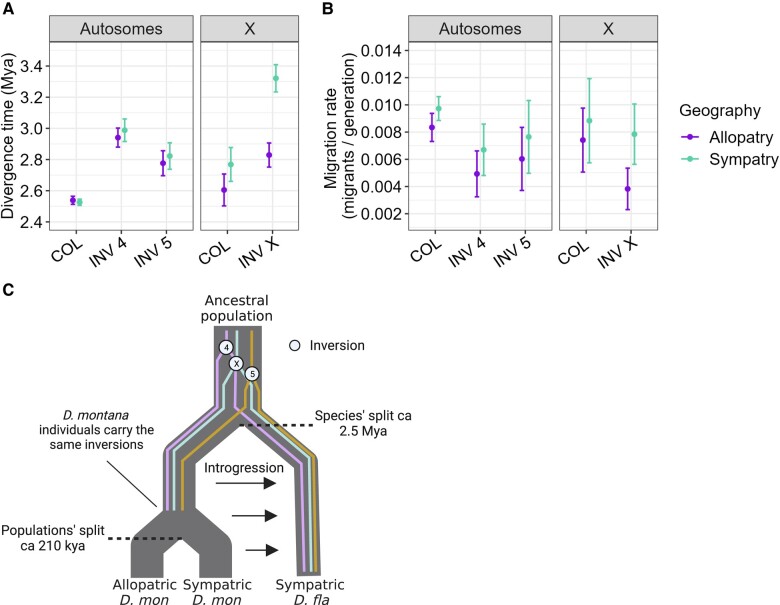
Estimates of A) split times and B) migration rates between *D. montana* and *D. flavomontana* for different chromosome partitions and for allopatric (dark purple) and sympatric (light green) comparisons. Confidence intervals were estimated using a parametric bootstrap as ±2 SD across 100 datasets simulated under the best-fit IM model with recombination (see Methods). C) Illustration of the likely evolutionary history of *D. montana* and *D. flavomontana*.

Taking the estimates for the colinear autosomal background as face value, *D. montana* and *D. flavomontana* have diverged ca. 2.5 Mya (Table 2; Fig. 6A and C). The divergence time estimates of the inversions differ from each other, and the inversions on the fourth, fifth, and the X chromosome predate the divergence time estimated for the colinear background (ca. 2.8 to 3.3 Mya) (Table 2; Fig. 6A and C). For all chromosome partitions, genetic diversity (*π*) and the effective population size (*N_e_*) of *D. montana* were approximately 2 times as large as those of *D. flavomontana* (Table 2; supplementary fig. S14, Supplementary Material online). *D. montana* populations living in contact (sympatry) and in isolation (allopatry) with *D. flavomontana* have diverged approximately 210,000 years ago (Table 2; Fig. 6C), an order of magnitude more recent than the split between *D. montana* and *D. flavomontana*.

**Table 2:** Parameters for effective populations sizes, divergence time (*t* in years/generations), and migration rate (*M*) estimated from 64-bp blocks under the IM*_mon_*_→ *fla*_ model for allopatric and sympatric comparisons and under the DIV model for intraspecific comparison

Comparison	Genomic region		*N_e_* ancestral	*N_e_ D. mon*	*N_e_ D. fla*	*T*	*M*
Allopatry	Autosomes	COL	1,403,000	705,000	382,000	2,539,000	0.0083
		4 INV	1,644,000	798,000	474,000	2,941,000	0.0049
		5 INV	1,364,000	718,000	396,000	2,777,000	0.0060
	X	COL	1,700,000	443,000	225,000	2,605,000	0.0074
		X INV	1,904,000	441,000	368,000	2,829,000	0.0038
Sympatry	Autosomes	COL	1,388,000	735,000	377,000	2,526,000	0.0097
		4 INV	1,603,000	865,000	470,000	2,988,000	0.0067
		5 INV	1,321,000	752,000	392,000	2,823,000	0.0076
	X	COL	1,592,000	493,000	219,000	2,769,000	0.0088
		X INV	1,591,000	607,000	365,000	3,321,000	0.0078

**Comparison**	**Genomic region**		***N_e_* ancestral**	***N_e_* allop *D. mon***	***N_e_* symp *D. mon***	***T***	

Intraspecific	Autosomes	COL	858,000	1,087,000	1,560,000	210,000	

Estimated long-term gene flow from *D. montana* to *D. flavomontana* was lower inside than outside of inversions both on the autosomes and the X (Table 2; Fig. 6B), which is in accordance with the finding that genetic divergence of noncoding (intergenic and intronic) sequences was consistently higher inside than outside of inversions (Fig. 4; supplementary table S11, Supplementary Material online). Moreover, migration rate estimates were higher in sympatry compared to allopatry (Table 2; Fig. 6B), which again agrees with the slightly, but consistently lower genetic divergence in sympatric compared to allopatric comparisons of the species (Fig. 4; supplementary table S11, Supplementary Material online).

Taken together, our analyses suggest that *D. montana* and *D. flavomontana* diverged ca. 2.5 Mya from a large ancestral population, which is broadly compatible with a recent estimate of 3.8 Mya based on small introns and the same molecular clock calibration (Yusuf et al. 2022). Crucially, split time estimates for all 5 fixed inversions we have identified on chromosomes X, 4, and 5 predate the estimated species split time based on the colinear background, which implies that these inversions must have existed already in the common ancestral population. In other words, we can rule out the possibility that the inversions arose after the onset of species divergence (in which case we would expect the divergence time estimates of inversions to overlap the estimated species divergence time). The reduced introgression for inversions compared to colinear regions we have estimated is a clear and expected consequence of reduced recombination and gene flow between alternative arrangements at each inversion.

What is less clear is the extent to which local adaptation in the face of gene flow in the ancestral population facilitated the fixation of these inversions (and vice versa) or whether the fixed inversions are a mere byproduct of population processes unrelated to speciation. The fact that 3 fixed inversions on the X are (i) overlapping and (ii) associated with a strong incompatibility preventing introgression from *D. montana* to *D. flavomontana* across the entire X chromosome (Poikela et al. 2023) suggests that at least the inversions on the X contributed to the buildup of reproductive isolation and acted as barriers to gene flow early on in the speciation process (Noor and Bennett 2009; Fuller et al. 2018). For example, these inversions may have been important in the initial ecological divergence of local populations of the ancestor, followed by the fast accumulation of genetic divergence and genetic incompatibilities. In contrast, we currently have no evidence that the inversions on chromosomes 4 and 5 are enriched for BDMIs (Poikela et al. 2023) or loci under divergent selection, so we cannot rule out a scenario in which these inversions have been maintained in the ancestral populations by balancing selection and have subsequently become fixed between *D. montana* and *D. flavomontana* simply by chance (Guerrero and Hahn 2017). In fact, even for the X inversions, we cannot verify whether the associated incompatibility allele(s) arose before or around the species’ split or afterward (postspeciation event). We stress that our comparison of divergence times estimated under the IM model between inverted and colinear parts of the genome relies on the assumption of neutrality (which is why we have restricted analyses of demographic history to intergenic sequence). If, however, some fraction of the intergenic partition is under selective constraint, we might expect higher genetic divergence within inversion: Berdan et al. (2021) recently showed using simulations that heterozygous inversions may accumulate nonadaptive, mildly deleterious mutations via less effective purifying selection within inversions, leading to higher genetic divergence even without any reduction in recombination between alternative arrangements.

Although we find evidence for postdivergence gene flow, it is worth highlighting that our estimate of the long-term rate of migration from *D. montana* to *D. flavomontana* is extremely small compared to analogous estimates for other young *Drosophila* sister species (Lohse et al. 2015); e.g. *D. mojavensis* and *D. arizonae* have approximately 1 migrant per generation, while our estimate for *D. montana* and *D. flavomontana* is roughly 1 migrant in 80 generations, 2 orders of magnitude lower. Thus, even the total probability of a lineage sampled in *D. flavomontana* to trace back to *D. montana* via migration (1−e^(−T M)^) is only 3.2%. This low rate of long-term effective migration agrees well with our previous evidence for strong prezygotic and postzygotic barriers between the species (Poikela et al. 2019). In addition, the species’ differences in the usage of host trees (Throckmorton 1982) and the ability to tolerate cold (Poikela et al. 2021) might have contributed to ecological isolation and reduced their encounters in nature. Intriguingly, we found higher levels of introgression in sympatry compared to allopatry, which suggests at least some introgression from *D. montana* to *D. flavomontana* over the past ∼210,000 years, i.e. after the allopatric (Alaskan) *D. montana* populations diverged from *D. montana* coexisting with *D. flavomontana*. Even low levels of introgression and selection against introgressed ancestry in the new genetic background may facilitate reinforcement of prezygotic barriers to prevent maladaptive hybridization between species and eventually complete the speciation process (Cruickshank and Hahn 2014; Servedio and Noor 2003). This is consistent with our previous finding that *D. flavomontana* has developed stronger prezygotic barriers against *D. montana* in sympatry compared to allopatry, presumably as a result of reinforcement (Poikela et al. 2019).

Our demographic inferences are limited in several ways. Firstly, the IM model is overly simplistic in assuming an instantaneous onset of species divergence and a constant rate of introgression throughout the species’ evolutionary history. However, given the overall extremely low estimate of gene flow and the computational limitations of gIMble, we have not attempted to fit—and therefore cannot exclude—more realistic (but necessarily more complex) demographic scenarios of either historical gene flow that reduced due to the emergence of strong barriers or sudden discrete bursts of admixture following periods of complete isolation. Secondly, our inference ignores recombination within blocks, a simplifying assumption that is known to lead to biased parameter estimates (Wall 2003). In particular, we found that the estimates of *T* obtained from parametric bootstrap replicates (simulated with recombination) are substantially larger (∼3.4 MY) than the true values (Table 2; Fig. 6; supplementary fig. S20, Supplementary Material online), which suggests that we have overestimated species divergence time overall. Finally, our approach of fitting an IM model to inverted regions ignores the fact that inversions arise in a single individual and may be fixed in a selective sweep. An inversion arising and sweeping to fixation immediately after the onset of species divergence would result in a lower estimate of *N_e_* for the species in which they fixed. If anything, we see the opposite pattern: i.e. larger estimates of *D. flavomontana N_e_* for the inversions on chromosomes 4 and 5 compared to the colinear background (Table 1), which is again compatible with an inversion origin in the ancestral population before the estimated species split. It is striking that all inversions date to a short interval just before the species split (∼600,000 years/generations) which is the same order as the (ancestral) population size. Given that we infer a substantially larger effective size for the ancestral population than for *D. montana* and *D. flavomontana*, one could interpret the interval in the ancestral population in which the inversions arose as the period of (rather than before) speciation.

Even though many species pairs differ from each other by multiple inversions, the majority of inversion differences must have arisen after speciation (Faria and Navarro 2010). Performing pairwise comparisons for younger and older species would offer a more holistic view of the role of inversions in speciation events. In our case, characterizing inversions and investigating divergence times and introgression across all species of the *montana* phylad of the *virilis* group (*D. montana*, *D. flavomontana*, *Drosophila borealis*, and *Drosophila lacicola*) (Hoikkala and Poikela 2022) could provide valuable additional information. In general, investigating millions of years old events by fitting necessarily drastically simplified scenarios of reality involves uncertainties.

## Conclusions

It has proven extremely difficult to test if and how inversions facilitate speciation, and empirical evidence on the role of inversions in speciation is largely lacking (Faria and Navarro 2010; Fuller et al. 2019). We explored these questions in 2 sister species of the *D. virilis* group, *D. montana* and *D. flavomontana*. Our main goals were (i) to characterize alternatively fixed chromosomal inversions of *D. montana* and *D. flavomontana*, (ii) to investigate the age of the inversions, and (iii) to identify whether the inversions have restricted gene exchange between *D. montana* and *D. flavomontana* during or before the onset of species divergence, which could have facilitated the accumulation or preservation of incompatibilities in the presence of gene flow.

Taking advantage of long- and short-read genome sequencing technologies, we generated the high-quality contiguous reference assemblies for *D. montana* and *D. flavomontana*. These genomes enabled us to accurately characterize inversions that are alternatively fixed between these sister species across their distribution area in North America. We were able to assign the majority of these to inversions that were previously described for the species based on polytene chromosome studies (Stone et al. 1960). Our analyses show that the inversions on chromosomes X, 4, and 5 arose before the onset of species divergence. Thus, the elevated genetic divergence within inversions results most likely from restricted recombination between alternative rearrangements, which were either under balancing selection or locally beneficial in different populations of the ancestral form. However, the X inversions have been found to contain strong BDMI that effectively restricts introgression from *D. montana* to *D. flavomontana* across the X chromosome in the first few backcross generations (Poikela et al. 2023) and provide evidence for the enrichment of BDMIs within inversions. Accordingly, our results are compatible with the idea that ancestrally polymorphic inversions, particularly the X chromosomal inversions in our case, can drive speciation potentially by facilitating initial ecological divergence and fast accumulation of genetic divergence and genetic incompatibilities (Fuller et al. 2018), while colinear regions keep exchanging genetic material until strong reproductive isolation has formed.

Even though the estimates of introgression between the species were extremely low, *D. flavomontana* has experienced some introgression from *D. montana* over the past ∼210,000 years in sympatric populations of the species. In general, selection can strengthen prezygotic barriers between species in response to low levels of poor functioning introgressed alleles, which likely leads to the strengthening of overall reproductive isolation and the completion of the speciation process (Cruickshank and Hahn 2014; Servedio and Noor 2003). This agrees with our previous evidence on *D. flavomontana* having developed stronger prezygotic barriers against *D. montana* in sympatric compared to allopatric populations of the species, potentially as a result of reinforcement (Poikela et al. 2019).

Overall, our results are compatible with the idea that inversions may be early triggers of the speciation process and highlight the value of interpreting the evolutionary effects of inversions through the lens of demographic models. However, in doing so, we have ignored much of the mechanistic and selective details of inversion evolution. Regions with repetitive sequences, such as transposable elements, tRNAs, ribosomal genes, or segmental duplications, are prone to breakage and are often the initial source of an inversion (Kapun and Flatt 2019). An in-depth investigation into the repetitive sequences or small structural variations around the inversion breakpoints would increase our understanding on how the inversions originated in the first place. Moreover, inversions are not static through their lifetime but evolve in response to changes in selection, genetic drift, new mutations, and gene flux (occurring via double cross-overs and gene conversion), as well as by interactions with other parts of the genome (Faria et al. 2018). Given the many, sometimes entangled processes affecting the origin and the fixation of inversions, models that can extract information about both demography and the selective forces acting on inversions in the early stages of speciation are the next obvious step in understanding how inversions facilitate the origin of species (Faria et al. 2018).

## Materials and Methods

### Sample Collections and Maintenance

*D. montana* and *D. flavomontana* females were collected from several sites in the Rocky Mountains and along the western coast of North America, and Alaska 2013 to 2015 (Fig. 1; supplementary table S1, Supplementary Material online). Sites in the Rocky Mountains and the western coast of North America are either inhabited by both species (sympatric sites: Jackson, Cranbrook, McBride, Terrace, Vancouver, Ashford, and Fall Creek) or by one of the species with nearby sites inhabited by both species (parapatric sites: Liberty, Afton, Livingston, and Azalea) (Fig. 1; supplementary table S1, Supplementary Material online). *D. montana* also has stable populations in high latitudes in Alaska, where *D. flavomontana* does not exist. We refer to the comparisons between *D. montana* and *D. flavomontana* from the Rocky Mountains and from the western coast as “sympatry” and those between *D. montana* from Alaska and *D. flavomontana* from the Rocky Mountains or the western coast as “allopatry” (Fig. 1). Intraspecific comparison was performed for *D. montana* living in isolation (allopatry) and in contact (sympatry) with *D. flavomontana* (Fig. 1).

The newly collected females were brought to the fly laboratory, with a constant light, 19 ± 1 °C and ∼60% humidity, at the University of Jyväskylä, Finland. Females that had mated with 1 or several males in nature were allowed to lay eggs in malt vials for several days, after which they were stored in 70% EtOH at −20 °C. The emerged F_1_ progeny of each female was kept together to produce the next generation and to establish isofemale strains. After that, also the F_1_ females were stored in 70% EtOH at −20 °C.

### DNA Extractions and Sequencing

We performed PacBio long-read sequencing from 2 *D. montana* and 2 *D. flavomontana* isofemale strains that had been kept in the fly laboratory since their establishment (Fig. 1; supplementary table S1, Supplementary Material online). DNA of the Seward *D. montana* and both *D. flavomontana* samples were extracted from a pool of 60 3-d-old females per isofemale strain using cetyltrimethylammonium bromide (CTAB) solution with RNAse treatment, phenol:chloroform:isoamyl alcohol (25:24:1) and chloroform:isoamyl alcohol (24:1) washing steps, and ethanol precipitation at the University of Jyväskylä, Finland. DNA of the Jackson *D. montana* sample was extracted with the “DNA Extraction SOP For Animal Tissue” protocol and purified with AMPure beads at BGI (Beijing Genomics Institute). Quality-checked DNA extractions of the Seward *D. montana* sample and both *D. flavomontana* samples were used to generate >15-kb PacBio libraries, which were all sequenced on 2 SMRT cells within a PacBio Sequel system (Pacific Biosciences, USA) at the Norwegian Sequencing Centre in 2018. DNA of the Jackson *D. montana* sample was used to generate >20-kb PacBio libraries and was sequenced on 1 SMRT cell using the PacBio Sequel system at BGI in 2019. Average PacBio raw read coverage was 27 to 35× per sample, except for the Jackson *D. montana* sample that was sequenced at 77× coverage. Detailed information on the PacBio raw reads of each sample is provided in supplementary table S2, Supplementary Material online.

We generated Illumina resequencing data for 12 *D. montana* and 9 *D. flavomontana* single wild-caught females or their F_1_ daughters from several locations in North America (Fig. 1; supplementary table S1, Supplementary Material online). DNA extractions were carried out at the University of Jyväskylä, Finland, using a CTAB method as described above. Quality-checked DNA extractions were used to produce an Illumina library for each sample in 3 batches. First, Nextera libraries were used to generate 150 bp paired-end (PE) reads on 2 lanes using HiSeq4000 Illumina instrument at Edinburgh Genomics in 2017. Second, 1 TruSeq library was used to generate 150-bp PE reads on one lane of a HiSeq4000 Illumina instrument at the Norwegian Sequencing Centre in 2018. Third, TruSeq libraries were used to generate 150-bp PE reads on 1 lane of a HiSeq X-Ten Illumina instrument at BGI in 2019. We generated on average 53 to 94× coverage per sample, except for the *D. montana* sample from Seward, which was sequenced to 435× coverage. Detailed information on Illumina raw reads is provided in supplementary table S2, Supplementary Material online.

### De Novo Genome Assemblies, Scaffolding, and Chromosome Synteny

We generated initial de novo assemblies for each PacBio data set and the respective Illumina reads using the wtdbg2 pipeline v2.5 (RedBean; Ruan and Li, 2020) and MaSuRCA hybrid assembler v3.3.9 (Zimin et al. 2017). To improve assembly contiguity, we used quickmerge for both assemblies of each sample (Chakraborty et al. 2016). The initial assembly statistics are given in supplementary table S13, Supplementary Material online. We polished the resulting assemblies with the respective Illumina reads using Pilon v1.23 (Walker et al. 2014) and removed uncollapsed heterozygous regions using purge_dups (Guan et al. 2020).

We identified genomic contaminants in the assemblies with BlobTools v1.1 (Laetsch and Blaxter 2017). PacBio and Illumina reads were first mapped back to each assembly with minimap2 (Li, 2018) and BWA mem (Burrows-Wheeler Aligner) v0.7.17 (Li and Durbin 2009), respectively. Contigs in the assemblies were then partitioned into taxonomic groups based on similarity search against the NCBI nucleotide database (BLASTn 2.9.0+; Camacho et al. 2009) and Uniref90 (Diamond v0.9.17; Buchfink et al. 2015). Finally, contigs were visualized on a scatter plot and colored by putative taxonomic groups (supplementary figs. S1 to S4, Supplementary Material online). Non-Diptera contigs were removed manually from the assemblies based on sequence GC content, read coverage, and taxonomy assignment. We estimated the completeness of the assemblies with the BUSCO pipeline v5.1.2 using the Diptera database “diptera_odb10” (Seppey et al. 2019), which searches for the presence of 3,285 conserved single-copy Diptera orthologs.

We constructed chromosome-level reference genomes for both species by scaffolding contigs of the original assemblies with a reference-guided scaffolding tool RagTag v2.1.0 (Alonge et al. 2019), which orients and orders the input contigs based on a reference using minimap2 (Li 2018). We used default settings except for the increased grouping confidence score (−i), which was increased to 0.6. For *D. montana*, we scaffolded the Seward *D. montana* assembly with the *D. lummei* genome, which was constructed using PacBio and Illumina reads and assigned to chromosomes using the published *D. virilis* chromosome map (Schäfer et al. 2010) and *D. virilis* assembly dvir_r1.03_FB2015_02 obtained from Flybase. For *D. flavomontana*, we first scaffolded the Livingston *D. flavomontana* assembly with the Vancouver *D. flavomontana* assembly and then with *D. lummei*. In *D. montana* and *D. flavomontana*, chromosome 2 has right (2R) and left (2L) arms, separated by a (sub)metacentric centromere, whereas in other *virilis* group species, the centromere is located near 1 end of the chromosome 2 (Stone et al. 1960). Therefore, scaffolding of chromosomes 2L and 2R was not feasible with the *D. lummei* genome.

For the *D. montana* chromosome-level reference genome, the X (29.1 Mb), 2L (20.2 Mb), and 2R (11.0 Mb) chromosomes could not be further scaffolded, while the lengths of chromosomes 3, 4, and 5 were increased substantially by scaffolding. The longest contig of chromosome 3 increased from 5.8 to 26.0 Mb (constructed from 37 contigs), chromosome 4 from 12.3 to 32.5 Mb (28 contigs), and chromosome 5 from 19.5 to 26.5 Mb (11 contigs; supplementary table S10, Supplementary Material online). For the *D. flavomontana* chromosome-level reference genome, the X chromosome (29.0 Mb) could not be further scaffolded, while the lengths of all other chromosomes increased due to scaffolding. Chromosome 2L increased from 10.2 to 20.4 Mb in length (3 contigs), 2R from 10.4 to 10.6 Mb (3 contigs), the third chromosome from 7.8 to 24.5 Mb (33 contigs), the fourth chromosome from 20.0 to 30.7 Mb (14 contigs), and the fifth chromosome from 23.5 to 27.2 Mb (4 contigs; supplementary table S10, Supplementary Material online).

Finally, we investigated chromosome synteny between species of the *montana* phylad (*D. montana* and *D. flavomontana*; monSE13F37 and flaMT13F11 assemblies) and *virilis* phylad (*D. virilis* and *D. lummei*) (Yusuf et al. 2022) using minimap2synteny.py (Mackintosh et al. 2023). Prior to using minimap2synteny.py, we aligned species’ assemblies using minimap2 v.2.17 (Li 2018) with the option -x asm10 and kept alignments with a mapping quality of 60.

### Genome Annotations

All genome assemblies were annotated for repetitive regions and genes. De novo libraries of repeat sequences were built for each assembly using RepeatModeler v2.0.1 (Flynn et al. 2019), and repetitive regions were softmasked, together with *Drosophila*-specific repeats, Dfam_3.1 (Hubley et al. 2016) and RepBase-20181026 (Bao et al. 2015), using RepeatMasker v4.1.0 (Smit et al. 2013-2015). Gene models were predicted on the softmasked assemblies of *D. montana* using the BRAKER2 pipeline. For gene annotation, we used RNA-seq data (Illumina TruSeq 150-bp PE) from whole-body female and male *D. montana* adult flies collected in Finland (Parker et al. 2021). RNA-seq reads were trimmed for adapter contamination and read quality using fastp v0.20.0 (Chen et al. 2018) and mapped to both softmasked *D. montana* assemblies using STAR v2.7.0 (Dobin et al. 2013). Finally, *D. montana* gene annotations were carried out with BRAKER2s ab initio gene prediction pipeline with RNA-seq evidence using Augustus v3.3.3 and GeneMark-ET v4.48 (Hoff et al. 2019, 2016; Li et al. 2009; Barnett et al. 2011; Lomsadze et al. 2014; Stanke et al. 2006, 2008). Protein predictions of the Jackson *D. montana* assembly with the best BUSCO values (see the Results and Discussion) were used to annotate both *D. flavomontana* and both chromosome-level genomes using the BRAKER2s ab initio gene prediction pipeline with GenomeThreader and AUGUSTUS (Stanke et al. 2006, 2008; Gremme 2012; Buchfink et al. 2015; Hoff et al. 2016, 2019). Annotation completeness was assessed using BUSCO v5.1.2 against the “diptera_odb10” database (Seppey et al. 2019).

### Mapping, Variant Calling, and Variant Filtering

To investigate genome-wide variation in sympatric and allopatric populations of the species, we mapped all Illumina samples to the *D. montana* chromosome-level assembly. For this, we quality-checked Illumina PE reads of each sample with FastQC v0.11.8 (Andrews 2010) and trimmed them for adapter contamination and low-quality bases using fastp v0.20.0 (Chen et al. 2018). We mapped each trimmed Illumina sample against the genome using BWA mem v0.7.17 with read group information (Li and Durbin 2009), sorted alignments with SAMtools v1.10 (Li et al. 2009), and marked PCR duplicates with sambamba v0.7.0 (Tarasov et al. 2015). The resulting BAM files were used for variant calling with freebayes v1.3.1-dirty (Garrison and Marth 2012). Raw variants were processed with gIMble preprocess (genome-wide IM blockwise likelihood estimation toolkit; Laetsch et al. 2023). In brief, non-SNP variants were deconstructed into allelic primitives, where remaining non-SNPs were removed in addition to any SNP variant within 2 bases of a non-SNP. Genotype calls of remaining SNP variants were set to missing if any of the following assumptions was violated: (i) sample depth (FMT/DP) between 8 and 2 SD from the mean coverage, (ii) read directionality placement balance (RPL ≥ 1, RPR ≥ 1), or (iii) read strand placement balance (SAF ≥ 1, SAR ≥ 1).

### PCA of SNP and Climatic Data

To group Illumina samples according to their species and population type, we performed a PCA on the filtered VCF file, including all samples, chromosomes, and coding, intronic, and intergenic SNPs using PLINK v1.9 package (Chang et al. 2015). The VCF file was converted to PLINK's BED/BIM format, and the PCA was run with PLINK's --pca function.

We performed another PCA on the climatic variables at fly sampling sites to visualize the climatic variation among them. First, we downloaded climatic information from WorldClim database v2.1 (2.5-min spatial resolution, data set 1970 to 2000; Fick and Hijmans 2017) using latitudinal and longitudinal coordinates of each site (supplementary table S1, Supplementary Material online) and extracted 19 bioclimatic variables using the “raster” package v2.8-19 (Hijmans 2020; supplementary tables S5 and S6, Supplementary Material online). We then performed PCA on the bioclimatic variables, describing temperature and humidity conditions in each site. We performed the PCA using the “FactoMineR” package (Lê et al. 2008) in R v4.3.1 and R studio v2023.03.0.

### Characterization of Chromosomal Inversions

We identified large (>500 kb) alternatively fixed inversions between *D. montana* and *D. flavomontana* using long- and short-read data as well as genome assemblies. We mapped PacBio reads of each sample against each of the 4 assemblies using ngmlr v0.2.7 (Sedlazeck et al. 2018) and obtained structural variant (SV) candidates from the SV identification software, Sniffles v1.0.12 (Sedlazeck et al. 2018). We also mapped Illumina PE reads against each of the 4 assemblies as explained in the “Mapping, Variant Calling, and Variant Filtering” paragraph. The resulting BAM files were given to Delly v0.8.1, which identifies SVs based on PE read orientation and split-read evidence (Rausch et al. 2012). We used SURVIVOR (Jeffares et al. 2017) to identify species-specific, geographically widespread inversions that were shared by Sniffles and Delly outputs and that were found in at least in 9 *D. montana* (out of 12) and 6 *D. flavomontana* (out of 9) samples. Putative breakpoints of each inversion were located within a single contig, except for the fourth chromosome inversion where breakpoints were located in 2 different contigs (supplementary table S9, Supplementary Material online). This inversion was therefore verified by mapping long- and short-read data against the *D. lummei* genome that has a more contiguous chromosome 4 (supplementary table S9 and fig. S10, Supplementary Material online). To determine whether the inversions belong to *D. montana* or *D. flavomontana*, we mapped PacBio reads of *D. lummei* (acting as an outgroup) against *D. montana* and *D. flavomontana* assemblies and investigated SVs using Sniffles. The putative breakpoints of the inversions were confirmed visually with Integrative Genomics Viewer (IGV) v2.8.0 (Thorvaldsdóttir et al. 2013) using both long- and short-read data (an example IGV view shown in supplementary fig. S21, Supplementary Material online).

Alternatively fixed inversions were also illustrated by aligning assemblies of *D. montana*, *D. flavomontana*, *D. virilis*, and *D. lummei* using minimap2synteny.py (as explained in the paragraph “De Novo Genome Assemblies, Scaffolding, and Chromosome Synteny”; Fig. 2) and nucmer alignments of the MUMmer package (Marçais et al. 2018) together with Dot plots (https://dot.sandbox.bio/; supplementary figs. S6 to S10, Supplementary Material online).

Inversion breakpoints are typically named proximal and distal based on their distance from the centromere. Since there is no prior knowledge of *D. montana* and *D. flavomontana* centromeres, we identified their approximate location based on *D. virilis* chromosome maps (chromosome 2L) and genes (X: *yellow*, 4: *bl*, 5: *Cid5* and *l(2)not*) located near centromeres or telomeres (Schaeffer et al. 2008; Kursel and Malik 2017). The number of PacBio reads supporting each breakpoint and genes and repetitive sequences located within the 5-kb region of the breakpoints (2.5-kb flanking each side of the breakpoints) are given in supplementary file S1, Supplementary Material online.

### Modeling Divergence and Postdivergence Gene Flow

We analyzed mean genetic divergence (*d_xy_*) and differentiation (*F_st_*) and fitted models of species divergence with and without long-term interspecific gene flow between and within the species using gIMble (Laetsch et al. 2023). This analytic likelihood method uses the joint distribution of mutation types in short sequence blocks, the bSFS, across subsamples of pairs of individual genomes to fit a series of models of speciation history. We summarized data by the bSFS for 2 block lengths, 64 and 128 bp.

Given the potentially different evolutionary history of the X chromosome and the autosomes (Charlesworth et al. 2018; Vicoso and Charlesworth 2006), we ran separate analyses for them throughout. Colinear regions, ending at inversion breakpoints, were combined across autosomes as these regions are expected to share the same evolutionary history, while inversions from different chromosomes may differ in age and were analyzed separately. The overlapping inversions of the X chromosome were analyzed together following Counterman and Noor (2006) and Lohse et al. (2015). We analyzed different chromosome partitions separately for allopatric and sympatric comparisons of the species. We also analyzed the split time of *D. montana* populations living in isolation (allopatry) and in contact (sympatry) with *D. flavomontana* to evaluate the timing of potential recent introgression between the 2 species. The intraspecific divergence time was inferred from the colinear autosomal regions, i.e. the same data partition we used to infer the interspecific background history.

We first calculated *d_xy_* and *F*_ST_ for different genomic regions (i.e. colinear and inverted autosomes and colinear and inverted X chromosome) and for allopatric and sympatric populations to evaluate the role of inversions in suppressing gene exchange. These analyses were carried out separately for coding, intronic, and intergenic regions (repetitive regions were excluded from all data partitions). To test whether *d_xy_* and *F*_ST_ were increased within inversions, we simulated data sets corresponding in size to the data sampled for each inversion under the background demography (inferred from colinear autosomal regions) and compared the observed *d_xy_* and *F*_ST_ to the distributions. We simulated inversion data sets under a minimal, conservative model of recombination, which allows for gene conversion but no cross-over. We assumed a rate of (initiation of) gene conversion of 3.59 × 10^–8^ per base per generation. This corresponds to recent estimates for *Drosophila pseudoobscura* and *Drosophila persimilis* (1.4 × 10^–5^ converted sites per base per generation; mean GC tract length of 390 bp) (Korunes and Noor 2018). We simulated sequences of 100 kb in length, 2 orders of magnitude shorter than total length of intergenic sequence per inversion.

Before analyzing different chromosome partitions, we investigated the likely evolutionary history of *D. montana* and *D. flavomontana* by comparing the likelihood of different demographic models. We limited this initial model selection of allopatric, sympatric, and intraspecific comparisons to intergenic sequences of colinear autosomal regions (repetitive regions excluded) to minimize the effects of selection. The simplest, strict DIV model considers isolation at time *T* without interspecific gene flow, i.e. isolation in allopatry (supplementary fig. S17A, Supplementary Material online). The IM model allows unidirectional migration rate at a constant rate *M* (supplementary fig. S17B, Supplementary Material online). The IM model was fitted to both gene flow directions (i.e. from *D. montana* to *D. flavomontana* and from *D. flavomontana* to *D. montana* and from allopatric to sympatric *D. montana* and from sympatric to allopatric *D. montana*). The DIV and IM models allow asymmetric effective population size (*N_e_*) between the descendent populations and a separate *N_e_* for the ancestral population. Analyses based on the bSFS assume a constant mutation rate (*μ*) across blocks and no recombination within them. We assumed a mutation rate (*μ*) of 2.8 × 10^−9^ per site per generation, based on an estimate of the spontaneous mutation rate in *D. melanogaster* (Keightley et al. 2014). The estimates of *T* are converted into absolute time using *t* = *T* × 2*N_e_* × *g*, where *N_e_* = *θ*/(4*μ*) and *g* is generation time. We assumed 1 generation per year, i.e. the generation time of Alaskan *D. montana* populations and most likely that of the ancestral population of the species, even though other *D. montana* and *D. flavomontana* populations presently have 2 generations per year (Tyukmaeva et al. 2020). To consider the potential effects of reference bias, we performed model fitting and selection twice using both *D. montana* and *D. flavomontana* chromosome-level assemblies as reference genomes.

To estimate the uncertainty in parameter estimates, i.e. the difference in support (ΔlnCL) between different demographic scenarios, we performed a parametric bootstrap. We used gIMble simulation to simulate 100 replicate data sets (of the same size as the real data in terms of the numbers of blocks). To include the effect of linkage between blocks, we simulated data in 1,000 chunks assuming a recombination rate of 8.9 × 10^−9^ calculated from the total map length (i.e. 1.76 × 10^−8^ divided by 2 given the absence of recombination in males). Specifically, we simulated data under the DIV model and fitted that model to the DIV and the best-fitting IM model to each replicate to obtain a null distribution of ΔlnCL between models (see supplementary figs. S18 and S19, Supplementary Material online).

Finally, to investigate the role of inversions in speciation, we performed demographic analyses under the best-fit model separately for different chromosome partitions (i.e. colinear and inverted autosomes and colinear and inverted X chromosome) and for allopatric and sympatric comparisons of *D. montana* and *D. flavomontana*. The uncertainties in estimates of *T* and *M* for each data partition were inferred from 100 parametric bootstrap replicates/simulations.

### Genes Putatively under Divergent Selection

To identify genes putatively under positive selection between *D. montana* and *D. flavomontana*, wild-caught Illumina females (supplementary table S1, Supplementary Material online) from sympatric populations were assembled with MaSuRCA v3.3.9 (Zimin et al. 2017). Furthermore, *Drosophila littoralis* female (strain ID KL13F60), collected from Korpilahti, Finland (62°00′N; 25°34′E) in 2013 and sequenced at BGI in 2019 (details in the “DNA Extractions and Sequencing” paragraph), was assembled and used as an outgroup in the *d_N_*/*d_S_* analysis. The completeness of the assemblies was assessed using BUSCO v5.1.2 with diptera_odb10 database (Seppey et al. 2019). The genomes were annotated using protein predictions of Jackson *D. montana* PacBio assembly with the best BUSCO values (see supplementary table S3, Supplementary Material online) using BRAKER2s ab initio gene prediction pipeline with GenomeThreader and AUGUSTUS (Stanke et al. 2006, 2008; Gremme 2012; Buchfink et al. 2015; Hoff et al. 2016, 2019).

For the *d_N_*/*d_S_* analysis, we chose samples, which have originated from climatically variable populations (Fig. 3B) and obtained >97% single-copy BUSCOs (supplementary table S14, Supplementary Material online). The high BUSCO values, as a proxy of high genome quality, result in a higher number of genes to be included in the analysis. Accordingly, we used *D. montana* samples from Terrace, Fall Creek, Azalea, and Cranbrook and *D. flavomontana* from Terrace, Fall Creek, McBride, and Cranbrook. SCOs between the samples were first identified with OrthoFinder (v2.5.4) (Emms and Kelly 2019). The rooted phylogenetic tree produced by OrthoFinder showed clear groupings of *D. montana*, *D. flavomontana*, and the outgroup (supplementary fig. S22, Supplementary Material online).

The SCO proteins were aligned using Prank v.170427 and the corresponding genes codon aligned with pal2nal v14.1. To identify genes under positive selection, we evaluated the rate of nonsynonymous (*d_N_*) to synonymous (*d_S_*) substitutions (*d_N_*/*d_S_*), also known as omega (*ω*), across the orthologs. We used GWideCodeML (Macías et al. 2020) to run CodeML (Yang 1997) with branch-site models for all orthologs. The tree from OrthoFinder was unrooted using Retree (Felsenstein 1989) and used as input for GWideCodeML. Two models were defined: the null model H_0_ (parameters model = 2, NSites = 2, fix_omega = 1, and omega = 1) that assumes no positive selection, and the alternative model H_A_ that shares the other settings of H_0_ but does not fix *ω* (omega = 0), allowing for optimization of this parameter. Both species were tested as being under selection. The built-in likelihood ratio tests of GWideCodeML were used to examine the orthologs, with a significantly better fit of the H_A_ model indicating the presence of positive selection.

The positively selected genes were mapped to the *D. montana* chromosome-level reference genome by extracting a representative protein sequence for each orthogroup from 1 randomly selected sample (flaCRAN14F7) and blasting it against the *D. montana* chromosome-level reference proteome using Diamond v2.0.15 (Buchfink et al. 2015). We blasted the genes under selection against *D. virilis* RefSeq proteins using BLASTp v2.9.0+ (Camacho et al. 2009) to obtain functional predictions for the orthologs. RefSeq protein IDs and functional predictions for the SCOs and genes putatively under divergent selection are given in supplementary file S2, Supplementary Material online. Finally, we performed a *G*-test to explore whether genes under divergent selection are enriched inside inversions.

## Supplementary Material

evae024_Supplementary_Data

## Data Availability

Raw sequencing reads are available at SRA and genome assemblies at GenBank under BioProject PRJNA939085. Scaffolded chromosome-level genome assemblies are available at 10.5281/zenodo.10635471. Unix and R commands and Jupyter Notebooks used in the study are available in https://github.com/noorlinnea.

## References

[evae024-B1] Alonge M , Soyk S, Ramakrishnan S, Wang X, Goodwin S, Sedlazeck FJ, Lippman ZB, Schatz MC. RaGOO: fast and accurate reference-guided scaffolding of draft genomes. Genome Biol. 2019:20(1):1–17. 10.1186/s13059-019-1829-6. 31661016 PMC6816165

[evae024-B2] Andrews S . FastQC: A quality control tool for high throughput sequence data. 2010. http://www.bioinformatics.babraham.ac.uk/projects/fastqc/.

[evae024-B3] Bao W , Kojima KK, Kohany O. Repbase update, a database of repetitive elements in eukaryotic genomes. Mob DNA. 2015:6(1):4–9. 10.1186/s13100-015-0041-9. 26045719 PMC4455052

[evae024-B4] Barb JG , Bowers JE, Renaut S, Rey JI, Knapp SJ, Rieseberg LH, Burke JM. Chromosomal evolution and patterns of introgression in *Helianthus*. Genetics. 2014:197(3):969–979. 10.1534/genetics.114.165548. 24770331 PMC4096374

[evae024-B5] Barnett DW , Garrison EK, Quinlan AR, Str̈mberg MP, Marth GT. Bamtools: a C++ API and toolkit for analyzing and managing BAM files. Bioinformatics. 2011:27(12):1691–1692. 10.1093/bioinformatics/btr174. 21493652 PMC3106182

[evae024-B6] Basset P , Yannic G, Brünner H, Hausser J. Restricted gene flow at specific parts of the shrew genome in chromosomal hybrid zones. Evolution (N Y). 2006:60:1718–1730. 10.1111/j.0014-3820.2006.tb00515.x. 17017071

[evae024-B7] Berdan EL , Blanckaert A, Butlin RK, Bank C. Deleterious mutation accumulation and the long-term fate of chromosomal inversions. PLoS Genet. 2021:17(3):e1009411. 10.1371/journal.pgen.1009411. 33661924 PMC7963061

[evae024-B8] Buchfink B , Xie C, Huson DH. Fast and sensitive protein alignment using DIAMOND. Nat Methods. 2015:12(1):59–60. 10.1038/nmeth.3176. 25402007

[evae024-B9] Butlin RK . Recombination and speciation. Mol Ecol. 2005:14(9):2621–2635. 10.1111/j.1365-294X.2005.02617.x. 16029465

[evae024-B10] Camacho C , Coulouris G, Avagyan V, Ma N, Papadopoulos J, Bealer K, Madden TL. BLAST+: architecture and applications. BMC Bioinformatics. 2009:10(1):421. 10.1186/1471-2105-10-421. 20003500 PMC2803857

[evae024-B11] Chakraborty M , Baldwin-Brown JG, Long AD, Emerson JJ. Contiguous and accurate de novo assembly of metazoan genomes with modest long read coverage. Nucleic Acids Res. 2016:44(19):e147. 10.1093/nar/gkw654. 27458204 PMC5100563

[evae024-B12] Chang CC , Chow CC, Tellier LC, Vattikuti S, Purcell SM, Lee JJ. Second-generation PLINK: rising to the challenge of larger and richer datasets. Gigascience. 2015:4:7. 10.1186/s13742-015-0047-8. 25722852 PMC4342193

[evae024-B13] Charlesworth B . Measures of divergence between populations and the effect of forces that reduce variability. Mol Biol Evol. 1998:15(5):538–543. 10.1093/oxfordjournals.molbev.a025953. 9580982

[evae024-B14] Charlesworth B , Campos JL, Jackson BC. Faster-X evolution: theory and evidence from *Drosophila*. Mol Ecol. 2018:27(19):3753–3771. 10.1111/mec.14534. 29431881

[evae024-B15] Charlesworth B , Coyne JA, Barton NH. The relative rates of evolution of sex chromosomes and autosomes. Am Nat. 1987:130(1):113–146. 10.1086/284701.

[evae024-B16] Chen S , Zhou Y, Chen Y, Gu J. Fastp: an ultra-fast all-in-one FASTQ preprocessor. Bioinformatics. 2018:34(17):i884–i890. 10.1093/bioinformatics/bty560. 30423086 PMC6129281

[evae024-B17] Connallon T , Olito C, Dutoit L, Papoli H, Ruzicka F, Yong L. Local adaptation and the evolution of inversions on sex chromosomes and autosomes. Philos Trans R Soc B Biol Sci. 2018:373(1757):20170423. 10.1098/rstb.2017.0423. PMC6125732 30150221

[evae024-B18] Counterman BA , Noor MAF. Multilocus test for introgression between the cactophilic species *Drosophila mojavensis* and *Drosophila arizonae*. Am Nat. 2006:168(5):682–696. 10.1086/508632. 17080365

[evae024-B19] Cridland JM , Macdonald SJ, Long AD, Thornton KR. Abundance and distribution of transposable elements in two *Drosophila* QTL mapping resources. Mol Biol Evol. 2013:30(10):2311–2327. 10.1093/molbev/mst129. 23883524 PMC3773372

[evae024-B20] Cruickshank TE , Hahn MW. Reanalysis suggests that genomic islands of speciation are due to reduced diversity, not reduced gene flow. Mol Ecol. 2014:23(13):3133–3157. 10.1111/mec.12796. 24845075

[evae024-B21] Dagilis AJ , Kirkpatrick M. Prezygotic isolation, mating preferences, and the evolution of chromosomal inversions. Evolution. 2016:70(7):1465–1472. 10.1111/evo.12954. 27174252

[evae024-B22] Dobin A , Davis CA, Schlesinger F, Drenkow J, Zaleski C, Jha S, Batut P, Chaisson M, Gingeras TR. STAR: ultrafast universal RNA-seq aligner. Bioinformatics. 2013:29(1):15–21. 10.1093/bioinformatics/bts635. 23104886 PMC3530905

[evae024-B23] Emms DM , Kelly S. OrthoFinder: phylogenetic orthology inference for comparative genomics. Genome Biol. 2019:20(1):1–14. 10.1186/s13059-019-1832-y. 31727128 PMC6857279

[evae024-B24] Fang Z , Pyhäjärvi T, Weber AL, Dawe RK, Glaubitz JC, González JdJS, Ross-Ibarra C, Doebley J, Morrell PL, Ross-Ibarra J, et al Megabase-scale inversion polymorphism in the wild ancestor of maize. Genetics. 2012:191(3):883–894. 10.1534/genetics.112.138578. 22542971 PMC3389981

[evae024-B25] Faria R , Johannesson K, Butlin RK, Westram AM. Evolving inversions. Trends Ecol Evol. 2018:34:239–248. 10.1016/j.tree.2018.12.005. 30691998

[evae024-B26] Faria R , Navarro A. Chromosomal speciation revisited: rearranging theory with pieces of evidence. Trends Ecol Evol. 2010:25(11):660–669. 10.1016/j.tree.2010.07.008. 20817305

[evae024-B27] Felsenstein J . PHYLIP: phylogeny inference package. Version 3.2. Cladistics. 1989:5:164–166. 10.1086/416571.

[evae024-B28] Fick SE , Hijmans RJ. WorldClim 2: new 1-km spatial resolution climate surfaces for global land areas. Int J Climatol. 2017:37(12):4302–4315. 10.1002/joc.5086.

[evae024-B29] Fishman L , Stathos A, Beardsley PM, Williams CF, Hill JP. Chromosomal rearrangements and the genetics of reproductive barriers in *Mimulus* (monkey flowers). Evolution (N Y). 2013:67:2547–2560. 10.1111/evo.12154. 24033166

[evae024-B30] Flynn JM , Hubley R, Goubert C, Rosen J, Clark AG, Feschotte C, Smit AF. RepeatModeler2: Automated genomic discovery of transposable element families. bioRxiv. 2019. 10.1101/856591. PMC7196820 32300014

[evae024-B31] Fuller ZL , Koury SA, Phadnis N, Schaeffer SW. How chromosomal rearrangements shape adaptation and speciation: case studies in *Drosophila pseudoobscura* and its sibling species *Drosophila persimilis*. Mol Ecol. 2019:28(6):1283–1301. 10.1111/mec.14923. 30402909 PMC6475473

[evae024-B32] Fuller ZL , Leonard CJ, Young RE, Schaeffer SW, Phadnis N. Ancestral polymorphisms explain the role of chromosomal inversions in speciation. PLoS Genet. 2018:14(7):e1007526. 10.1371/journal.pgen.1007526. 30059505 PMC6085072

[evae024-B33] Garrison E , Marth G. Haplotype-based variant detection from short-read sequencing. arXiv preprint arXiv: 1207.3907. 2012. http://arxiv.org/abs/1207.3907.

[evae024-B34] Gremme G . Computational gene structure prediction (Doctoral dissertation, Staats-und Universitätsbibliothek Hamburg Carl von Ossietzky). 2012.

[evae024-B35] Guan D , McCarthy SA, Wood J, Howe K, Wang Y, Durbin R. Identifying and removing haplotypic duplication in primary genome assemblies. Bioinformatics. 2020:36(9):2896–2898. 10.1093/bioinformatics/btaa025. 31971576 PMC7203741

[evae024-B36] Guerrero RF , Hahn MW. Speciation as a sieve for ancestral polymorphism. Mol Ecol. 2017:26(20):5362–5368. 10.1111/mec.14290. 28792649

[evae024-B37] Hallem EA , Carlson JR. Coding of odors by a receptor repertoire. Cell. 2006:125(1):143–160. 10.1016/j.cell.2006.01.050. 16615896

[evae024-B38] Halligan DL , Keightley PD. Ubiquitous selective constraints in the *Drosophila* genome revealed by a genome-wide interspecies comparison. Genome Res. 2006:16(7):875–884. 10.1101/gr.5022906. 16751341 PMC1484454

[evae024-B39] Harrison RG , Larson EL. Hybridization, introgression, and the nature of species boundaries. J Hered. 2014:105(S1):795–809. 10.1093/jhered/esu033. 25149255

[evae024-B40] Hijmans RJ . raster: Geographic data analysis and modeling. R package version 2.8-19. 2020. http://CRAN.R-project.org/package=raster.

[evae024-B41] Hoff KJ , Lange S, Lomsadze A, Borodovsky M, Stanke M. BRAKER1: unsupervised RNA-seq-based genome annotation with GeneMark-ET and AUGUSTUS. Bioinformatics. 2016:32(5):767–769. 10.1093/bioinformatics/btv661. 26559507 PMC6078167

[evae024-B42] Hoff KJ , Lomsadze A, Borodovsky M, Stanke M. Whole-genome annotation with BRAKER. Methods Mol Biol. 2019:1962:65–95. 10.1007/978-1-4939-9173-0_5. 31020555 PMC6635606

[evae024-B43] Hoffmann AA , Rieseberg LH. Revisiting the impact of inversions in evolution: from population genetic markers to drivers of adaptive shifts and speciation? Annu Rev Ecol Evol Syst. 2008:39(1):21–42. 10.1146/annurev.ecolsys.39.110707.173532. 20419035 PMC2858385

[evae024-B44] Hoikkala A , Poikela N. Adaptation and ecological speciation in seasonally varying environments at high latitudes: *Drosophila virilis* group. Fly (Austin). 2022:16(1):85–104. 10.1080/19336934.2021.2016327. 35060806 PMC8786326

[evae024-B45] Hubley R , Finn RD, Clements J, Eddy SR, Jones TA, Bao W, Smit AFA, Wheeler TJ. The Dfam database of repetitive DNA families. Nucleic Acids Res. 2016:44(D1):D81–D89. 10.1093/nar/gkv1272. 26612867 PMC4702899

[evae024-B46] Jackson BC . Recombination-suppression: how many mechanisms for chromosomal speciation? Genetica. 2011:139(3):393–402. 10.1007/s10709-011-9558-0. 21327492

[evae024-B47] Jeffares DC , Jolly C, Hoti M, Speed D, Shaw L, Rallis C, Balloux F, Dessimoz C, Bähler J, Sedlazeck FJ, et al Transient structural variations have strong effects on quantitative traits and reproductive isolation in fission yeast. Nat Commun. 2017:8(1):14061. 10.1038/ncomms14061. 28117401 PMC5286201

[evae024-B48] Kapun M , Flatt T. The adaptive significance of chromosomal inversion polymorphisms in *Drosophila melanogaster*. Mol Ecol. 2019:28(6):1263–1282. 10.1111/mec.14871. 30230076

[evae024-B49] Kapun M , Schmidt C, Durmaz E, Schmidt PS, Flatt T. Parallel effects of the inversion In(3R)Payne on body size across the North American and Australian clines in *Drosophila melanogaster*. J Evol Biol. 2016:29(5):1059–1072. 10.5061/dryad.8ns67. 26881839 PMC4867298

[evae024-B50] Keightley PD , Ness RW, Halligan DL, Haddrill PR. Estimation of the spontaneous mutation rate per nucleotide site in a *Drosophila melanogaster* full-sib family. Genetics. 2014:196(1):313–320. 10.1534/genetics.113.158758. 24214343 PMC3872194

[evae024-B51] Kirkpatrick M , Barton N. Chromosome inversions, local adaptation and speciation. Genetics. 2006:173(1):419–434. 10.1534/genetics.105.047985. 16204214 PMC1461441

[evae024-B52] Korunes KL , Noor MAF. Pervasive gene conversion in chromosomal inversion heterozygotes. Mol Ecol. 2018:28(6):1302–1315. 10.1111/mec.14921. 30387889 PMC6475484

[evae024-B53] Kulathinal RJ , Stevison LS, Noor MAF. The genomics of speciation in *Drosophila*: diversity, divergence, and introgression estimated using low-coverage genome sequencing. PLoS Genet. 2009:5(7):e1000550. 10.1371/journal.pgen.1000550. 19578407 PMC2696600

[evae024-B54] Kursel LE , Malik HS. Recurrent gene duplication leads to diverse repertoires of centromeric histones in *Drosophila* species. Mol Biol Evol. 2017:34(6):1445–1462. 10.1093/molbev/msx091. 28333217 PMC5435080

[evae024-B55] Laetsch DR , Bisschop G, Martin SH, Aeschbacher S, Setter D, Lohse K. Demographically explicit scans for barriers to gene flow using gIMble. PLoS Genet. 2023:19(10):1–30. 10.1371/journal.pgen.1010999. PMC10610087 37816069

[evae024-B56] Laetsch DR , Blaxter ML. BlobTools: interrogation of genome assemblies. F1000Res. 2017:6:1–16. 10.12688/f1000research.12232.1.

[evae024-B57] Lê S , Josse J, Husson F. FactoMineR: an R package for multivariate analysis. J Stat Softw. 2008:25(1):1–18. 10.18637/jss.v025.i01.

[evae024-B58] Li H . Minimap2: pairwise alignment for nucleotide sequences. Bioinformatics. 2018:34(18):3094–3100. 10.1093/bioinformatics/bty191. 29750242 PMC6137996

[evae024-B59] Li H , Durbin R. Fast and accurate short read alignment with Burrows-Wheeler transform. Bioinformatics. 2009:25(14):1754–1760. 10.1093/bioinformatics/btp324. 19451168 PMC2705234

[evae024-B60] Li H , Handsaker B, Wysoker A, Fennell T, Ruan J, Homer N, Marth G, Abecasis G, Durbin R. The Sequence Alignment/Map (SAM) format and SAMtools. Bioinformatics. 2009:25(16):2078–2079. 10.1093/bioinformatics/btp352. 19505943 PMC2723002

[evae024-B61] Lohse K , Clarke M, Ritchie MG, Etges WJ. Genome-wide tests for introgression between cactophilic *Drosophila* implicate a role of inversions during speciation. Evolution (N Y). 2015:69:1178–1190. 10.1111/evo.12650. PMC5029762 25824653

[evae024-B62] Lomsadze A , Burns PD, Borodovsky M. Integration of mapped RNA-seq reads into automatic training of eukaryotic gene finding algorithm. Nucleic Acids Res. 2014:42(15):e119. 10.1093/nar/gku557. 24990371 PMC4150757

[evae024-B63] Lowry DB , Willis JH. A widespread chromosomal inversion polymorphism contributes to a major life-history transition, local adaptation, and reproductive isolation. PLoS Biol. 2010:8(9):e1000500. 10.1371/journal.pbio.1000500. 20927411 PMC2946948

[evae024-B64] Lundberg M , Mackintosh A, Petri A, Bensch S. Inversions maintain differences between migratory phenotypes of a songbird. Nat Commun. 2023:14(1):452. 10.1038/s41467-023-36167-y. 36707538 PMC9883250

[evae024-B65] Macías LG , Barrio E, Toft C. GWideCodeML: a Python package for testing evolutionary hypotheses at the genome-wide level. G3 Genes, Genomes, Genet. 2020:10(12):4369–4372. 10.1534/g3.120.401874. PMC7718741 33093185

[evae024-B66] Mackintosh A , Vila R, Laetsch DR, Hayward A, Martin SH, Lohse K. Chromosome fissions and fusions act as barriers to gene flow between *Brenthis* fritillary butterflies. Mol Biol Evol. 2023:40(3):1–13. 10.1093/molbev/msad043. PMC10015618 36810615

[evae024-B67] Marçais G , Delcher AL, Phillippy AM, Coston R, Salzberg SL, Zimin A. MUMmer4: a fast and versatile genome alignment system. PLoS Comput Biol. 2018:14(1):1–14. 10.1371/journal.pcbi.1005944. PMC5802927 29373581

[evae024-B68] Matzkin LM , Merritt TJS, Zhu CT, Eanes WF. The structure and population genetics of the breakpoints associated with the cosmopolitan chromosomal inversion In(3R)Payne in *Drosophila melanogaster*. Genetics. 2005:170(3):1143–1152. 10.1534/genetics.104.038810. 15781702 PMC1451188

[evae024-B69] Michel AP , Grushko O, Guelbeogo WM, Lobo NF, Sagnon N, Costantini C, Besansky NJ. Divergence with gene flow in *Anopheles funestus* from the Sudan Savanna of Burkina Faso, West Africa. Genetics. 2006:173(3):1389–1395. 10.1534/genetics.106.059667. 16648581 PMC1526678

[evae024-B70] Miller DE , Staber C, Zeitlinger J, Hawley RS. Highly contiguous genome assemblies of 15 *Drosophila* species generated using nanopore sequencing. G3 Genes, Genomes, Genet. 2018:8(10):3131–3141. 10.1534/g3.118.200160. PMC6169393 30087105

[evae024-B71] Navarro A , Barton NH. Accumulating postzygotic isolation genes in parapatry: a new twist on chromosomal speciation. Evolution. 2003:57:447–459. 10.1111/j.0014-3820.2003.tb01537.x. 12703935

[evae024-B72] Noor MAF , Bennett SM. Islands of speciation or mirages in the desert? Examining the role of restricted recombination in maintaining species. Heredity (Edinb). 2009:103(6):439–444. 10.1038/hdy.2009.151. 19920849 PMC2809014

[evae024-B73] Noor MAF , Garfield DA, Schaeffer SW, Machado CA. Divergence between the *Drosophila pseudoobscura* and *D. persimilis* genome sequences in relation to chromosomal inversions. Genetics. 2007:177(3):1417–1428. 10.1534/genetics.107.070672. 18039875 PMC2147956

[evae024-B74] Noor MAF , Grams KL, Bertucci LA, Reiland J. Chromosomal inversions and the reproductive isolation of species. Proc Natl Acad Sci U S A. 2001:98(21):12084–12088. 10.1073/pnas.221274498. 11593019 PMC59771

[evae024-B75] Nozawa K , Garcia TX, Kent K, Leng M, Jain A, Malovannaya A, Yuan F, Yu Z, Ikawa M, Matzuk MM, et al Testis-specific serine kinase 3 is required for sperm morphogenesis and male fertility. Andrology. 2023:11(5):826–839. 10.1111/andr.13314. 36306217 PMC10267670

[evae024-B76] Parker DJ , Envall T, Ritchie MG, Kankare M. Sex-specific responses to cold in a very cold-tolerant, northern *Drosophila* species. Heredity (Edinb). 2021:126(4):695–705. 10.1038/s41437-020-00398-2. 33510465 PMC8182794

[evae024-B77] Parker DJ , Wiberg RA, Trivedi U, Tyukmaeva VI, Gharbi K, Butlin RK, Hoikkala A, Kankare M, Ritchie MG. Inter and intraspecific genomic divergence in *Drosophila montana* shows evidence for cold adaptation. Genome Biol Evol. 2018:10(8):2086–2101. 10.5061/dryad.s813p55. 30010752 PMC6107330

[evae024-B78] Patterson JT . Revision of the *montana* complex of the virilis species group. Univerisity Texas Publ. 1952:5204:20–34.

[evae024-B79] Poikela N , Kinnunen J, Wurdack M, Kauranen H, Schmitt T, Kankare M, Snook RR, Hoikkala A. Strength of sexual and postmating prezygotic barriers varies between sympatric populations with different histories and species abundances. Evolution. 2019:73(6):1182–1199. 10.1111/evo.13732. 30957216

[evae024-B80] Poikela N , Laetsch DR, Kankare M, Hoikkala A, Lohse K. Experimental introgression in *Drosophila*: asymmetric postzygotic isolation associated with chromosomal inversions and an incompatibility locus on the X chromosome. Mol Ecol. 2023:32(4):854–866. 10.1111/mec.16803. 36461113 PMC10107139

[evae024-B81] Poikela N , Tyukmaeva V, Hoikkala A, Kankare M. Multiple paths to cold tolerance: the role of environmental cues, morphological traits and the circadian clock gene vrille. BMC Ecol Evol. 2021:21(1):1–20. 10.1186/s12862-021-01849-y. 34112109 PMC8191109

[evae024-B82] Rausch T , Zichner T, Schlattl A, Stütz AM, Benes V, Korbel JO. DELLY: structural variant discovery by integrated paired-end and split-read analysis. Bioinformatics. 2012:28(18):i333–i339. 10.1093/bioinformatics/bts378. 22962449 PMC3436805

[evae024-B83] Ruan J , Li H. Fast and accurate long-read assembly with wtdbg2. Nat Methods. 2020:17(2):155–158. 10.1038/s41592-019-0669-3. 31819265 PMC7004874

[evae024-B84] Schaeffer SW , Bhutkar A, McAllister BF, Matsuda M, Matzkin LM, O'Grady PM, Rohde C, Valente VLS, Aguadé M, Anderson WW, et al Polytene chromosomal maps of 11 *Drosophila* species: the order of genomic scaffolds inferred from genetic and physical maps. Genetics. 2008:179(3):1601–1655. 10.1534/genetics.107.086074. 18622037 PMC2475758

[evae024-B85] Schäfer MA , Mazzi D, Klappert K, Kauranen H, Vieira J, Hoikkala A, Ritchie MG, Schlötterer C. A microsatellite linkage map for *Drosophila montana* shows large variation in recombination rates, and a courtship song trait maps to an area of low recombination. J Evol Biol. 2010:23(3):518–527. 10.1111/j.1420-9101.2009.01916.x. 20040000

[evae024-B86] Sedlazeck FJ , Rescheneder P, Smolka M, Fang H, Nattestad M, von Haeseler A, Schatz MC. Accurate detection of complex structural variations using single-molecule sequencing. Nat Methods. 2018:15(6):461–468. 10.1038/s41592-018-0001-7. 29713083 PMC5990442

[evae024-B87] Seppey M , Manni M, Zdobnow EM. BUSCO: assessing genome assembly and annotation completeness. Methods Mol Biol. 2019:1962:227–245. 10.1007/978-1-4939-9173-0_14. 31020564

[evae024-B88] Servedio MR , Noor MAF. The role of reinforcement in speciation: theory and data. Source Annu Rev Ecol Evol Syst. 2003:34(1):339–364. 10.1146/132412.

[evae024-B89] Smit AFA , Hubley R, Green P. RepeatMasker Open-4.0., 2013-2015. https://www.repeatmasker.org/faq.html.

[evae024-B90] Stanke M , Diekhans M, Baertsch R, Haussler D. Using native and syntenically mapped cDNA alignments to improve de novo gene finding. Bioinformatics. 2008:24(5):637–644. 10.1093/bioinformatics/btn013. 18218656

[evae024-B91] Stanke M , Schöffmann O, Morgenstern B, Waack S. Gene prediction in eukaryotes with a generalized hidden Markov model that uses hints from external sources. BMC Bioinformatics. 2006:7(1):62. 10.1186/1471-2105-7-62. 16469098 PMC1409804

[evae024-B92] Stone WS , Guest WC, Wilson FD. The evolutionary implications of the cytological polymorphism and phylogeny of the virilis group of *Drosophila*. Proc Natl Acad Sci. 1960:46(3):350–361. 10.1073/pnas.46.3.350. 16578491 PMC222839

[evae024-B93] Sturtevant AH . A case of rearrangement of genes in *Drosophila*. Proc Natl Acad Sci U S A. 1921:7(8):235–237. 10.1073/pnas.7.8.235. 16576597 PMC1084859

[evae024-B94] Tarasov A , Vilella AJ, Cuppen E, Nijman IJ, Prins P. Sambamba: fast processing of NGS alignment formats. Bioinformatics. 2015:31:2032–2034. 10.1093/bioinformatics/btv098. 25697820 PMC4765878

[evae024-B95] Thorvaldsdóttir H , Robinson JT, Mesirov JP. Integrative Genomics Viewer (IGV): high-performance genomics data visualization and exploration. Brief Bioinformatics. 2013:14(2):178–192. 10.1093/bib/bbs017. 22517427 PMC3603213

[evae024-B96] Throckmorton LH . The *virilis* species group. Genet Bioogy Drosoph. 1982:3:227–296.

[evae024-B97] Trickett AJ , Butlin RK. Recombination suppressors and the evolution of new species. Heredity (Edinb). 1994:73(4):339–345. 10.1038/hdy.1994.180. 7989214

[evae024-B98] Tyukmaeva VI , Lankinen P, Kinnunen J, Kauranen H, Hoikkala A. Latitudinal clines in the timing and temperature-sensitivity of photoperiodic reproductive diapause in *Drosophila montana*. Ecography (Cop). 2020:43:1–10. 10.1111/ecog.04892.

[evae024-B99] Vicoso B , Charlesworth B. Evolution on the X chromosome: unusual patterns and processes. Nat Rev Genet. 2006:7(8):645–653. 10.1038/nrg1914. 16847464

[evae024-B100] Villoutreix R , Ayala D, Joron M, Gompert Z, Feder JL, Nosil P. Inversion breakpoints and the evolution of supergenes. Mol Ecol. 2021:30(12):2738–2755. 10.1111/mec.15907. 33786937 PMC7614923

[evae024-B101] Walker BJ , Abeel T, Shea T, Priest M, Abouelliel A, Sakthikumar S, Cuomo CA, Zeng Q, Wortman J, Young SK, et al Pilon: an integrated tool for comprehensive microbial variant detection and genome assembly improvement. PLoS One. 2014:9(11):e112963. 10.1371/journal.pone.0112963. 25409509 PMC4237348

[evae024-B102] Wall JD . Estimating ancestral population sizes and divergence times. Genetics. 2003:163(1):395–404. 10.1093/genetics/163.1.395. 12586724 PMC1462435

[evae024-B103] Wallberg A , Schöning C, Webster MT, Hasselmann M. Two extended haplotype blocks are associated with adaptation to high altitude habitats in East African honey bees. PLoS Genet. 2017:13(5):e1006792. 10.1371/journal.pgen.1006792. 28542163 PMC5444601

[evae024-B104] Wellenreuther M , Bernatchez L. Eco-evolutionary genomics of chromosomal inversions. Trends Ecol Evol. 2018:33(6):427–440. 10.1016/j.tree.2018.04.002. 29731154

[evae024-B105] Wright D , Schaeffer SW. The relevance of chromatin architecture to genome rearrangements in *Drosophila*. Philos Trans R Soc B Biol Sci. 2022:377(1856):20210206. 10.1098/rstb.2021.0206. PMC9189500 35694744

[evae024-B106] Yang Z . Paml: a program package for phylogenetic analysis by maximum likelihood. Bioinformatics. 1997:13(5):555–556. 10.1093/bioinformatics/13.5.555. 9367129

[evae024-B107] Yang H , Jaime M, Polihronakis M, Kanegawa K, Markow T, Kaneshiro K, Oliver B. Re-annotation of eight *Drosophila* genomes. Life Sci Alliance. 2018:1(6):e201800156. 10.26508/lsa.201800156. PMC6305970 30599046

[evae024-B108] Yusuf L , Tyukmaeave V, Hoikkala A, Ritchie M. Divergence and introgression among the virilis group of *Drosophila*. bioRxiv. 10.1101/2022.01.11.475832. 2022. PMC9783487 36579165

[evae024-B109] Zimin AV , Puiu D, Luo M-C, Zhu T, Koren S, Marçais G, Yorke JA, Dvořák J, Salzberg SL. Hybrid assembly of the large and highly repetitive genome of *Aegilops tauschii*, a progenitor of bread wheat, with the MaSuRCA mega-reads algorithm. Genome Res. 2017:27(5):787–792. 10.1101/gr.213405.116. 28130360 PMC5411773

