# Supplementary material for: Chromosomal Inversions and the Demography of Speciation in Drosophila montana and Drosophila flavomontana
Source: Genome Biol Evol. 2024 Mar 14;16(3):evae024. doi: 10.1093/gbe/evae024 (PMC10972691; doi:10.1093/gbe/evae024)
Supplement: evae024_Supplementary_Data [file evae024_supplementary_data.zip › Chromosomal_inversions_and_speciation_supplementary_material_Poikela_etal_GBE_Revision_Clean.pdf]

Supplementary Material

Chromosomal inversions and the demography of speciation in *Drosophila montana* and *Drosophila flavomontana*

Poikela, N., Laetsch, D. R., Hoikkala, V., Lohse, K. & Kankare, M.

Contents

Supplementary Material 1
Supplementary tables 2
Supplementary figures 14
Supplementary references 36

Supplementary tables

Table S1. Detailed information of the samples. PacBio samples were sequenced using pools of females collected from isofemale lines that were kept in the fly laboratory since their establishment, and Illumina samples were sequenced using single wild-caught females or their F_1 daughters stored in -20 ˚C after the collections. Prefix "mon" and "fla" before a fly strain ID refer to *D. montana* and *D. flavomontana*, respectively.

Collecting site	Illumina sample	Strain ID	Generation	Collecting year	PacBio
Allopatry (Alaska)					
Fairbanks (Moose Creek), Alaska, USA<br>64˚55'26''N; 147˚59'17''W<br>Altitude 180m	Only *D. montana*	monFA13F2	F1 female	2013	
Honolulu (Honolulu Creek), Alaska, USA<br>63˚03'49''N; 149˚32'40''W<br>Altitude 495 m	Only *D. montana*	monHO13F3	F1 female	2013	
Seward, Alaska, USA<br>60˚09'45''N; 149˚27'09''W<br>Altitude 35 m	Only *D. montana*	monSE13F37	F1 female	2013	60 females from the isofemale strain
Sympatry (Rocky Mountains)					
McBride, British Columbia, Canada<br>53˚07'N; 120˚18'W<br>Altitude 720 m	Both species	monMB14F1<br>flaMB14F10	Founder female<br>Founder female	2014<br>2014	
Cranbrook, British Columbia, Canada<br>49˚36'N; 115˚46'W<br>Altitude 940 m	Both species	monCRAN14F16<br>flaCRAN14F7	Founder female<br>Founder female	2014<br>2014	
Livingston, Montana, USA<br>45˚20'59''N; 110˚36'30''W<br>Altitude 1 605 m	Only *D. flavomontana*	flaMT13F11	F1 female	2013	60 females from the isofemale strain
Jackson, Wyoming, USA<br>43˚25'48''N; 110˚50'34''W<br>Altitude 1 857 m	Both species	monJX13F3<br>flaJX13F38	F1 female<br>F1 female	2013<br>2013	60 females from the isofemale strain
Afton, Wyoming, USA<br>42˚43'N; 110˚55'W<br>Altitude 2 000 m	Only *D. montana*	monAF15F28	F1 female	2015	
Liberty, Utah, USA<br>41˚20'N; 111˚51'W<br>Altitude 1 600 m	Only *D. flavomontana*	flaLB15F3	F1 female	2015	
Sympatry (Western coast)					
Terrace, British Columbia, Canada<br>54˚27'N; 128˚34'W<br>Altitude 217 m	Both species	monTER14F11<br>flaTER14F5	Founder female<br>Founder female	2014<br>2014	
Vancouver, British Columbia, Canada<br>49°15'N;123°10'W<br>Altitude 4 m	Both species	monVAN14F1<br>flaVAN14F20	Founder female<br>Founder female	2014<br>2014	 60 females from the isofemale strain
Ashford, Washington, Usa<br>46˚45'21''N; 121˚57'22''W<br>Altitude 573 m	Both species	monASH13F13<br>flaASH13F2	F1 female<br>F1 female	2013<br>2013	
Fall Creek, Oregon, USA<br>43˚58'32''N; 122˚45'06''W<br>Altitude 225 m	Both species	mon4Fall<br>fla3Fall	F1 female<br>F1 female	2010<br>2010	
Azalea, Oregon, USA<br>42˚48'24''N; 123˚13'37''W<br>Altitude 498 m	Only *D. montana*	monAZA2	F1 female	2010	

Table S2. PacBio and Illumina raw reads. N50 raw reads = half of the raw reads are larger than or equal to the N50 raw read value.

**PacBio reads**				**Total number of reads**						**Number of reads**			
**Species**	**Region**	**Population**	**Strain ID**	**SMRT 1**	**SMRT 2**	**Mean coverage**	**Max read length (bp)**	**Average read length (bp)**	**N50 raw read (bp)**	**>= 80 000 bp**	**Bases (Gb)**	**Technique**	**Sequencing facility**
D. montana	Allopatry (Alaska)	Seward	monSE13F37	888,644	781,439	35.1	168303	5905.9	11366	81	9.9	PacBio Sequel	Norwegian sequencing center
	Sympatry (Rocky Mountains)	Jackson	monJX13F48	2,161,616	-	62.9	111203	6422.3	8045	19	13.9	PacBio Sequel	BGI
D. flavomontana	Sympatry (Rocky Mountains)	Livingston	flaMT13F11	684,309	758,824	27.1	194838	5390.7	10773	121	7.8	PacBio Sequel	Norwegian sequencing center
	Sympatry (Western coast)	Vancouver	flaVAN14F20	710,709	839,748	30.9	169754	5861.8	12235	133	9.1	PacBio Sequel	Norwegian sequencing center
**Illumina 150bp paired-end reads**				**Total number of reads**									
**Species**	**Region**	**Population**	**Strain ID**	**Lane 1**	**Lane 2**	**Mean coverage**	**Insert size peak (bp)**					**Technique**	**Sequencing facility**
D. montana	Allopatry (Alaska)	Seward	monSE13F37	315,326,680	-	434.6	261					HiSeq4000	Norwegian sequencing center
		Honolulu Creek	monHO13F3	48,321,573	-	72.7	255					HiSeq X-Ten	BGI
		Fairbanks	monFA13F2	52,970,348	-	79.9	251					HiSeq X-Ten	BGI
	Sympatry (Rocky Mountains)	McBride	monMB14F1	64,726,992	-	52.9	229					HiSeq X-Ten	BGI
		Cranbrook	monCAN14F16	53,005,731	-	75.2	257					HiSeq X-Ten	BGI
		Jackson	monJX13F48	33,029,719	31,973,224	81.9	150					HiSeq4000	Edinburgh Genomics
		Afton	monAF15F28	34,605,493	36,274,368	84.8	150					HiSeq4000	Edinburgh Genomics
	Sympatry (Western coast)	Terrace	monTER14F11	48,422,463	-	66.6	239					HiSeq X-Ten	BGI
		Vancouver	monVAN14F1	30,641,929	30,099,059	69.2	150					HiSeq4000	Edinburgh Genomics
		Ashford	monASH13F13	42,182,222	41,678,505	93.9	111					HiSeq4000	Edinburgh Genomics
		Fall Creek	mon4Fall	55,597,271	-	81.7	261					HiSeq X-Ten	BGI
		Azalea	monAZA2	47,287,149	-	70.2	250					HiSeq X-Ten	BGI
D. flavomontana	Sympatry (Rocky Mountains)	McBride	flaMB14F10	49,610,911	-	70.5	262					HiSeq X-Ten	BGI
		Cranbrook	flaCRAN14F7	54,276,116	-	70.6	258					HiSeq X-Ten	BGI
		Livingston	flaMT13F11	28,832,333	30,942,904	74.6	150					HiSeq4000	Edinburgh Genomics
		Jackson	flaJX13F31	41,741,562	42,330,188	109.1	150					HiSeq4000	Edinburgh Genomics
	Sympatry (Western coast)	Liberty	flaLB15F3	39,780,753	40,656,195	101.5	150					HiSeq4000	Edinburgh Genomics
		Terrace	flaTER14F5	50,162,958	-	71.9	150					HiSeq X-Ten	BGI
		Vancouver	flaVAN14F20	36,923,245	38,165,991	91.7	150					HiSeq4000	Edinburgh Genomics
		Ashford	flaASH13F2	23,396,198	22,877,448	58.6	150					HiSeq4000	Edinburgh Genomics
		Fall Creek	fla3Fall	50,824,988	-	73.0	150					HiSeq X-Ten	BGI

Table S3. Contiguity, completeness, and annotation of original and scaffolded chromosome-level genome assemblies. N50 = half of the genome is in contigs larger than or equal to the N50 contig size. N50 count = half of the genome consists of the number indicated in N50 count.

	D. montana Seward, Alaska monSE13F37	*D. montana* Jackson, USA monJX13F48	*D. flavomontana* Livingston, USA flaMT13F11	*D. flavomontana* Vancouver, Canada flaVAN14F20	*D. montana* chromosome-level genome	*D. flavomontana* chromosome-level genome
**Genome contiguity**						
**Genome size (Mb)**	184.3	181.0	192.6	193.5	145.5	148.1
**Total contigs**	324	796	376	296	6	6
**Longest contig (Mb)**	29.1	15.2	29.0	26.8	32.5	31.2
**N50 (Mb)**	11.0	1.3	9.8	7.8	26.5	28.8
**N50 count**	5	20	6	6	3	3
**Genome completeness**						
**Complete BUSCOs (%; n: 3285)**	98.1	98.5	97.5	97.3	91.9	93.5
**Single copy BUSCOs (%)**	97.6	98.0	97.1	96.9	91.7	93.2
**Duplicated BUSCOs (%)**	0.5	0.5	0.4	0.4	0.2	0.3
**Genome annotation**						
**GC level (%)**	40.2	40.2	40.2	40.1	40.3	40.3
**Masked repeats (%)**	26.6	25.5	29.9	30.4	16.8	16.3
**Genes (N)**	15,696	15,819	15,940	16,056	15,446	15,299
**Mean gene length (bp)**	1,640	1,637	1,568	1,564	1,571	1,566
**Complete BUSCOs (%; n: 3285)**	94.2	94.7	92.5	92.9	88.5	89.7
**Single copy BUSCOs (%)**	84.4	84.5	84.1	84.5	79.9	81.3
**Duplicated BUSCOs (%)**	9.8	10.2	8.4	8.4	8.8	8.4

Table S4. Principal components, Eigenvalues, variance, and cumulative variance of 12 *D. montana* and 9 *D. flavomontana* Illumina samples.

PC	Eigenvalue	Variance (%)	Cumulative variance (%)
PC1	14.0	50.4	50.4
PC2	1.1	4.0	54.5
PC3	1.0	3.7	58.1
PC4	1.0	3.5	61.6
PC5	0.9	3.4	65.1
PC6	0.9	3.2	68.3
PC7	0.9	3.2	71.5
PC8	0.9	3.1	74.6
PC9	0.9	3.1	77.7
PC10	0.9	3.1	80.8
PC11	0.9	3.1	83.9
PC12	0.8	3.0	86.9
PC13	0.7	2.4	89.3
PC14	0.5	1.8	91.1
PC15	0.5	1.6	92.7
PC16	0.4	1.5	94.3
PC17	0.4	1.5	95.7
PC18	0.4	1.5	97.2
PC19	0.4	1.4	98.6
PC20	0.4	1.4	100.0

Table S5. A List of 19 bioclimatic variables used in the PCA (WorldClim database v2.0, 2.5 min spatial resolution; dataset 1970-2000; www.worldclim.org) (Fick and Hijmans 2017).

Variable	Description
bio1	Annual mean temperature
bio2	Mean diurnal range (mean of monthly (max - min temperature))
bio3	Isothermality (bio2/bio7*100)
bio4	Temperature seasonality (standard deviation * 100)
bio5	Max temperature of the warmest month
bio6	Min temperature of the coldest month
bio7	Annual temperature range (bio5-bio6)
bio8	Mean temperature of the wettest quarter
bio9	Mean temperature of the driest quarter
bio10	Mean temperature of the warmest quarter
bio11	Mean temperature of the coldest quarter
bio12	Annual precipitation
bio13	Precipitation of the wettest month
bio14	Precipitation of the driest month
bio15	Precipitation seasonality (coefficient of variation)
bio16	Precipitation of the wettest quarter
bio17	Precipitation of the driest quarter
bio18	Precipitation of the warmest quarter
bio19	Precipitation of the coldest quarter

Table S6. 19 bioclimatic variables (explanations are in Table S5) for each site extracted from WorldClim database v2.1 using latitudinal and longitudinal coordinates (2.5 min spatial resolution; dataset 1970-2000; www.worldclim.org) (Fick and Hijmans 2017).

Population	Longitude	Altitude	Latitude	bio1	bio2	bio3	bio4	bio5	bio6	bio7	bio8	bio9	bio10	bio11	bio12	bio13	bio14	bio15	bio16	bio17	bio18	bio19
Fairbanks	-147.99	180.00	64.92	-2.84	11.05	23.49	1371.73	21.74	-25.33	47.06	14.00	-10.18	14.00	-19.01	304	49	8	52	135	33	135	50
Honolulu Creek	-149.54	495.00	63.06	-1.22	9.57	26.43	1027.63	19.13	-17.09	36.22	10.26	-1.91	12.16	-12.49	636	103	25	50	281	88	251	107
Seward	-149.54	35.00	60.16	0.68	6.54	29.29	605.24	13.48	-8.84	22.32	0.65	2.64	8.74	-5.60	1104	162	52	42	423	161	210	302
Terrace	-128.57	217.00	54.45	6.41	7.72	27.57	730.96	21.38	-6.63	28.01	1.75	13.43	15.32	-2.71	1379	210	50	52	578	157	170	487
Vancouver	-122.60	4.00	49.24	10.04	9.15	36.73	577.06	24.20	-0.70	24.90	3.52	17.02	17.09	3.06	1972	308	64	49	818	221	226	691
Ashford	-121.96	573.00	46.76	6.25	9.40	39.81	512.91	20.72	-2.88	23.60	1.02	12.65	13.00	0.69	2333	361	48	58	1057	199	213	964
Fall Creek	-122.75	225.00	43.98	10.93	11.96	45.11	524.28	27.29	0.78	26.51	5.27	17.47	17.70	5.00	1497	233	20	61	676	111	112	609
Azalea	-123.23	498.00	42.81	11.05	13.74	48.03	555.79	28.90	0.30	28.60	5.03	18.11	18.20	4.80	1145	196	13	70	559	62	71	507
McBride	-120.16	720.00	53.30	4.07	11.31	32.24	850.10	22.13	-12.94	35.07	4.04	0.10	14.23	-6.66	706	75	39	21	210	127	201	163
Cranbrook	-115.74	940.00	49.52	5.55	12.24	32.16	899.03	25.95	-12.10	38.06	14.11	0.93	16.50	-5.85	461	61	26	27	151	85	133	113
Livingston	-110.61	1605.00	45.36	5.13	14.06	38.05	836.35	25.90	-11.05	36.95	12.93	-4.85	15.66	-4.85	500	72	24	35	185	83	154	83
Jackson	-110.84	1875.00	43.43	3.51	15.73	38.05	924.38	26.55	-14.80	41.35	8.07	-1.79	14.87	-8.04	456	52	28	20	132	94	110	120
Afton	-110.92	2000.00	42.72	3.14	16.64	36.87	1030.70	27.49	-17.63	45.12	8.47	15.39	15.39	-10.24	466	53	30	16	140	104	104	112
Liberty	-111.89	1600.00	41.33	5.95	13.73	36.51	866.64	27.02	-10.59	37.61	4.65	16.83	16.83	-4.49	515	55	27	21	162	89	89	129

Table S7. Principal components, Eigenvalues, variance, and cumulative variance of bioclimatic variables.

PC	Eigenvalue	Variance (%)	Cumulative variance (%)
PC1	10.44	54.93	54.93
PC2	5.11	26.88	81.80
PC3	1.43	7.55	89.35
PC4	0.71	3.76	93.11
PC5	0.54	2.86	95.98
PC6	0.46	2.44	98.42
PC7	0.14	0.76	99.17
PC8	0.10	0.51	99.68
PC9	0.05	0.24	99.92
PC10	0.01	0.04	99.97
PC11	0.00	0.02	99.98
PC12	0.00	0.01	100.00
PC13	0.00	0.00	100.00

Table S8. Contributions (loadings) of the bioclimatic variables (see also Table S5-S6) on each principal component (PC).

Variable	PC1	PC2	PC3	PC4	PC5
bio1	4.89	8.34	0.59	0.29	5.21
bio2	2.19	11.82	3.21	0.29	0.50
bio3	2.25	11.84	0.04	4.12	0.93
bio4	8.23	0.39	0.67	0.15	6.01
bio5	0.10	18.16	0.38	7.42	0.10
bio6	8.15	1.87	0.01	2.70	5.19
bio7	8.24	0.87	0.19	8.88	3.99
bio8	5.88	0.82	6.64	2.34	19.88
bio9	4.75	4.00	3.41	0.01	27.41
bio10	0.14	15.23	0.05	5.93	1.23
bio11	7.30	3.59	0.14	2.11	6.41
bio12	8.78	0.38	0.62	4.68	1.12
bio13	8.60	0.29	2.60	4.66	0.89
bio14	3.68	5.16	20.32	4.30	0.51
bio15	3.12	0.03	44.66	0.04	0.98
bio16	8.50	0.19	3.23	4.07	1.75
bio17	5.88	3.19	11.31	7.15	0.06
bio18	0.74	13.84	0.03	7.23	14.33
bio19	8.57	0.00	1.89	3.62	3.51

Table S9. Genomic coordinates of alternatively fixed chromosomal inversions of *D. montana* and *D. flavomontana*, based on the original assemblies of the species as well as the chromosome-level genomes of both species (Table S3). Each of the five alternatively fixed inversions as well as one putative heterozygous inversion are shown in their own line. The breakpoints are named proximal and distal based on their distance to the centromere (the approximate locations of the centromeres were identified using *D. virilis* chromosome maps and genes, see Methods for details).

		**Inversion breakpoints in *D. montana* and *D. flavomontana* genomes**					**Inversion breakpoints in *D. montana* chromosome-level genome**					**Inversion breakpoints in *D. flavomontana* chromosome-level genome**					
				**Breakpoint**					**Breakpoint**					**Breakpoint**			
**Species the inversion belongs to**	**Chromosome**	**Contig**	**Contig length (bp)**	**Distal**	**Proximal**	**Size (bp)**	**Scaffold**	**Scaffold length**	**Distal**	**Proximal**	**Size (bp)**	**Scaffold**	**Scaffold length**	**Distal**	**Proximal**	**Size (bp)**	**Representation in Illumina samples**
D. montana	X	monSE13F37.00001	29140820	3993916	11173058	7179142	X_chromosome	29140820	3993916	11173058	7179143	X_chromosome	28975156	6309450	20284519	13975070	homozygous in all *D. montana* samples
		monJX13F48.00118, monJX13F48.00006	253764, 4625534	104108	1345305	-											
		flaMT13F11.00001	28975156	6309450	20284519	13975069											
		flaVAN14F20.00001	26778568	22735439	8730382	14005057											
D. flavomontana	X	monSE13F37.00001	28975156	6041987	20201805	14159819	X_chromosome	29140820	6041987	20201805	14159819	X_chromosome	28975156	11363874	22321964	10958091	homozygous in all *D. montana* samples
		monJX13F48.00021, monJX13F48.00002	1229388, 13454835	142107	6726561	-											
		flaMT13F11.00001	28975156	11363874	22321964	10958091											
		flaVAN14F20.00001	26778568	17674592	6694000	10980592											
D. flavomontana	X	monSE13F37.00001	29140820	17044685	20200540	3155884	X_chromosome	29140820	17044685	20200540	3155884	X_chromosome	28975156	11368068	14486661	3118594	homozygous in all *D. flavomontana* samples
		monJX13F48.00021, monJX13F48.00002	1229388, 13454835	144296	9907081	-											
		flaMT13F11.00001	28975156	11368068	14486661	3119573											
		flaVAN14F20.00001	26778568	17672247	14539822	3132425											
D. montana	2L	monSE13F37.00002	20245637	15098446	19046306	3947861	2L_chromosome	20245637	15098446	19046306	3947861	2L_chromosome	20392248	1181134	5145967	3964834	heterozygous in all *D. montana* samples
		monJX13F48.00003	9138818	8048854	4117707	3931148											
		flaMT13F11.00005	10178141	1181134	5145967	3964833											
		flaVAN14F20.00004, flaVAN14F20.00047	17678878, 659972	2535965	477262	-											
D. flavomontana	4	monSE13F37.00004, monSE13F37.00009	12274228, 2530810	10263367	1906955	-	4_chromosome	32544174	17382172	3246708	14135465	4_chromosome	30698533	23745872	7855585	15890288	homozygous in all *D. flavomontana* samples,
Named J in Stone et al. 1960		monJX13F48.00036, monJX13F48.00073	596273, 350243	226846	284790	-											
		flaMT13F11.00003, flaMT13F11.00008	19965754, 5486619	12355487	1949285	-											
		flaVAN14F20.00003, flaVAN14F20.00008	19125027, 4169626	12375436	1985368	-											
		lummei.00004*	18946740	1568294	15689984	14121690											
D. flavomontana	5	monSE13F37.00003	20245037	7861561	17088002	9226442	5_chromosome	26508887	9422591	18649749	9227159	5_chromosome	27217941	21134310	11949884	9184427	homozygous in all *D. flavomontana* samples
Named E in Stone et al. 1960		flaMT13F11.00002	23523717	17439995	8255383	9183122											
		flaVAN14F20.00002	22345759	16280386	7084336	9194450											
		monJX13F48.00001	15164525	11733609	2455274	9278336											

* *D. flavomontana* inversion breakpoints on the 4th chromosome are found in different contigs in *D. montana* and *D. flavomontana* assemblies, and thus the inversion was verified using *D. lummei* genome.

Table S10. Contigs of each assembly (>1Mb contigs shown here) were assigned to chromosomes using *Drosophila lummei* genome (Poikela et al. in prep.).

**Seward *D. montana***			**Jackson *D. montana***			**Livingston *D. flavomontana***			**Vancouver *D. flavomontana***		
**Contig length (Mb)**		**Chromosome**	**Contig length (Mb)**		**Chromosome**	**Contig length (Mb)**		**Chromosome**	**Contig length (Mb)**		**Chromosome**
monSE13F37.00001	29140820	X	monJX13F48.00001	15164525	5	flaMT13F11.00001	28975156	X	flaVAN14F20.00001	26778568	X
monSE13F37.00002	20245637	2L	monJX13F48.00002	13454835	X	flaMT13F11.00002	23523717	5	flaVAN14F20.00002	22345759	5
monSE13F37.00003	19534505	5	monJX13F48.00003	9138818	2L	flaMT13F11.00003	19965754	4	flaVAN14F20.00003	19125027	4
monSE13F37.00004	12274228	4	monJX13F48.00004	8789853	2L	flaMT13F11.00004	10393190	2R	flaVAN14F20.00004	17678878	2L
monSE13F37.00005	10997687	2R	monJX13F48.00005	8412689	3	flaMT13F11.00005	10178141	2L	flaVAN14F20.00005	10603604	2R
monSE13F37.00006	5767499	3	monJX13F48.00006	4625534	X	flaMT13F11.00006	9776843	2L	flaVAN14F20.00006	7750958	3
monSE13F37.00007	3775224	3	monJX13F48.00007	3696986	5	flaMT13F11.00007	7754770	3	flaVAN14F20.00007	4798025	3
monSE13F37.00008	2568140	4	monJX13F48.00008	3308613	2R	flaMT13F11.00008	5486619	4	flaVAN14F20.00008	4169626	4
monSE13F37.00009	2530810	4	monJX13F48.00009	3289462	4	flaMT13F11.00009	3073694	3	flaVAN14F20.00009	3314633	3
monSE13F37.00010	2178246	3	monJX13F48.00010	3232620	-	flaMT13F11.00010	2473142	3	flaVAN14F20.00010	2593425	3
monSE13F37.00011	1979467	5	monJX13F48.00011	2813930	2R	flaMT13F11.00011	2460368	3	flaVAN14F20.00011	2245353	2L
monSE13F37.00012	1726930	4	monJX13F48.00012	2122920	2R	flaMT13F11.00012	2261038	3	flaVAN14F20.00012	2071253	4
monSE13F37.00013	1610333	-	monJX13F48.00013	2080494	2R	flaMT13F11.00013	2022541	3	flaVAN14F20.00013	1841750	3
monSE13F37.00014	1585447	4	monJX13F48.00014	1980436	5	flaMT13F11.00014	1824522	-	flaVAN14F20.00014	1714737	4
monSE13F37.00015	1444893	3	monJX13F48.00015	1656297	3	flaMT13F11.00015	1668200	3	flaVAN14F20.00015	1374044	-
monSE13F37.00016	1385966	3	monJX13F48.00016	1503212	4	flaMT13F11.00016	1643239	2L	flaVAN14F20.00016	1368565	-
monSE13F37.00017	1362064	4	monJX13F48.00017	1455675	3	flaMT13F11.00017	1376368	-	flaVAN14F20.00017	1336510	X
monSE13F37.00018	1364004	-	monJX13F48.00018	1408546	-	flaMT13F11.00018	1364617	-	flaVAN14F20.00018	1295148	-
monSE13F37.00019	1327839	-	monJX13F48.00019	1365292	4	flaMT13F11.00019	1346930	2L	flaVAN14F20.00019	1292286	-
monSE13F37.00020	1290151	-	monJX13F48.00020	1284102	-	flaMT13F11.00020	1305735	-	flaVAN14F20.00020	1278608	2L
monSE13F37.00021	1271459	4				flaMT13F11.00021	1205103	-	flaVAN14F20.00021	1168480	-
monSE13F37.00022	1212424	-				flaMT13F11.00022	1163332	-	flaVAN14F20.00022	1144968	3
monSE13F37.00023	1188514	3				flaMT13F11.00023	1128347	4	flaVAN14F20.00023	1099907	3
monSE13F37.00024	1154756	4				flaMT13F11.00024	1089159	-	flaVAN14F20.00024	1085779	-
monSE13F37.00025	1124584	-							flaVAN14F20.00025	1074364	-
monSE13F37.00026	1122473	-							flaVAN14F20.00026	1042346	-
monSE13F37.00027	1100476	X									
monSE13F37.00028	1088638	3									
monSE13F37.00029	1086808	4									
monSE13F37.00030	1052805	3									
monSE13F37.00031	1043766	-									

Table S11. Summary of the effects of chromosome partition (colinear vs inverted) on d_{xy} and F_{st} in allopatric and sympatric populations of *D. montana* and *D. flavomontana* and in intraspecific comparisons within *D. montana*. d_{xy} and F_{st} was analysed separately for the autosomes and the X chromosome and for intergenic, intronic and coding sequences. P-values were inferred from simulations and significant P-values are in bold.

Genomic region	Comparison	Comparison	dxy	P-value	Fst	P-value
**Intergenic**	**Allopatry**	[Colinear autosomes]	0.03030		0.5968	
		4 inverted	0.03511	**< 0.001**	0.6072	0.656
		5 inverted	0.03113	**< 0.001**	0.6200	0.278
		[Colinear X]	0.03211		0.7021	
		X inverted	0.03505	**< 0.001**	0.7273	0.942
	**Sympatry**	[Colinear autosomes]	0.03004		0.5857	
		4 inverted	0.03495	**< 0.001**	0.5930	0.752
		5 inverted	0.03092	**< 0.001**	0.6110	0.290
		[Colinear X]	0.03194		0.6876	
		X inverted	0.03484	**< 0.001**	0.6847	0.050
**Introns**	**Allopatry**	[Colinear autosomes]	0.03127		0.6042	
		4 inverted	0.03676	**< 0.001**	0.6114	0.596
		5 inverted	0.03333	**< 0.001**	0.6160	0.494
		[Colinear X]	0.03510		0.7191	
		X inverted	0.03824	**< 0.001**	0.7233	0.928
	**Sympatry**	[Colinear autosomes]	0.03102		0.5959	
		4 inverted	0.03663	**< 0.001**	0.5919	0.190
		5 inverted	0.03312	**0.018**	0.6083	0.532
		[Colinear X]	0.03498		0.7052	
		X inverted	0.03799	**< 0.001**	0.6798	**0.034**
**Coding**	**Allopatry**	[Colinear autosomes]	0.02025		0.6472	
		4 inverted	0.02206	**< 0.001**	0.6636	0.534
		5 inverted	0.01997	0.634	0.6405	0.208
		[Colinear X]	0.01694		0.7570	
		X inverted	0.01945	**< 0.001**	0.7400	0.104
	**Sympatry**	[Colinear autosomes]	0.02014		0.6394	
		4 inverted	0.02203	**< 0.001**	0.6472	0.654
		5 inverted	0.01989	0.516	0.6330	0.202
		[Colinear X]	0.01678		0.7494	
		X inverted	0.01925	**< 0.001**	0.7078	0.064

Table S12. Support (measured as ΔlnCL) and parameter estimates for divergence time (T), migration rate (M) and effective population sizes (N_e) for studied populations and their common ancestral population under strict divergence (DIV, M=0) and isolation with migration (IM) models with both gene flow directions. This initial model comparison was performed for intergenic autosomal colinear regions to minimize the effects of selection, using two reference genomes (*D. montana* and *D. flavomontana*) and two block sizes (64b and 128b). Grey shading indicates the best-fit model for each comparison (see Fig. S18-S19).

Reference genome used	Block size	Comparison	Model	*D. mon* N_e	*D. fla* N_e	Ancestral N_e	T	m	lnCL	ΔlnCL
D. montana	64 bp	Allopatric comparison	DIV	692600	395250	1464316	2378570	-	-45651205	12869
		(*D. mon* vs *D. fla*)	IM *D. mon --> D. fla*	705262	382053	1402532	2538521	1.09E-08	-45638336	0
			IM *D. fla --> D. mon*	691899	395700	1457419	2398028	1.21E-09	-45650952	12616
		Sympatric comparison	DIV	719795	392311	1458664	2342807	-	-136875659	47790
		(*D. mon* vs *D. fla*)	IM *D. mon --> D. fla*	735267	376923	1387707	2526063	1.29E-08	-136827869	0
			IM *D. fla --> D. mon*	718542	393103	1447342	2375536	2.13E-09	-136873722	45853
			**Model**	***D. mon allop*** **N_e**	***D. mon symp*** **N_e**	**Ancestral N_e**	**T**	**M**	**lnCL**	**ΔlnCL**
		Intraspecific comparison	DIV	1086549	1560399	858472	209661	-	-39887382	0
		(*D. mon* vs *D. mon*)	IM *D. mon symp. --> D. mon allop.*	1086577	1560435	858470	209663	1.50E-15	-39887382	0
			IM *D. mon allop. --> D. mon symp.*	1079043	1441403	857987	216639	3.32E-07	-39887408	27
	**Block size**		**Model**	***D. mon*** **N_e**	***D. fla*** **N_e**	**Ancestral N_e**	**T**	**M**	**lnCL**	**ΔlnCL**
	128 bp	Allopatric comparison	DIV	728545	398774	1132250	2710977	-	-26255962	15401
		(*D. mon* vs *D. fla*)	IM *D. mon --> D. fla*	732787	388220	1034471	2881985	7.62E-09	-26240561	0
			IM *D. fla --> D. mon*	725401	398056	1078665	2805472	3.40E-09	-26249397	8836
		Sympatric comparison	DIV	757078	395703	1134780	2671276	-	-78733822	56290
		(*D. mon* vs *D. fla*)	IM *D. mon --> D. fla*	762355	383306	1022312	2868134	9.05E-09	-78677532	0
			IM *D. fla --> D. mon*	752964	394863	1070200	2787162	4.37E-09	-78707531	29999
			**Model**	***D. mon allop*** **N_e**	***D. mon symp*** **N_e**	**Ancestral N_e**	**T**	**M**	**lnCL**	**ΔlnCL**
		Intraspecific comparison	DIV	1638756	2453210	776166	281292	-	-26289327	14070
		(*D. mon* vs *D. mon*)	IM *D. mon symp. --> D. mon allop.*	417656	1269040	704689	751648	5.86E-06	-26280840	5583
			IM *D. mon allop. --> D. mon symp.*	1041239	642010	696082	917483	4.42E-06	-26275257	0
**Reference genome used**	**Block size**	**Comparison**	**Model**	***D. mon*** **N_e**	***D. fla*** **N_e**	**Ancestral N_e**	**T**	**M**	**lnCL**	**ΔlnCL**
D. flavomontana	64 bp	Allopatric comparison	DIV	704135	356493	1462359	2399150	-	-46064633	9057
		(*D. mon* vs *D. fla*)	IM *D. mon --> D. fla*	713873	346500	1415033	2518428	7.81E-09	-46055576	0
			IM *D. fla --> D. mon*	703666	356694	1455996	2416828	1.01E-09	-46064391	8816
		Sympatric comparison	DIV	753925	352945	1439661	2344076	-	-138110861	35847
		(*D. mon* vs *D. fla*)	IM *D. mon --> D. fla*	767091	340667	1381988	2487543	9.83E-09	-138075014	0
			IM *D. fla --> D. mon*	752758	353451	1425934	2383428	2.43E-09	-138107834	32820
			**Model**	***D. mon allop*** **N_e**	***D. mon symp*** **N_e**	**Ancestral N_e**	**T**	**M**	**lnCL**	**ΔlnCL**
		Intraspecific comparison	DIV	1092818	2805737	826433	173390	-	-37761907	54
		(*D. mon* vs *D. mon*)	IM *D. mon symp. --> D. mon allop.*	1092873	2806425	826437	173381	1.27E-11	-37761907	54
			IM *D. mon allop. --> D. mon symp.*	1079681	2499743	825799	180657	5.24E-07	-37761853	0
	**Block size**		**Model**	***D. mon*** **N_e**	***D. fla*** **N_e**	**Ancestral N_e**	**T**	**M**	**lnCL**	**ΔlnCL**
	128 bp	Allopatric comparison	DIV	745937	359180	1130565	2725366	-	-26607436	11739
		(*D. mon* vs *D. fla*)	IM *D. mon --> D. fla*	749783	350820	1050428	2862796	5.92E-09	-26595697	0
			IM *D. fla --> D. mon*	744008	358222	1086957	2800850	2.49E-09	-26602466	6769
		Sympatric comparison	DIV	799335	355370	1118044	2674056	-	-79835822	44004
		(*D. mon* vs *D. fla*)	IM *D. mon --> D. fla*	804432	345201	1022931	2836521	7.25E-09	-79791818	0
			IM *D. fla --> D. mon*	796338	354096	1060491	2776345	3.62E-09	-79813016	21198
			**Model**	***D. mon allop*** **N_e**	***D. mon symp*** **N_e**	**Ancestral N_e**	**T**	**M**	**lnCL**	**ΔlnCL**
		Intraspecific comparison	DIV	1910759	5565491	742244	253322	-	-25123546	17638
		(*D. mon* vs *D. mon*)	IM *D. mon symp. --> D. mon allop.*	253437	1469856	661231	717155	1.57E-05	-25111917	6009
			IM *D. mon allop. --> D. mon symp.*	1051701	887758	654390	898887	5.80E-06	-25105908	0

Table S13. Genomes of the initial PacBio assemblies. N50 = half of the genome is in contigs larger than or equal the N50 contig size. N50 count = half of the genome consists of the number indicated in N50 count.

Assembler		*D. montana* Seward, Alaska monSE13F37	*D. montana* Jackson, USA monJX13F48	*D. flavomontana* Vancouver, Canada flaVAN14F20	*D. flavomontana* Livingston, USA flaMT13F11
**wtdbg2 (RedBean)**	**Genome size (Mb)**	199.9	181.3	212.5	207.0
	**Total contigs**	1002	1168	1780	1124
	**Longest contig (Mb)**	20.3	14.5	19.8	28.8
	**N50 (Mb)**	2.5	0.9	2.7	2.6
	**N50 count**	15	41	12	12
	**GC%**	0.417	0.404	0.410	0.406
**MaSuRCA**	**Genome size (Mb)**	199.1	185.6	202.5	200.8
	**Total contigs**	873	1251	733	845
	**Longest contig (Mb)**	16.5	5.5	16.1	15.6
	**N50 (Mb)**	1.7	0.5	2.2	2.1
	**N50 count**	25	86	15	26
	**GC%**	0.408	0.403	0.400	0.401

Table S14. Genome assemblies of single wild-caught females (Illumina 150b paired-end data) used in dN/dS analysis. N50 = half of the genome is in contigs larger than or equal the N50 contig size. N50 count = half of the genome consists of the number indicated in N50 count.

**Species**	**Population**		**Fly strain**	**Genome size (Mb)**	**Total contigs**	**Longest contig (bp)**	**N50 (bp)**	**N50 count**	**GC%**	**Total complete BUSCOs (%, n: 3285)**	**Single copy BUSCOs (%)**	**Duplicated BUSCOs (%)**	**Sample used in dN/dS analysis**
D. littoralis	Outgroup (Finland)	Korpilahti	KL13F60	205.1	23756	642147	67694	751	40.5	98.4	97.7	0.7	yes
D. montana	Sympatry (western coast)	Terrace	monTER14F11	218.3	32634	680450	41496	1386	41.0	98.0	97.2	0.8	yes
		Vancouver	monVAN14F1	205.8	50249	127841	11406	4956	40.5	92.2	89.6	2.6	yes
		Ashford	monASH13F13	225.3	119802	52723	3986	15697	40.3	79.9	78.8	1.1	no
		Fall Creek	mon4Fall	206.6	28369	423492	45341	1191	40.4	97.8	97.1	0.7	yes
		Azalea	monAZA2	210.1	32250	428221	36497	1498	40.5	97.9	97.4	0.5	yes
	Sympatry (Rocky Mountains)	McBride	monMB14F1	216.9	59047	372247	17321	3115	41.0	95.6	93.8	1.8	yes
		Cranbrook	monCRAN14F16	228.2	47760	728611	37548	1476	41.4	97.9	97.0	0.9	yes
		Jackson	monJX13F3	224.4	57826	181731	11395	5290	40.5	92.4	89.2	3.2	yes
		Afton	monAF15F28	227.5	73398	316902	9702	6161	40.6	91.7	90.0	1.7	yes
D. flavomontana	Sympatry (western coast)	Terrace	flaTER14F5	209.4	28746	745447	58956	906	40.2	98.2	97.6	0.6	yes
		Vancouver	flaVAN14F20	203.4	49591	177737	13290	4092	40.4	93.2	91.8	1.4	yes
		Ashford	flaASH13F2	202.2	40399	165684	13556	4091	40.7	95.4	94.2	1.2	yes
		Fall Creek	fla3Fall	203.1	29004	550168	50722	1011	40.3	97.7	97.0	0.7	yes
	Sympatry (Rocky Mountains)	McBride	flaMB14F10	227.1	41561	588908	52916	1018	41.8	98.0	97.3	0.7	yes
		Cranbrook	flaCRAN14F7	212.8	32100	613853	50615	1062	40.5	98.0	97.5	0.5	yes
		Livingston	flaMT13F11	204.6	65403	100004	7933	7202	40.6	83.2	80.5	2.7	no
		Jackson	flaJX13F38	212.8	49728	213613	13230	4408	40.6	90.9	88.3	2.6	yes
		Liberty	flaLB15F3	205.2	52507	159220	10707	5293	40.3	83.8	80.6	3.2	no

Supplementary figures

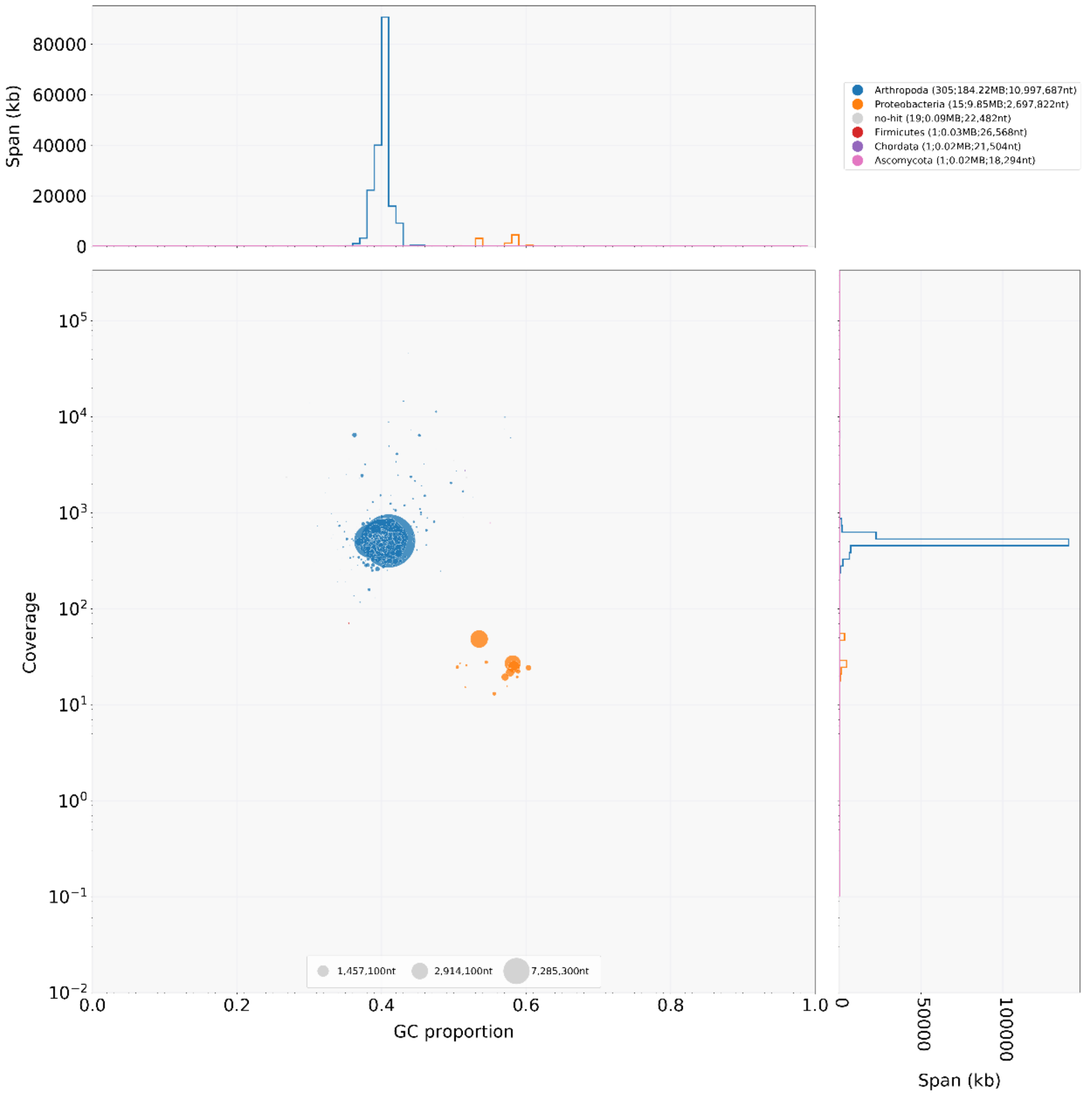

Figure S1. Genomic contaminants of monSE13F37 (*D. montana*) assembly illustrated with blobplot (Laetsch and Blaxter 2017).

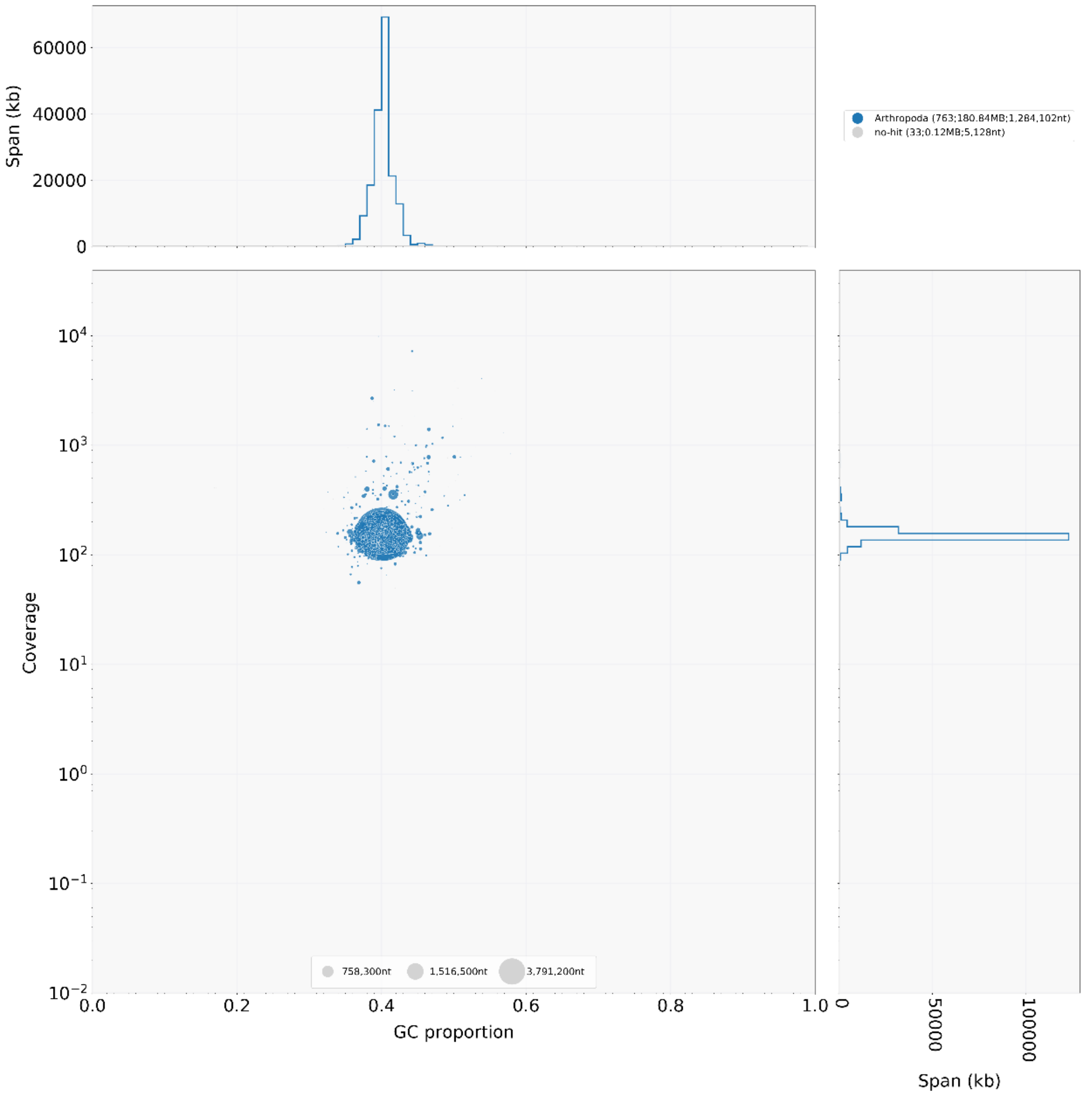

Figure S2. Genomic contaminants of monJX13F48 (*D. montana*) assembly illustrated with blobplot (Laetsch and Blaxter 2017).

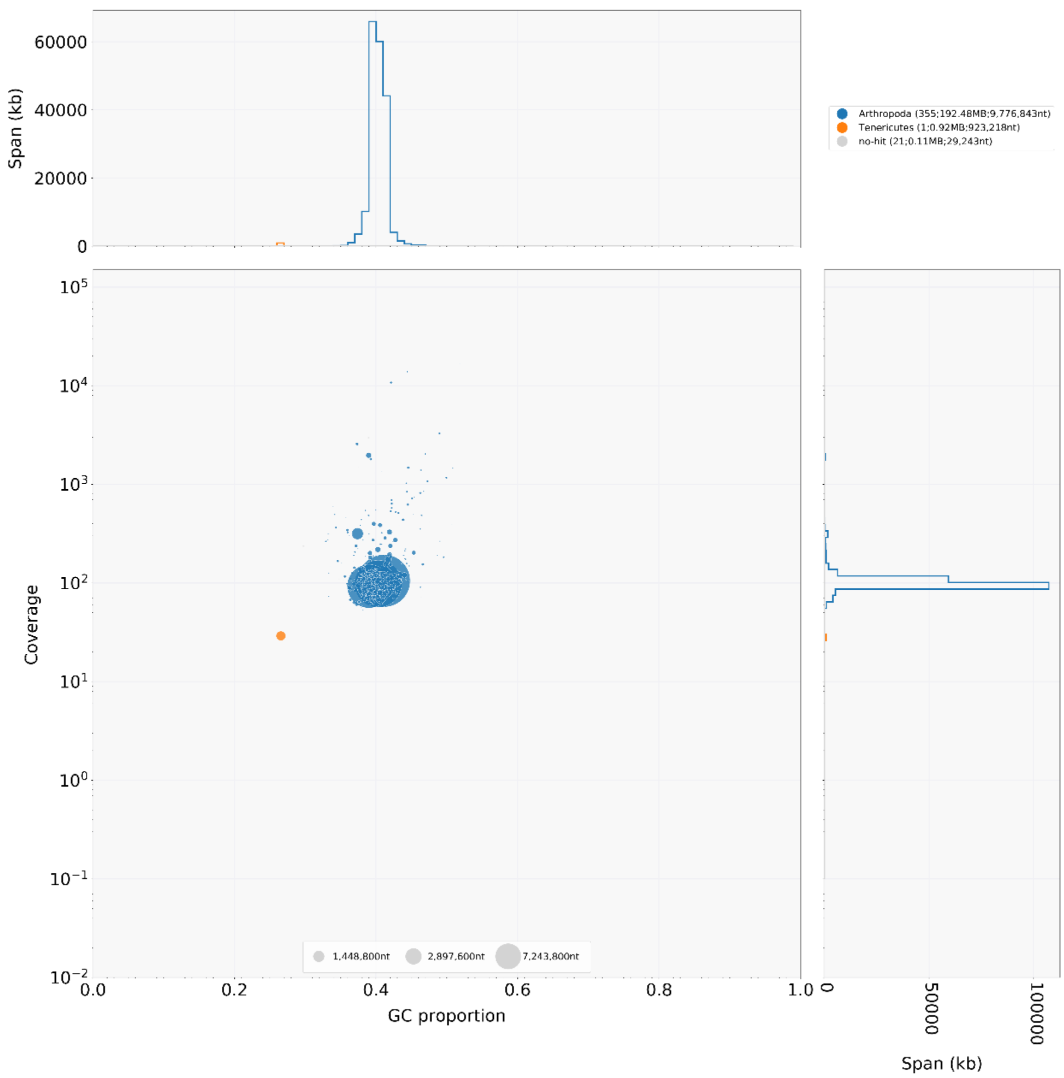

Figure S3. Genomic contaminants of flaMT13F11 (*D. flavomontana*) assembly illustrated with blobplot (Laetsch and Blaxter 2017).

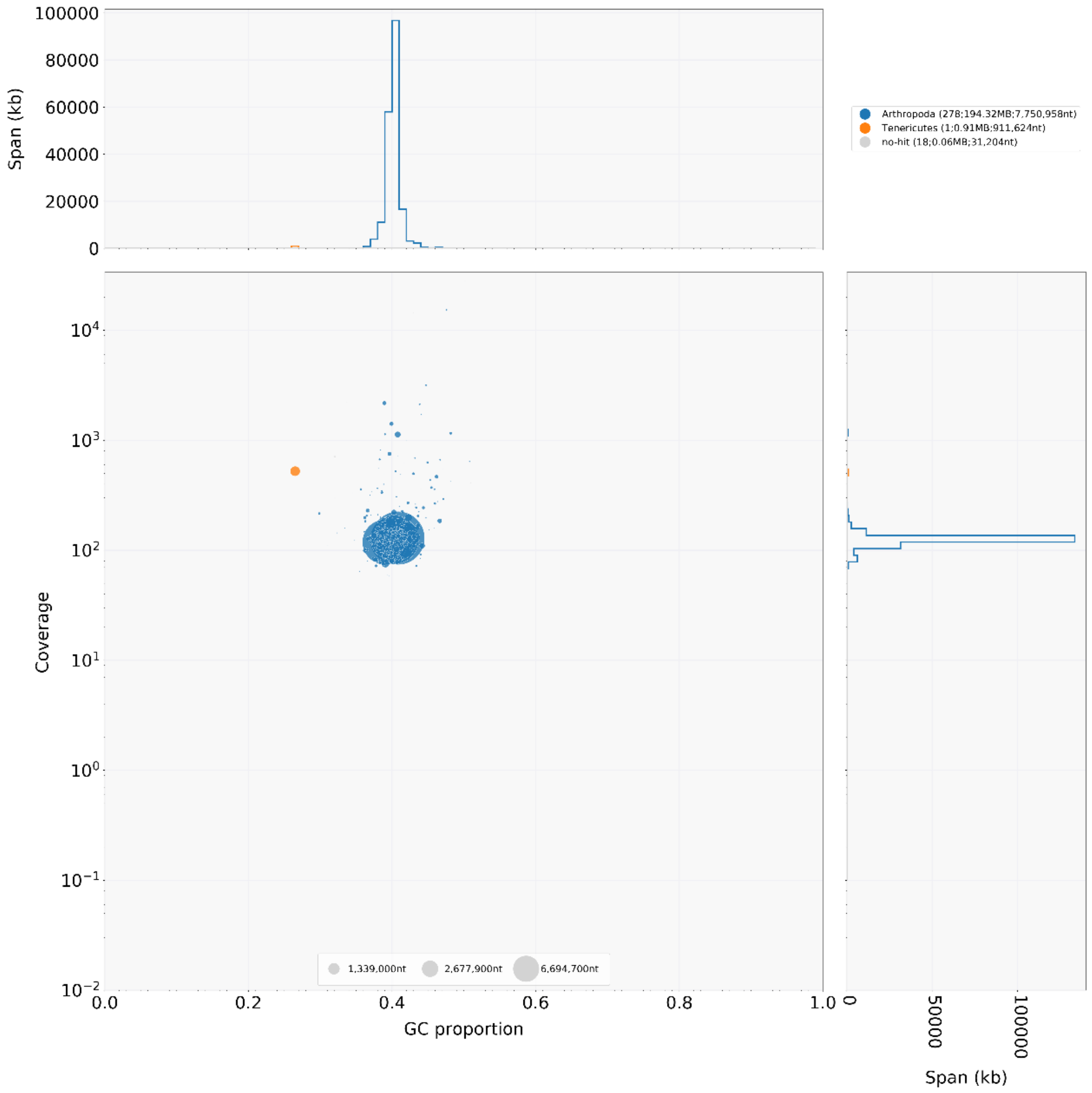

Figure S4. Genomic contaminants of flaVAN14F20 (*D. flavomontana*) assembly illustrated with blobplot (Laetsch and Blaxter 2017).

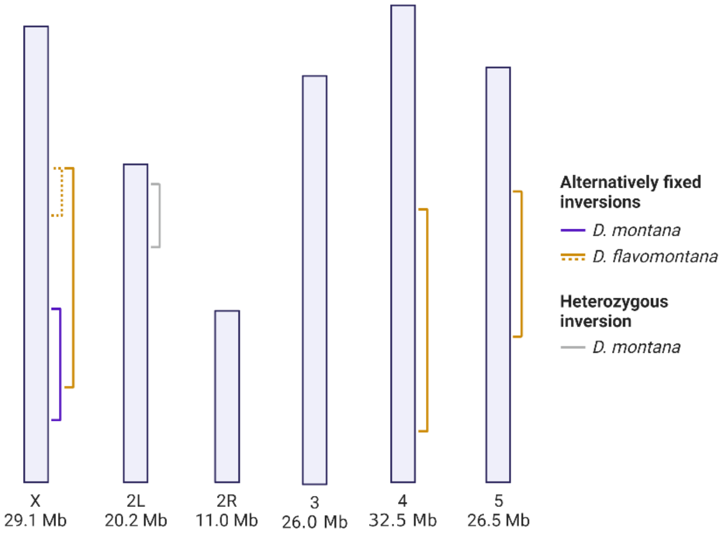

Figure S5. Chromosomes and five large (>0.5Mb) alternatively fixed inversions of *D. montana* (blue solid line) and *D. flavomontana* (orange solid and dashed lines), as well as one putative heterozygous inversion of *D. montana* (light grey solid line) (Table S9). The numbers below each chromosome refer to its length in the *D. montana* assembly. Chromosome 2 has left (2L) and right (2R) arms that are separated by a submetacentric centromere.

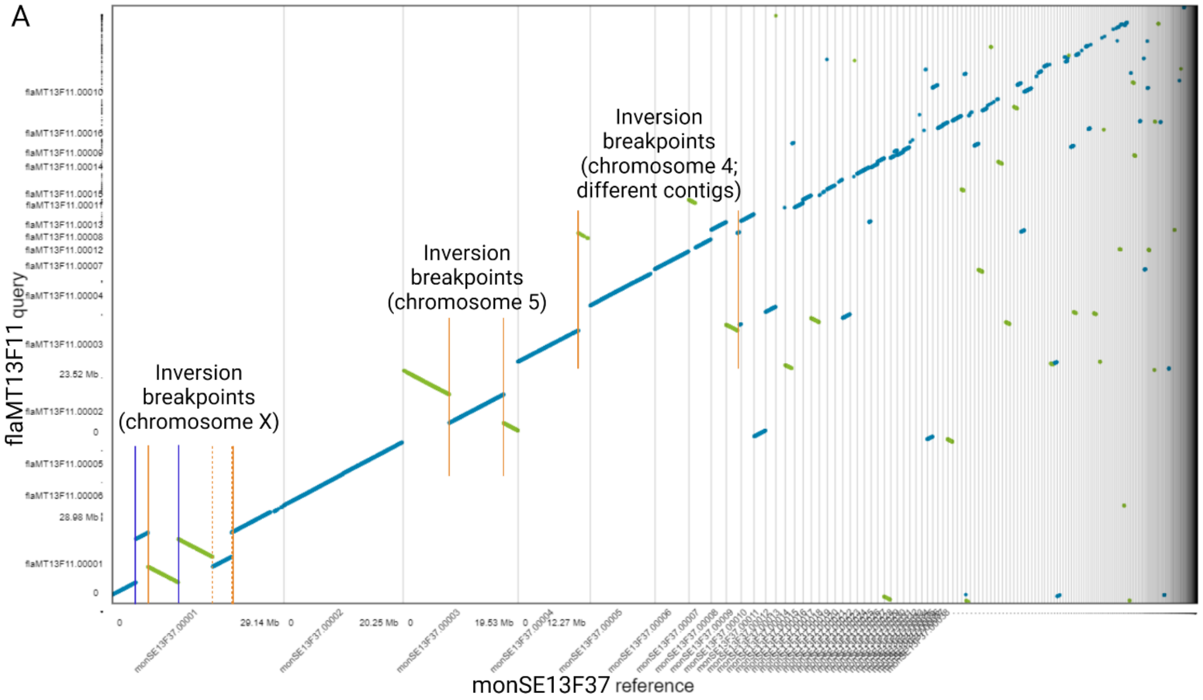

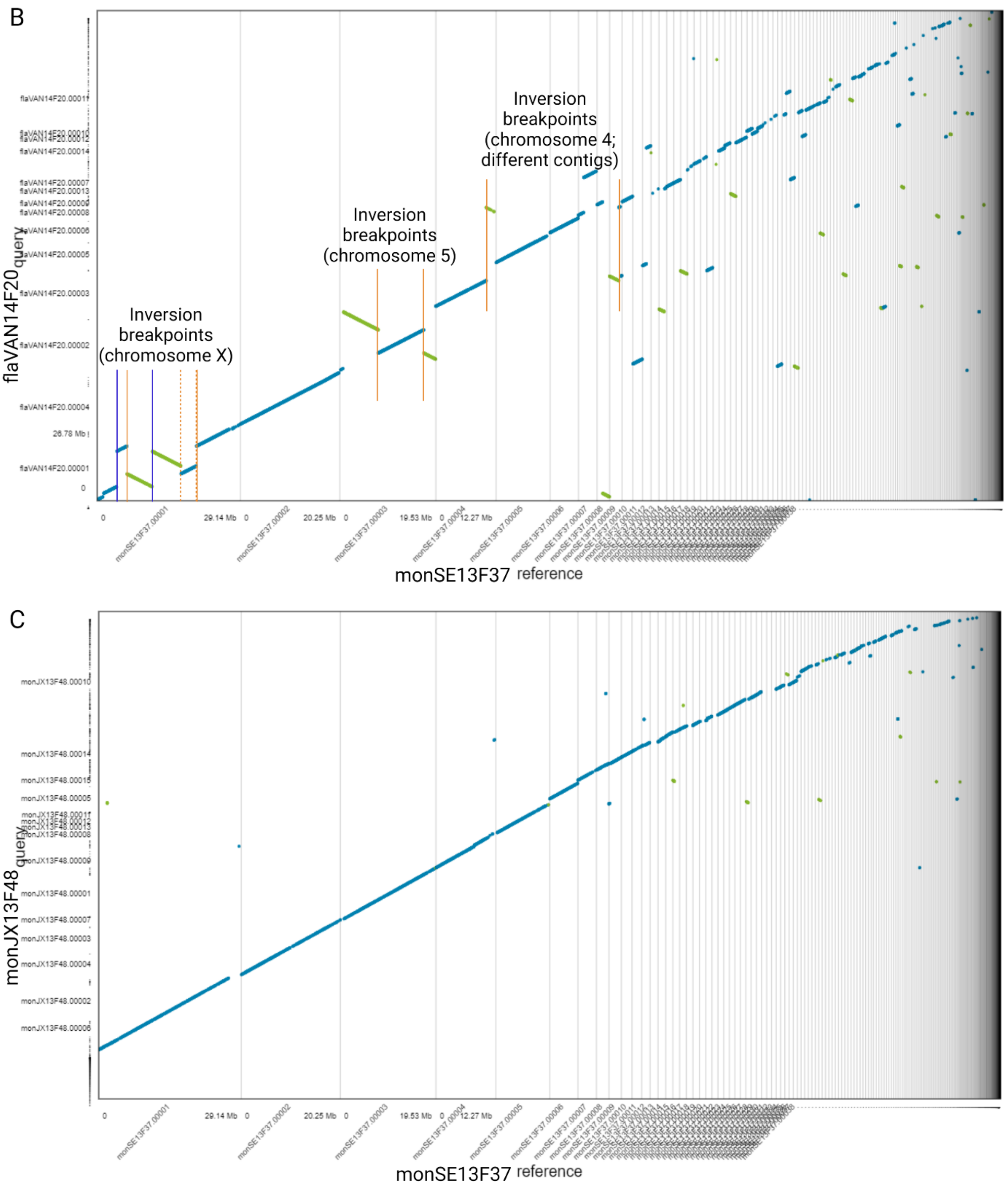

Figure S6. Nucmer alignments of the MUMmer package (Marçais et al. 2018) illustrated with Dot plots (https://dot.sandbox.bio/). *D. montana* monSE13F37 was used as a reference and (A) *D. flavomontana* flaMT13F11, (B) *D. flavomontana* flaVAN14F20, or (C) *D. montana* monJX13F48 as query. A change in color and orientation within a single pair of contig (e.g. monSE13F37.00001 and flaMT13F11.00001) indicate inverted regions. Inversions are evident only when both breakpoints are found within the same contig in both the reference and the query. Alternatively fixed inversions of *D. montana* and *D. flavomontana* are marked with blue solid lines, and orange solid and dashed lines respectively (see Table S9, Supplementary file 1).

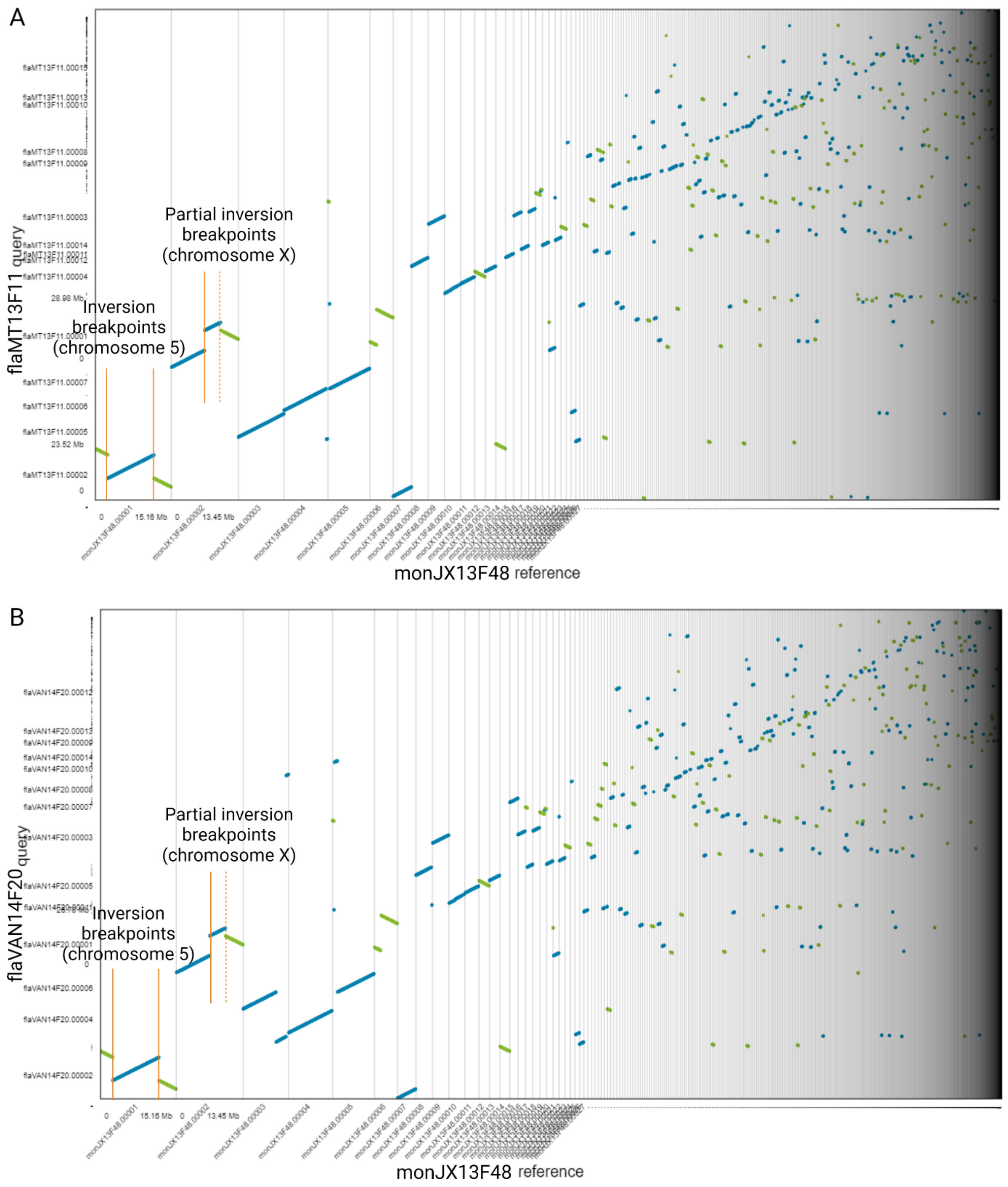
A
Partial inversion breakpoints (chromosome X)
Inversion breakpoints (chromosome 5)
flaMT13F11 query
monJX13F48 reference
B
Partial inversion breakpoints (chromosome X)
Inversion breakpoints (chromosome 5)
flaVAN14F20 query
monJX13F48 reference

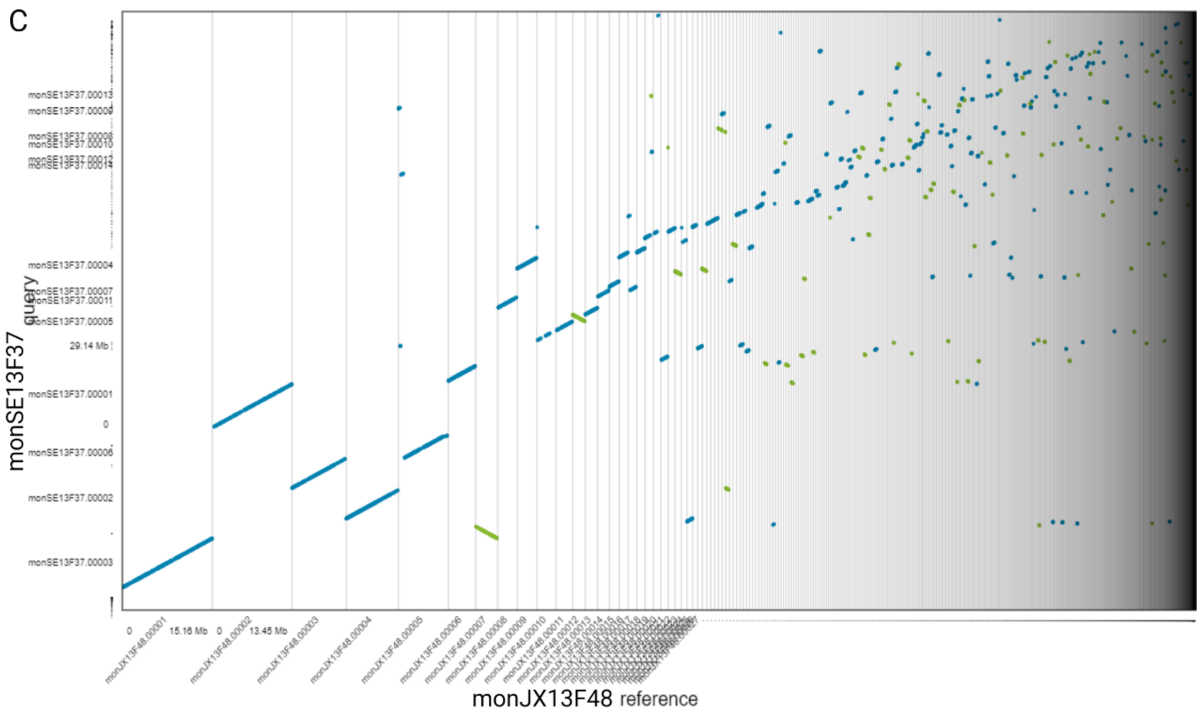

Figure S7. Nucmer alignments of the MUMmer package (Marçais et al. 2018) illustrated with Dot plots (https://dot.sandbox.bio/). *D. montana* monJX13F48 was used as a reference and (A) *D. flavomontana* flaMT13F11, (B) *D. flavomontana* flaVAN14F20, or (C) *D. montana* monSE13F37 as query. For guidance on the plot see Fig. S6.

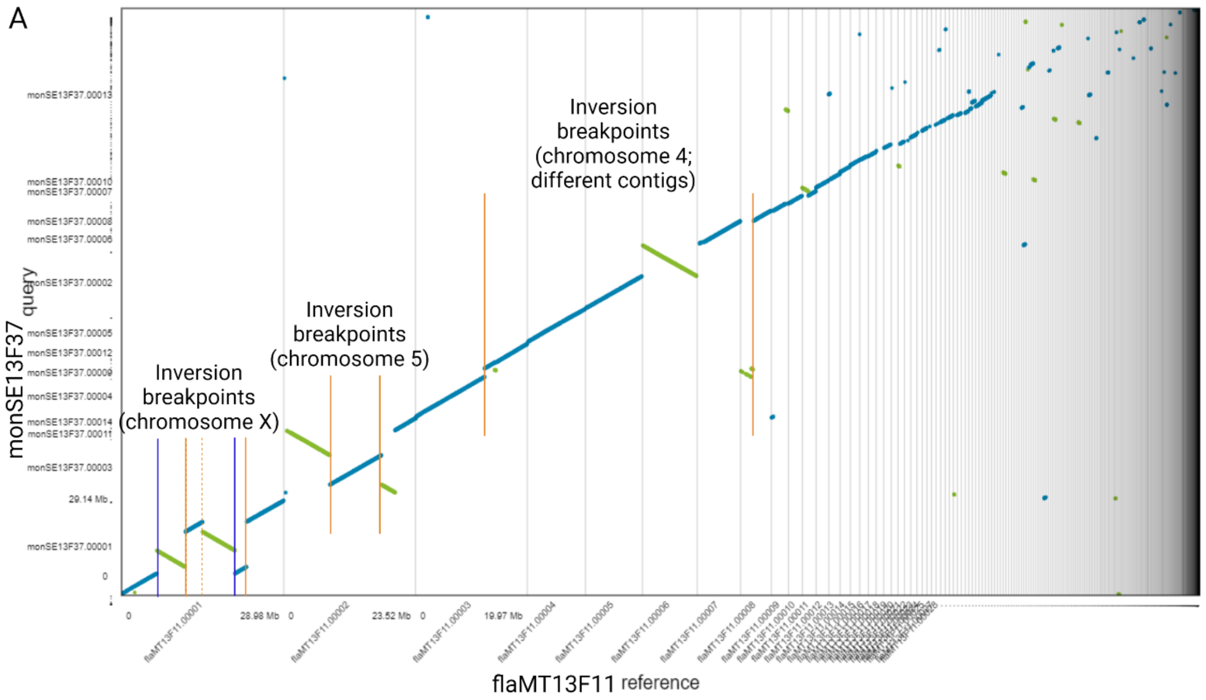

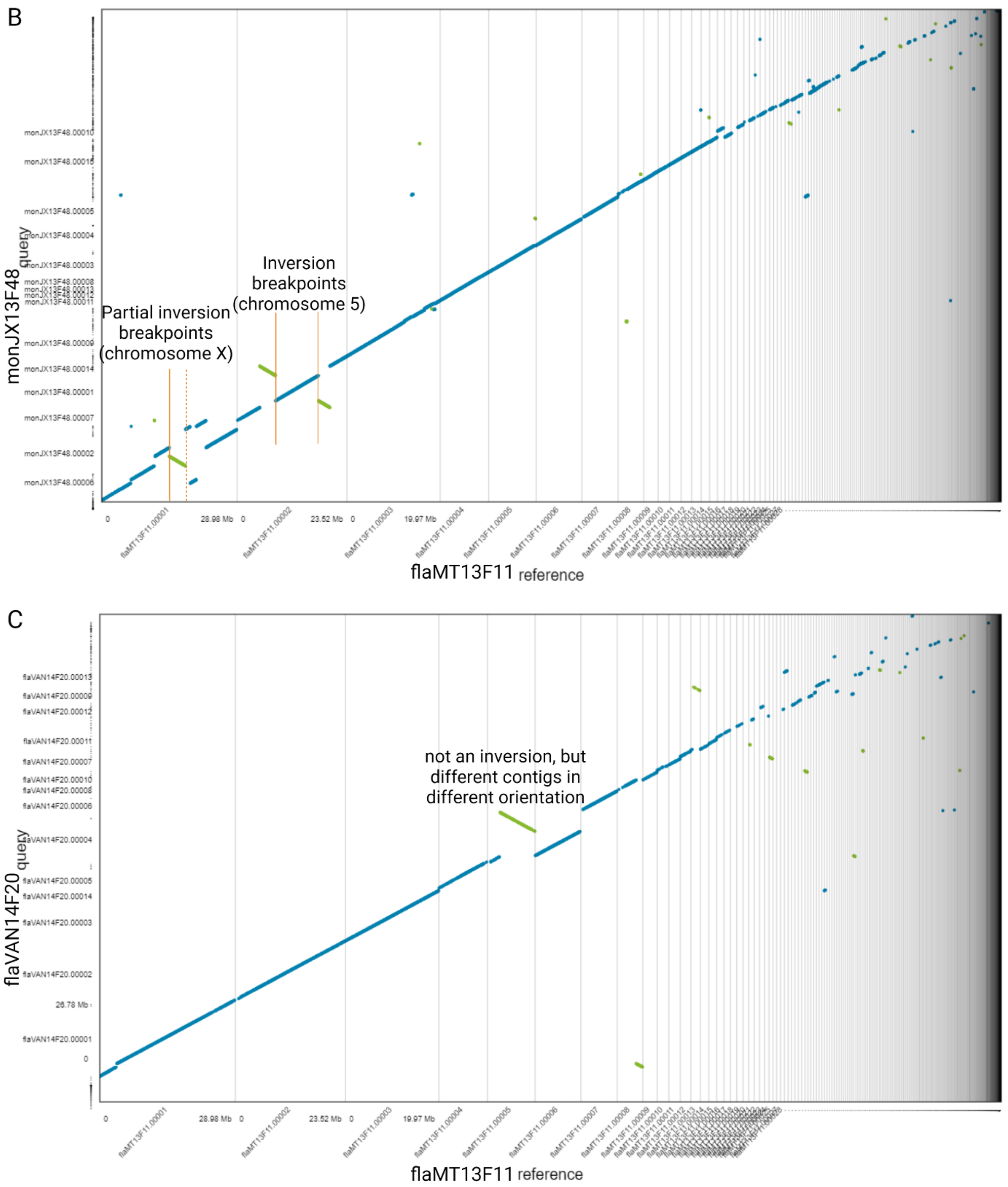

Figure S8. Nucmer alignments of the MUMmer package (Marçais et al. 2018) illustrated with Dot plots (https://dot.sandbox.bio/). *D. flavomontana* flaM13F11 was used as a reference and (A) *D. montana* monSE13F37, (B) *D. montana* monJX13F48, and (C) *D. flavomontana* flaVAN14F20 as query. For guidance on the plot see Fig. S6.

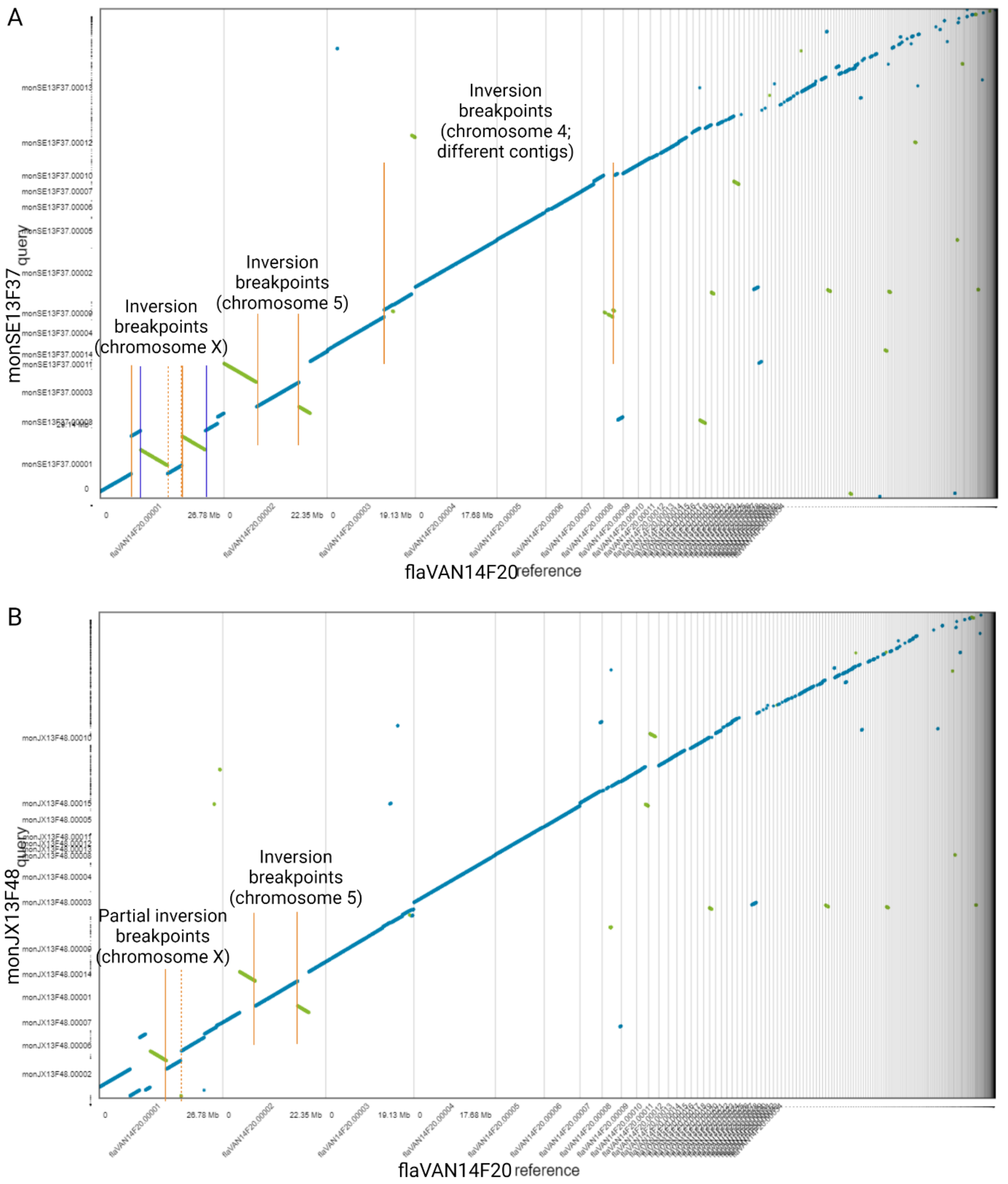
A
Inversion breakpoints (chromosome 4; different contigs)
Inversion breakpoints (chromosome 5)
Inversion breakpoints (chromosome X)
monSE13F37 query
flaVAN14F20 reference
B
Inversion breakpoints (chromosome 5)
Partial inversion breakpoints (chromosome X)
monJX13F48 query
flaVAN14F20 reference

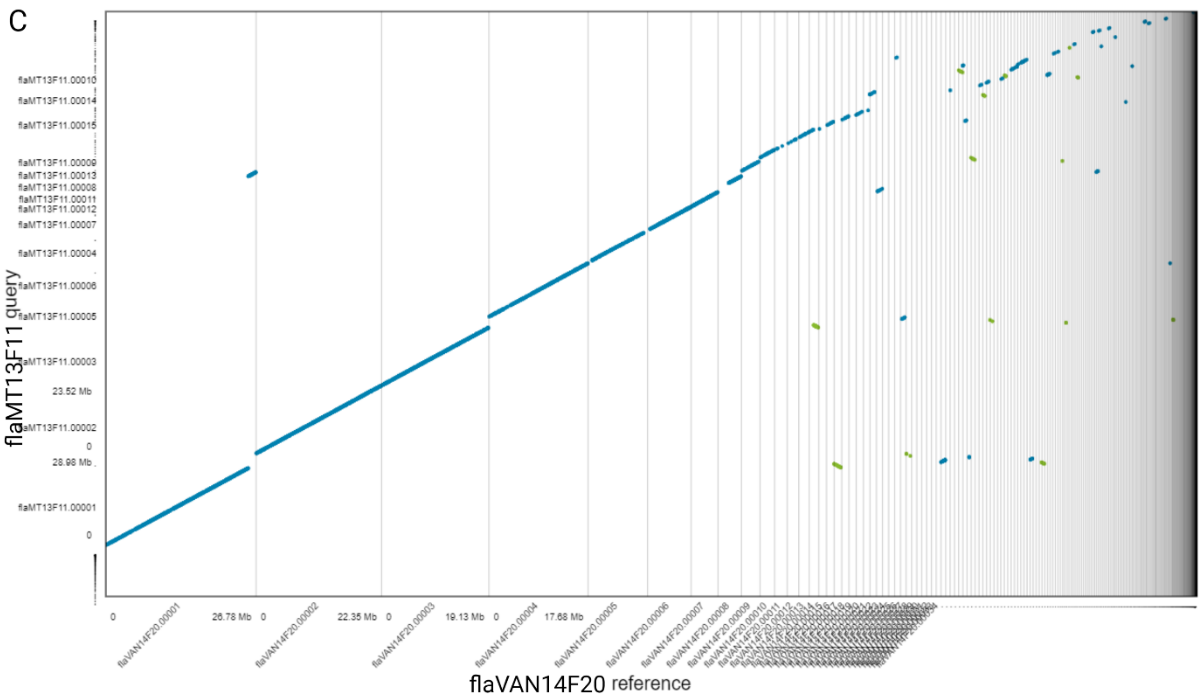

Figure S9. Nucmer alignments of the MUMmer package (Marçais et al. 2018) illustrated with Dot plots (https://dot.sandbox.bio/). *D. flavomontana* flaVAN14F20 was used as a reference and (A) *D. montana* monSE13F37, (B) *D. montana* monJX13F48, or (C) *D. flavomontana* flaMT13F11 as query. For guidance on the plot see Fig. S6.

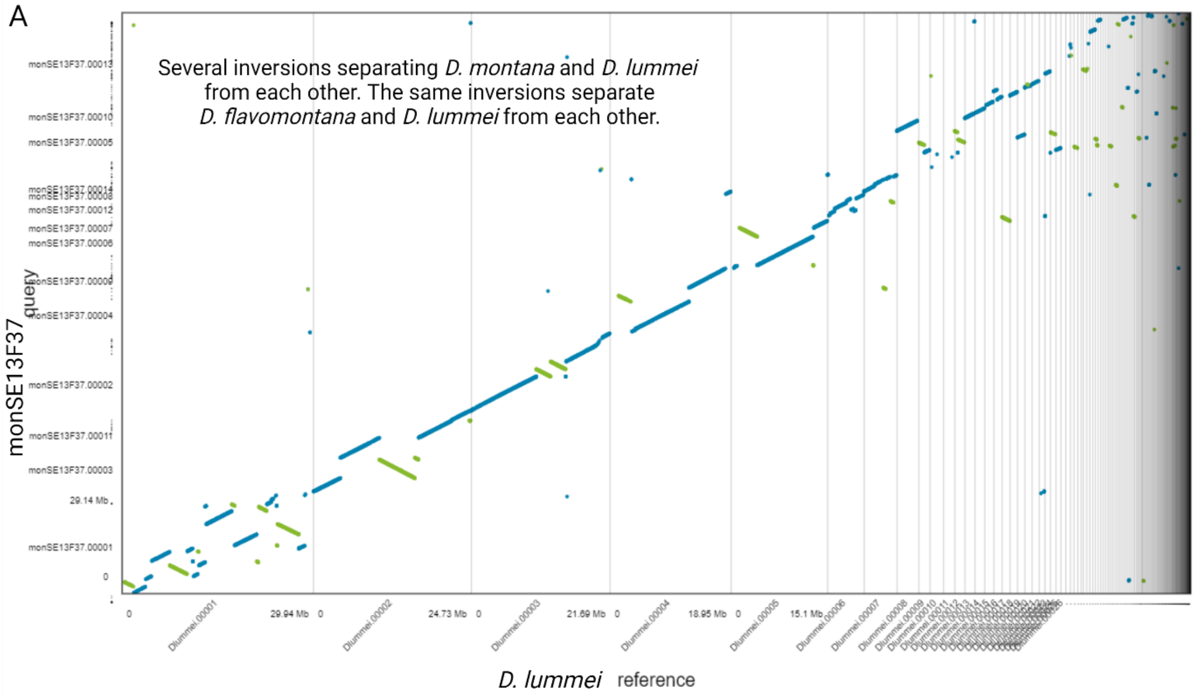

B

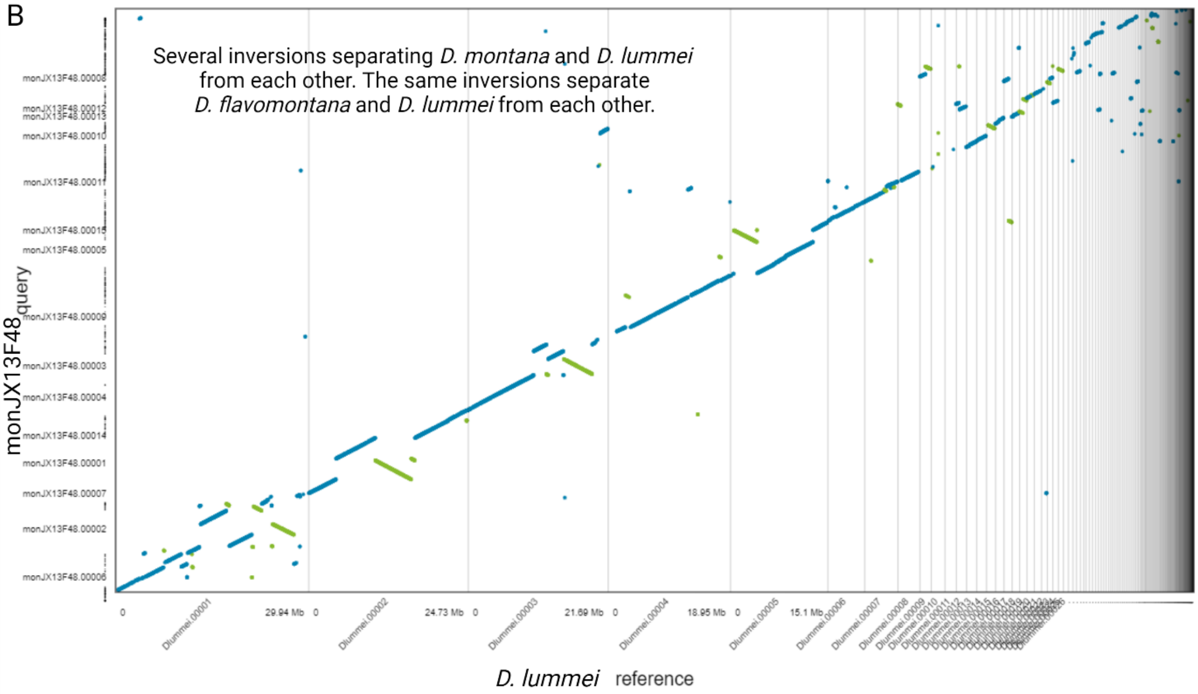
Several inversions separating D. montana and D. lummei from each other. The same inversions separate D. flavomontana and D. lummei from each other.
monJX13F48 query
D. lummei reference
29.94 Mb
24.73 Mb
21.69 Mb
18.95 Mb
15.1 Mb

C

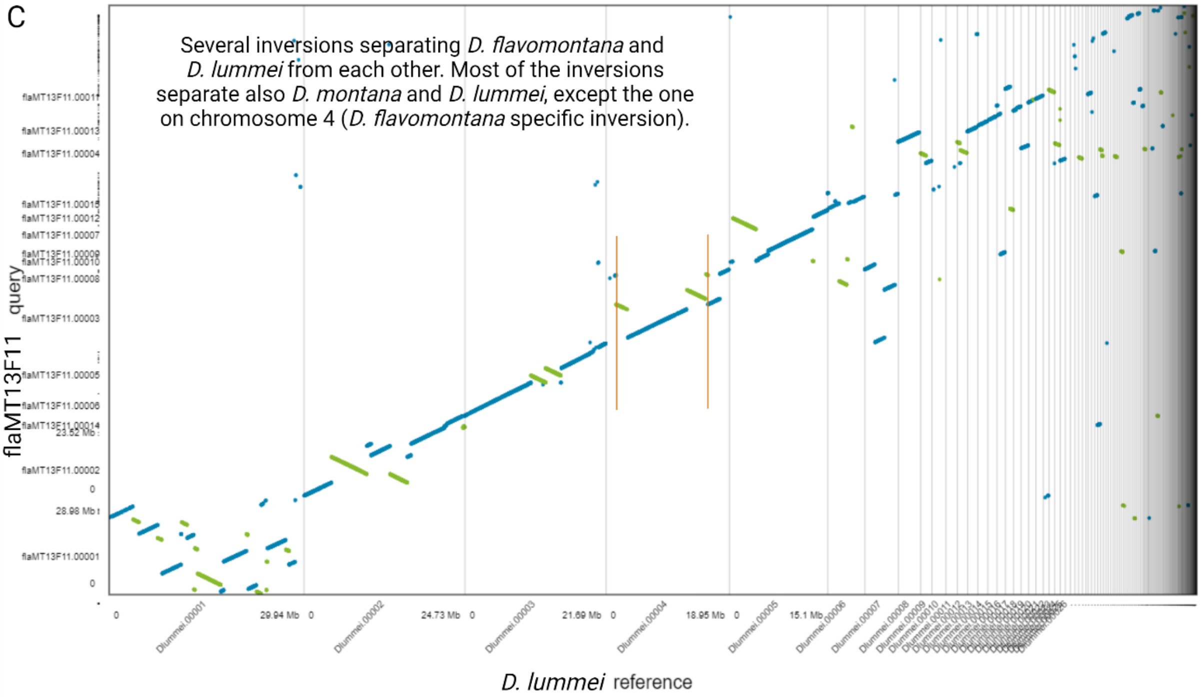
Several inversions separating D. flavomontana and D. lummei from each other. Most of the inversions separate also D. montana and D. lummei, except the one on chromosome 4 (D. flavomontana specific inversion).
flaMT13F11 query
D. lummei reference
29.94 Mb
24.73 Mb
21.69 Mb
18.95 Mb
15.1 Mb

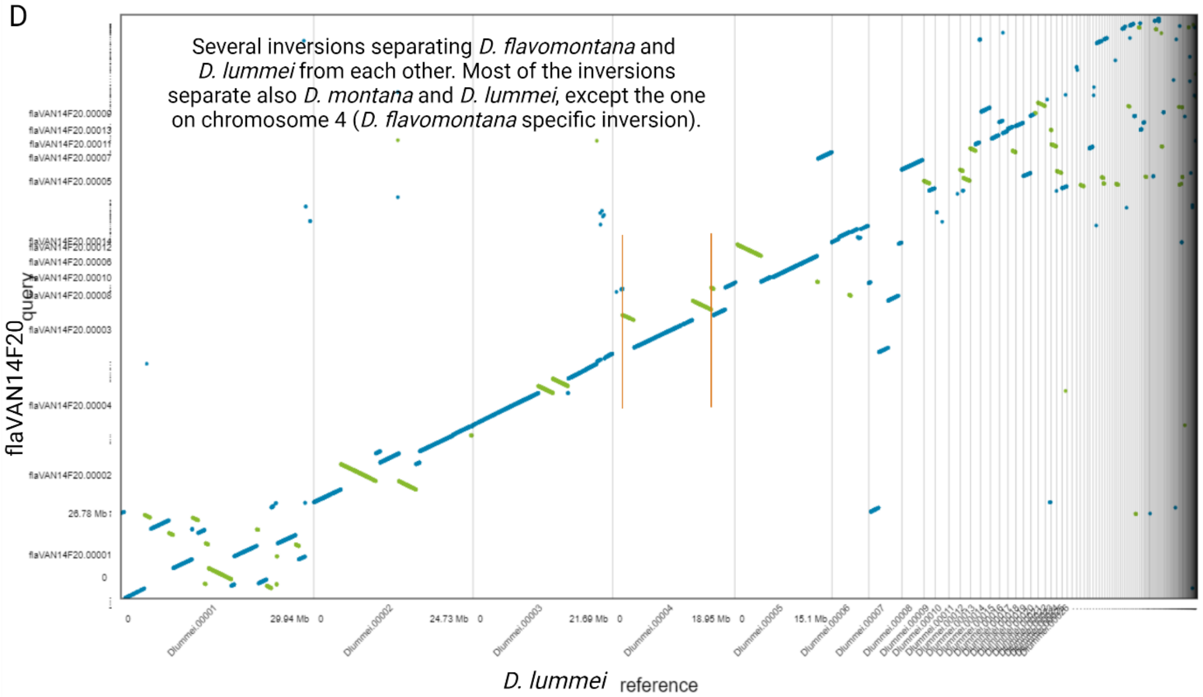

Figure S10. Nucmer alignments of the MUMmer package (Marçais et al. 2018) illustrated with Dot plots (https://dot.sandbox.bio/). *D. lummei* was used as a reference and (A) *D. montana* monSE13F37, (B) *D. montana* monJX13F48, or (C) *D. flavomontana* flaMT13F11, or (D) *D. flavomontana* flaVAN14F20 as query. For guidance on the plot see Fig. S6.

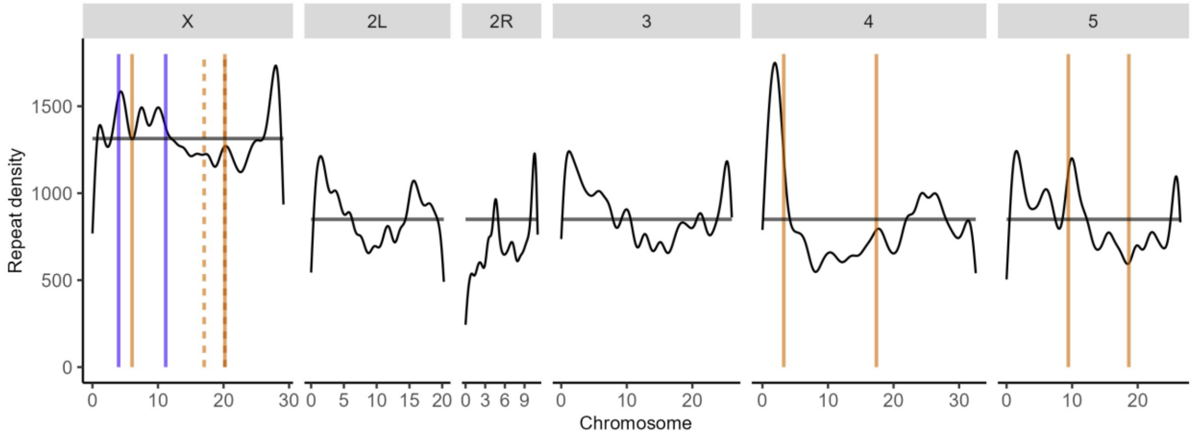

Figure S11. Density of repetitive sequences across the genome. Breakpoints of alternatively fixed inversions of *D. montana* and *D. flavomontana* are marked with solid blue, and solid and dashed orange lines, respectively (see Fig. S5). Black horizontal lines represent the mean repeat density for the X chromosome and autosomes.

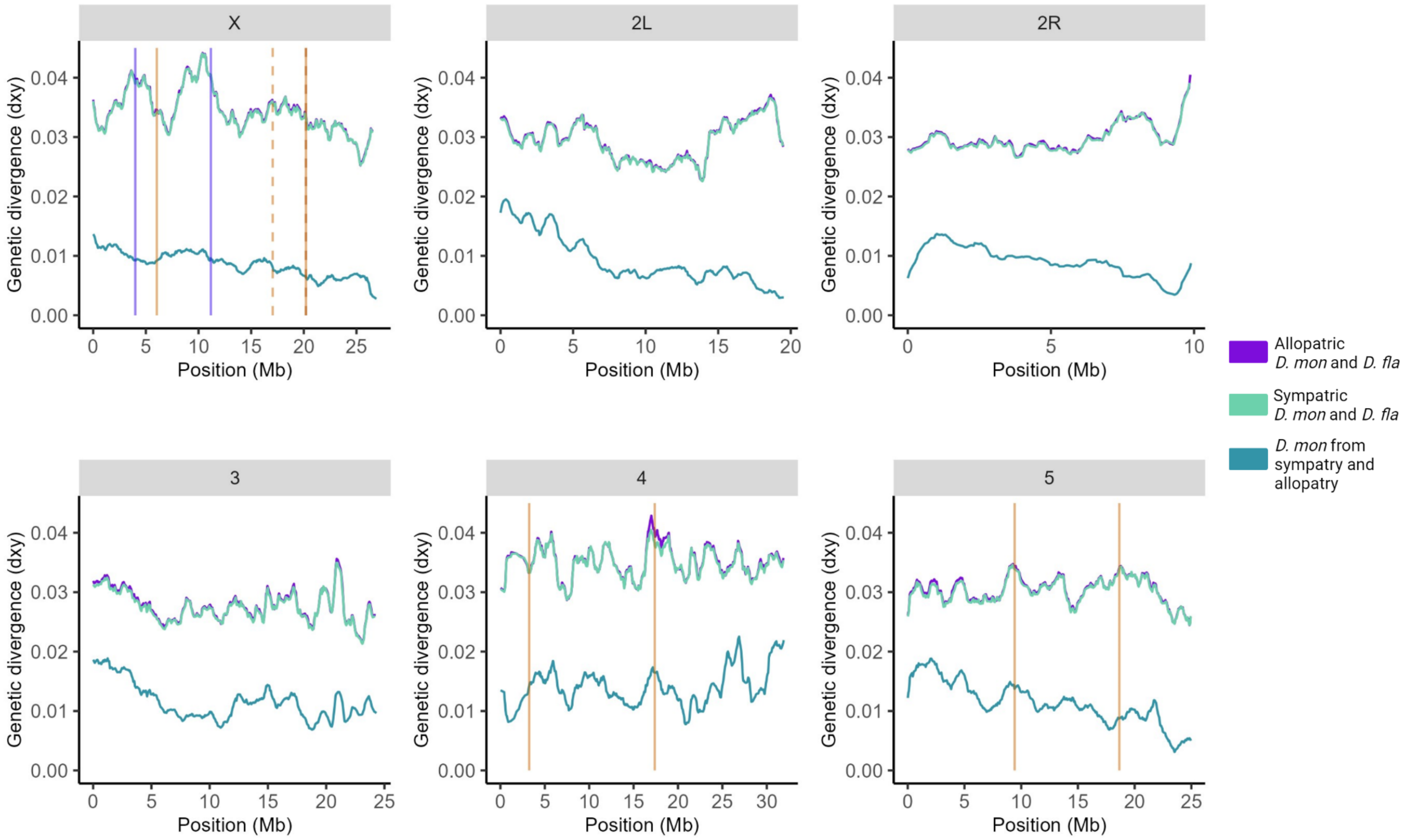

Figure S12. Genetic divergence (measured as d_{XY}) for different chromosomes (including intergenic regions) in sliding windows (window size 10 000 blocks, step size 1 000 blocks, block length 64b) for allopatric and sympatric comparisons of *D. montana* and *D. flavomontana* (interspecific), and for *D. montana* originating from allopatry and sympatry (intraspecific). Vertical lines represent breakpoints of different inversions (see Fig. S5): blue solid lines, and orange solid and dashed lines indicate alternatively fixed inversions of *D. montana* and *D. flavomontana*, respectively.

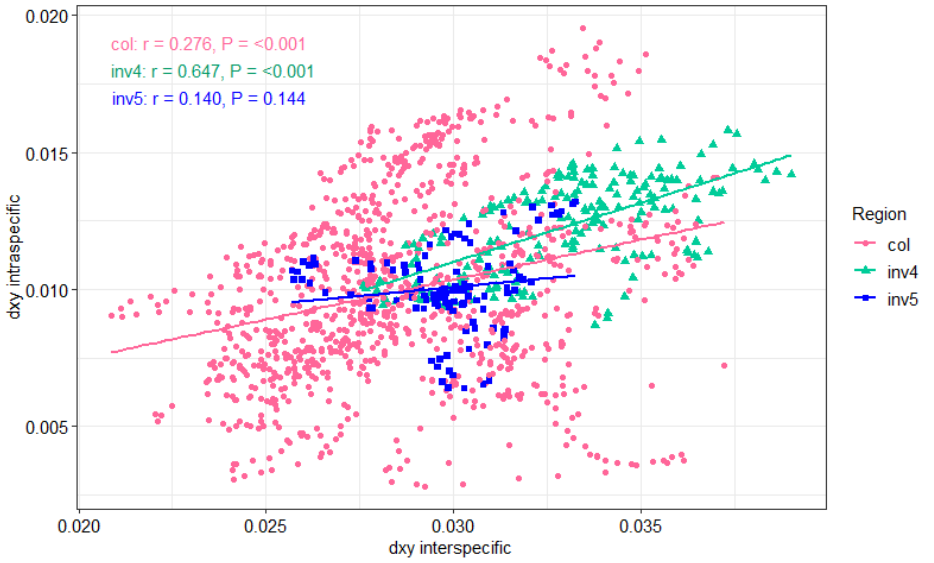

Figure S13. Pearson correlation analysis to test if correlation between interspecific and intraspecific d_{xy} is greater for inverted autosomal regions compared to colinear background. Lines represent linear regressions.

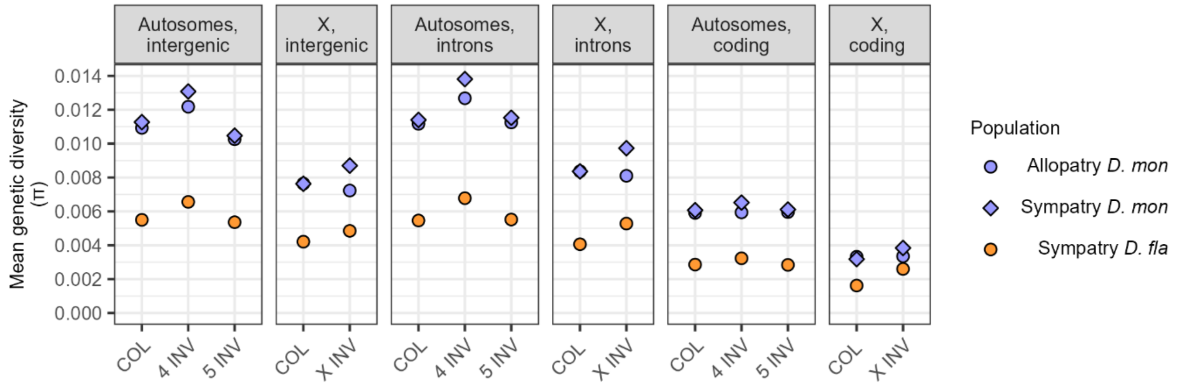

Figure S14. Mean genetic diversity (π) separately for intergenic, intronic and coding sequences, for autosomal and X chromosomal colinear (COL) and inverted (INV) chromosomal regions, as well as for *D. montana* originating from allopatric and sympatric populations and *D. flavomontana* originating from sympatric populations.

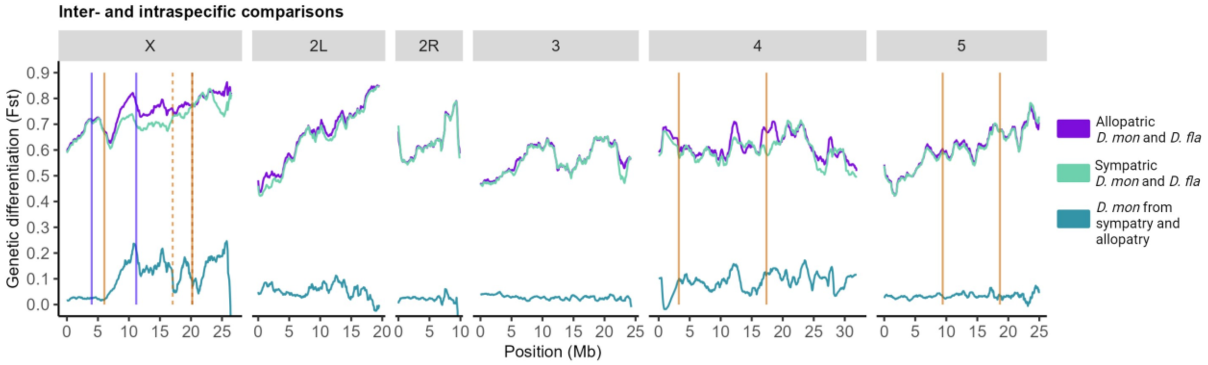

Figure S15. Genetic differentiation (measured as F_{ST}) across the genome (including intergenic regions) in sliding windows (window size 5,000 blocks, step size 500 blocks, block length 64b) for allopatric and sympatric comparisons of *D. montana* and *D. flavomontana* (interspecific), and for *D. montana* originating from allopatry and sympatry (intraspecific). Vertical lines represent breakpoints of different inversions (see Fig. S5): blue solid lines, and orange solid and dashed lines indicate alternatively fixed inversions of *D. montana* and *D. flavomontana*, respectively.

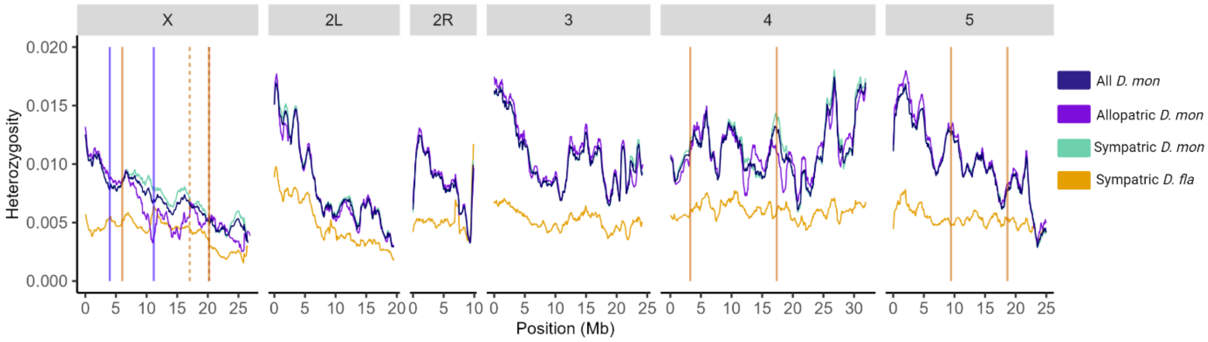

Figure S16. Heterozygosity across the genome (including intergenic regions) in sliding windows (window size 5,000 blocks, step size 500 blocks, block length 64b) separately for *D. montana* and *D. flavomontana* originating from sympatric and allopatric populations. Vertical lines represent breakpoints of different inversions (see Fig. S5): blue solid lines, and orange solid and dashed lines indicate alternatively fixed inversions of *D. montana* and *D. flavomontana*, respectively.

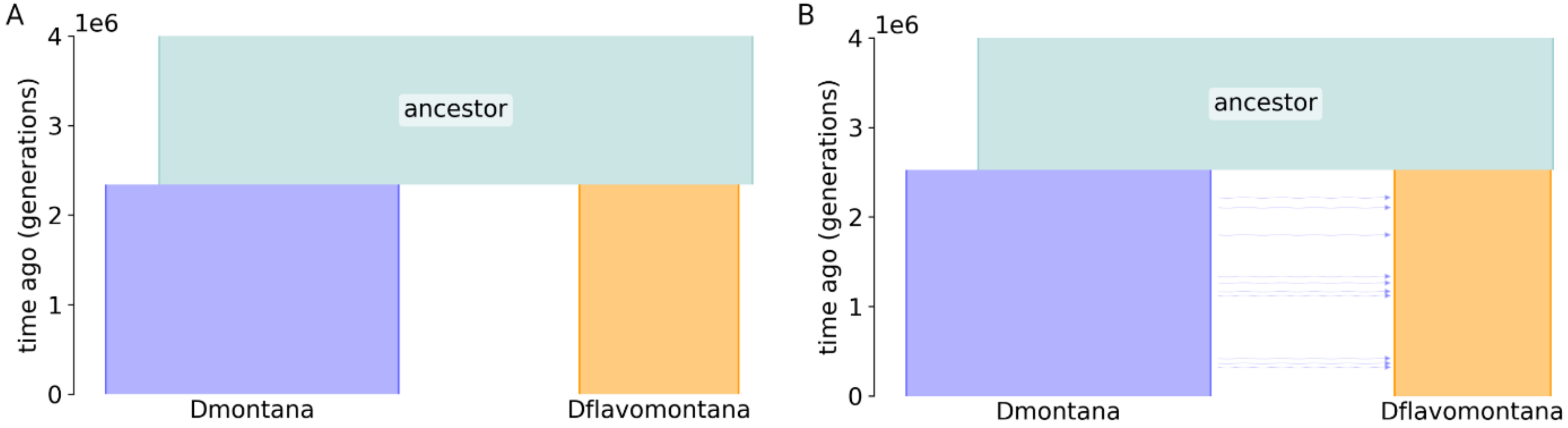

Figure S17. Sketches of the demographic models. The block widths indicate relative effective population sizes (N_e) of the ancestral population and its two descendants *D. montana* and *D. flavomontana*. (A) This model assumes no gene flow, i.e. strictly allopatric speciation (DIV). (B) The isolation with migration (IM) model allows unidirectional gene flow at rate *M* migrants per generation in this case from *D. montana* to *D. flavomontana*. Figures were produced with demesdraw (Gower et al. 2022).

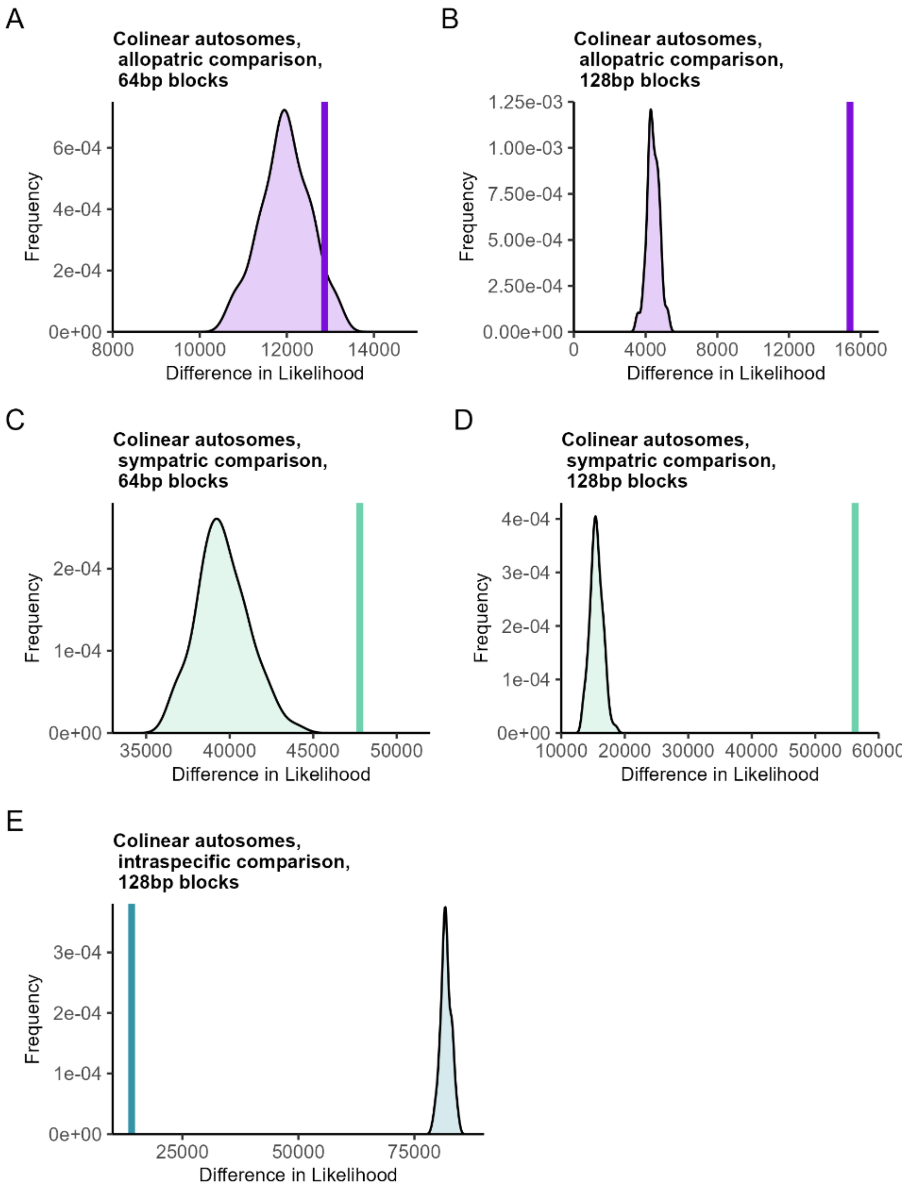

Figure S18. Datasets simulated under the strict divergence model (DIV) were fitted to both the DIV model (true model) and the IM model (wrong model), and the null distribution of ΔlnL was compared to the observed ΔlnL (vertical line). If the observed ΔlnL falls outside that null distribution, the IM model fits significantly better than the DIV model. The data were analysed using the *D. montana* genome as a reference. The analysis was performed for colinear autosomal regions of (A) allopatric comparison with block size 64b, (B) allopatric comparison with block size 128b, (C) sympatric comparison with block size 64b, (D) sympatric comparison with block size 128b, (E) intraspecific comparison with block size 128b.

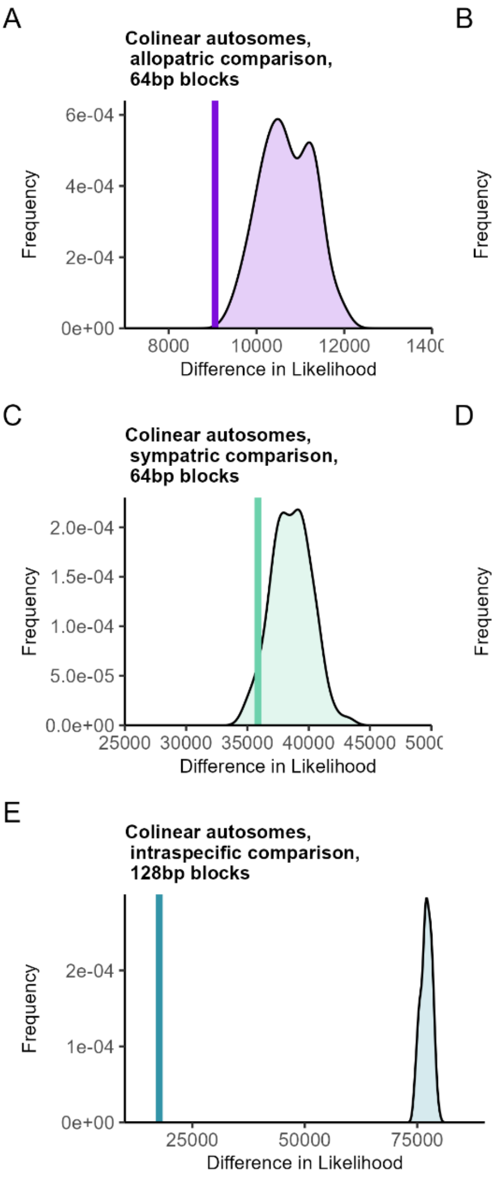

Figure S19. Datasets simulated under the strict divergence model (DIV) were fitted to both the DIV model (true model) and the IM model (wrong model), and the null distribution of ΔlnL was compared to the observed ΔlnL (vertical line). If the observed ΔlnL falls outside that null distribution, the IM model fits significantly better than the DIV model. The data were analysed using the *D. flavomontana* genome as a reference. The analysis was performed for colinear autosomal regions of (A) allopatric comparison with block size 64b, (B) allopatric comparison with block size 128b, (C) sympatric comparison with block size 64b, (D) sympatric comparison with block size 128b, (E) intraspecific comparison with block size 128b.

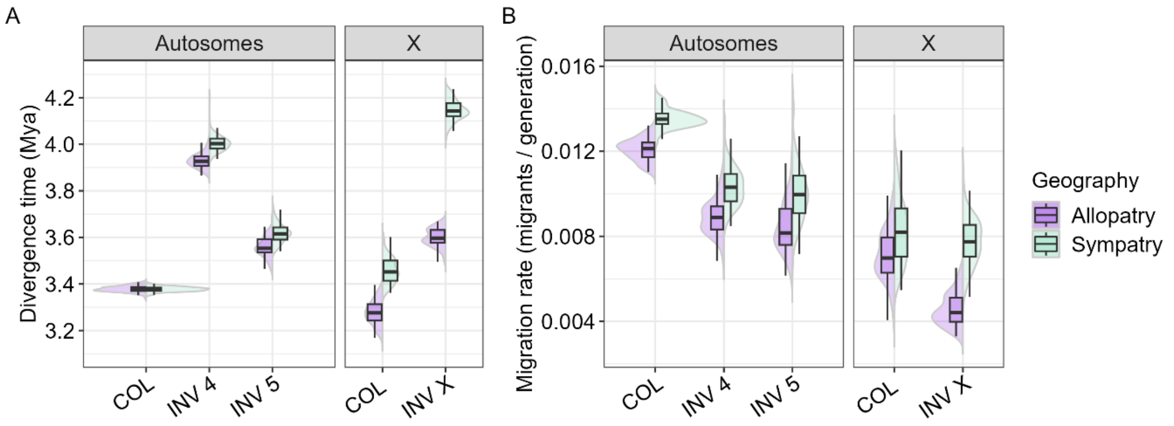

Figure S20. Estimates of (A) split times and (B) migration rates between *D. montana* and *D. flavomontana* for different chromosome partitions and for allopatric (purple) and sympatric (green) comparisons. Confidence intervals were estimated using 100 parametric bootstrap simulations (with recombination) under the best-fit IM model. Boxplot whiskers represent ±1.5×IQR; the distributions of estimates are shown as smoothed histograms.

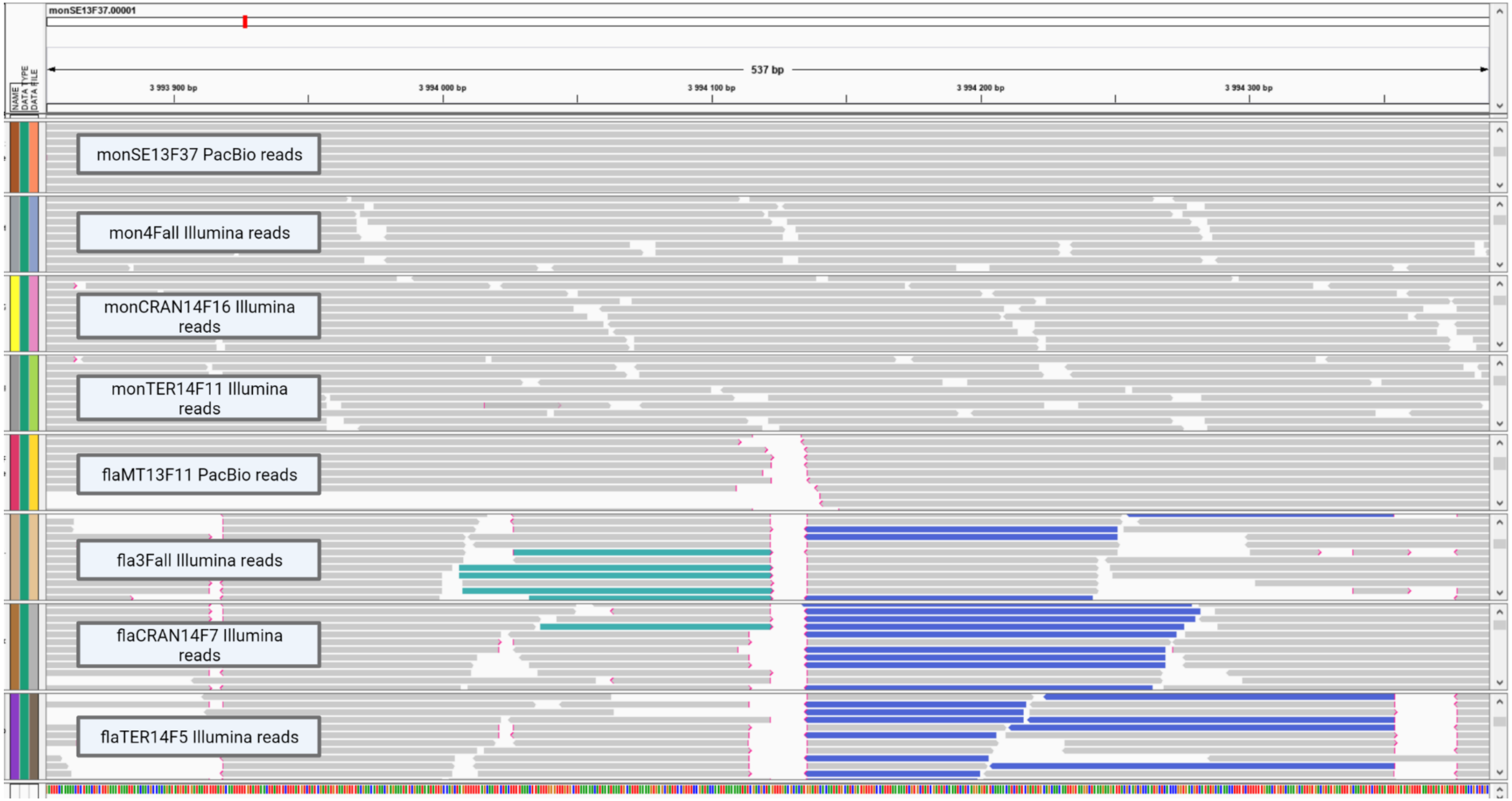

Figure S21. Example plot of an inversion breakpoint illustrated with Integrative Genomics Viewer (IGV) v2.8.0 (Thorvaldsdóttir et al. 2013) using both long read PacBio and short read Illumina data. Both long and short reads of *D. flavomontana* samples, mapped against *D. montana* genome, are split (red tip of the read) or in reversed orientation (blue and green reads) due to alternatively fixed inversion of the species.

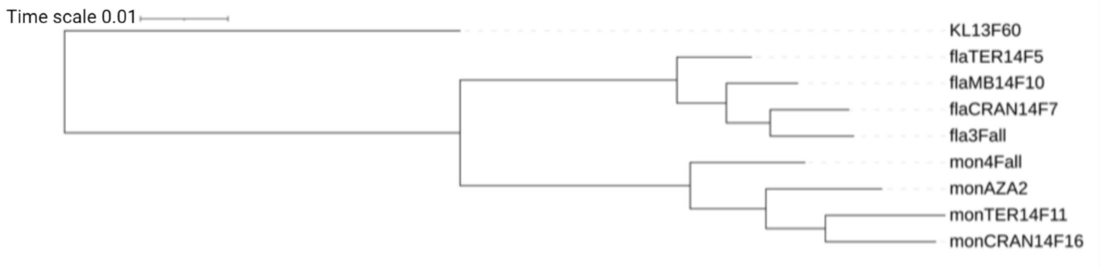

Figure S22. Phylogenetic tree produced by Orthofinder. Prefix "mon" and "fla" before a fly strain ID refer to *D. montana* and *D. flavomontana*, respectively. *D. littoralis* sample (KL13F60) was used as an outgroup in the tree. Details of the samples are given in Table S14.

Supplementary references

Fick SE, Hijmans RJ. 2017. WorldClim 2: new 1-km spatial resolution climate surfaces for global land areas. *Int. J. Climatol.* 37:4302–4315.

Gower G, Ragsdale AP, Bisschop G, Gutenkunst RN, Hartfield M, Noskova E, Schiffels S, Struck TJ, Kelleher J, Thornton KR. 2022. Demes: A standard format for demographic models. *Genetics* 222:iyac131.

Laetsch DR, Blaxter ML. 2017. BlobTools: Interrogation of genome assemblies. *F1000Research 2017* 6:1–16.

Marçais G, Delcher AL, Phillippy AM, Coston R, Salzberg SL, Zimin A. 2018. MUMmer4: A fast and versatile genome alignment system. *PLoS Comput. Biol.* 14:1–14.

Thorvaldsdóttir H, Robinson JT, Mesirov JP. 2013. Integrative Genomics Viewer (IGV): high-performance genomics data visualization and exploration. *Brief. Bioinform.* 14:178–192.
